# Functionalized Nitroimidazole Scaffold Construction and Their Pharmaceutical Applications: A 1950–2021 Comprehensive Overview

**DOI:** 10.3390/ph15050561

**Published:** 2022-04-30

**Authors:** Ria Gupta, Sumit Sharma, Rohit Singh, Ram A. Vishwakarma, Serge Mignani, Parvinder Pal Singh

**Affiliations:** 1 Natural Product & Medicinal Chemistry Division, CSIR-Indian Institute of Integrative Medicine (CSIR-IIIM), Canal Road, Jammu 180001, India; riagupta518@gmail.com (R.G.); sumit.iiim23@gmail.com (S.S.); singhrohit737@gmail.com (R.S.); ram@iiim.ac.in (R.A.V.); serge_mignani@orange.fr (S.M.); 2 Academy of Scientific & Innovative Research, Gaziabad 201002, India; 3 PRES Sorbonne Paris Cité, CNRS UMR 860, Laboratoire de Chimie et de Biochimie Pharmacologiques et Toxicologique, 45, rue des Saints Peres, Université Paris Descartes, 75006 Paris, France; 4 CQM—Centro de Química da Madeira, MMRG, Universidade da Madeira, Campus da Penteada, 9020-105 Funchal, Portugal

**Keywords:** nitroimidazole, synthesis, antibiotic, clinical use

## Abstract

Nitroimidazole represents one of the most essential and unique scaffolds in drug discovery since its discovery in the 1950s. It was K. Maeda in Japan who reported in 1953 the first nitroimidazole as a natural product from *Nocardia mesenterica* with antibacterial activity, which was later identified as Azomycin **1** (2-nitroimidazole) and remained in focus until now. This natural antibiotic was the starting point for synthesizing numerous analogs and regio-isomers, leading to several life-saving drugs and clinical candidates against a number of diseases, including infections (bacterial, viral, parasitic) and cancers, as well as imaging agents in medicine/diagnosis. In the present decade, the nitroimidazole scaffold has again been given two life-saving drugs (Delamanid and Pretomanid) used to treat MDR (multi-drug resistant) tuberculosis. Keeping in view the highly successful track-record of the nitroimidazole scaffold in providing breakthrough therapeutic drugs, this comprehensive review focuses explicitly on presenting the activity profile and synthetic chemistry of functionalized nitroimidazole (2-, 4- and 5-nitroimidazoles as well as the fused nitroimidazoles) based drugs and leads published from 1950 to 2021. The present review also presents the miscellaneous examples in each class. In addition, the mutagenic profile of nitroimidazole-based drugs and leads and derivatives is also discussed.

## 1. Introduction

**Figure 1:**
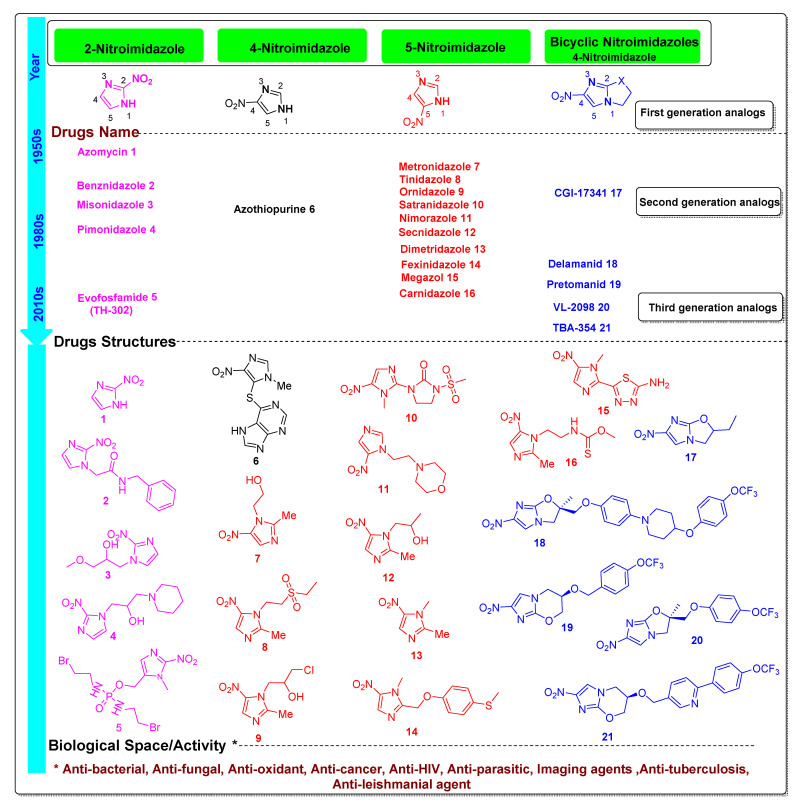
Timeline and selected related chemical structures of nitroimidazole series.

During the past decade, the attrition rate of drug development candidates reaching the market has been decreased and has become one of the significant challenges in pharmaceutical research and drug development (R&D). Several issues have arisen to explain the decrease in pharmaceutical industry productivity, including the non-optimal physicochemical properties of hits, leads and clinical candidates, affecting their absorption, distribution, metabolism, elimination, and toxicity (ADMET) profiles, and consequently, their drug-like properties [1,2]. Several strategies have been developed to solve these issues, such as (1) target-based approaches (e.g., fragment-based approach, drug-design approach) and (2) phenotypic screening approach, as well as the scaffold hopping approach and functionalization of the known scaffold [3]. In this direction, the nitroimidazole scaffold was discovered in the early 1950s and was reported for anti-bacterial potential, which was highly exploited regarding its chemistry and pharmacological space, and thereafter, several drugs and clinical candidates have been generated. Figure 1 below shows the timeline and selected related chemical structures of the nitroimidazole series and their therapeutic applications, clearly showing the interest of drug development of this scaffold [1].

**Figure 2:**
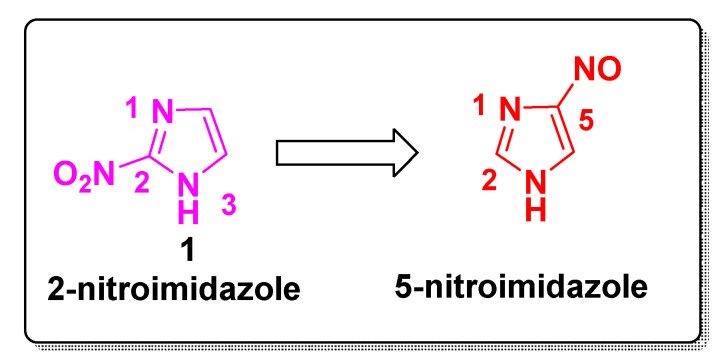
Chemical structure of 2-nitroimidazole and 5-nitroimidazole.

The journey of nitroimidazole in drug discovery started with a discovery made by Maeda et al. in 1953 with the isolation of a compound from the culture of bacteria *Nacardia mesenterica* [4]. In 1955, Nakamura established its structure as 2-nitroimidazole [5]. Later, 2-nitroimidazole was also isolated from other bacterial strains such as *Streptomyces, Trichomonas*, etc. Azomycin **1** has shown potent activity against trichomoniasis [1], a sexually transmitted parasitic disease caused by *Trichomonas vaginalis*, which inspired the researchers of RhÔne-Poulenc for its derivatization, but all attempts toward its synthesis were unsuccessful. RhÔne-Poulenc researchers shifted their attention to the synthesis of 5-nitroimidazole regio-isomers, which fortuitously led to the identification of Metronidazole **7** and other related drugs, discussed in depth in upcoming sections (Figure 2).

**Figure 3:**
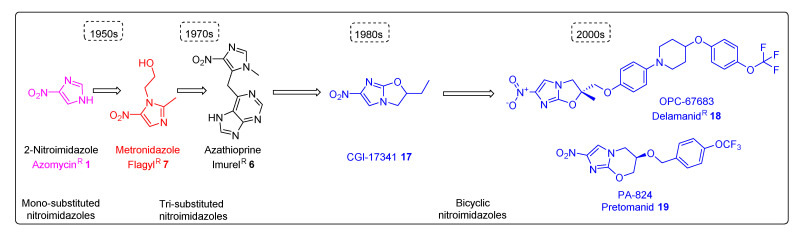
Transition from simple nitroimidazole to functionalized and optimized nitroimidazole-based drug candidates.

The 5-nitroimidazole scaffold [6] was the starting point to the synthesis of numerous analogs, and one among such is Metronidazole **7** ((Flagyl^®^, Rhône-Poulenc, France), which has shown potent anti-protozoal activity and lesser toxicity than Azomycin **1** (Figure 3) [7]. Metronidazole **7** was the first systemically active medicine against *Trichomonas vaginalis* and *Trichomonas foetus*. Currently, Metronidazole **7** is used to treat bacterial *vaginosis*, *trichomonas, amebiasis*, and non-sporing anaerobic bacterial infections caused by *Bacteroids fragilis* [8]. The 5-nitroimidazole moiety is well known for its broad spectrum of anti-infectious activity [9]. A specific review on the anti-infectious activity of 5-nitroimidazole has been emphasized by Crozet et al [6]. For instance, Metronidazole **7** (Flagyl^®^, developed by Rhône-Poulenc, France), Tinidazole **8** (Fasigyne^®^, developed by Pfizer, Brooklyn, NY, USA), Ornidazole **9** (Tiberal^®^, developed by Hoffmann-La Roche, Basel, Switzerland), Satranidazole **10**, Nimorazole **11** (Nitrimidazine, Naxogin^®^, developed by Carlo Erba, Cornaredo, Italy), Secnidazole **12** (Secnol^®^, developed by Symbiomix, Newark, NJ, USA), and Dimetridazole **13** (Emtryl^®^, developed by Rhône-Poulenc, France) are other members of this class of drugs commonly used in medicine. Apart from these, Fexinidazole **14** (Sanofi, France & DNDi) represents another 5-nitroimidazole-based drug [10], which has replaced the old and highly problematic treatment techniques and is an oral, safe and effective short-course treatment for human African trypanosomiases. Fexinidazole **14** is free of genetic toxicity in mammalian cells [1]. Other 5-nitroimidazole-based candidates are Megazol **15** (discovered by American Cyanamid Company, Bridgewater Township, NJ, USA), used against protozoan infections and Carnidazole **16** (Spartrix, Pantrix, Gambamix^®^, developed by Janssen, Pharmaceutica NV, Beerse, Belgium) used in the veterinary field. 

Apart from 5-nitroimidazole, 2-nitroimidazole analogs have also utilised in many drugs, and representative examples as drugs are Benznidazole **2** (Rochagan, developed by Laboratório Farmacêutico do Estado de Pernambuco, Recife, Brazil) [11], Misonidazole **3** (designed by Roche, Branchburg, NJ, USA), Pimonidazole **4** (Hypoxyprobe, Roche, NJ, USA) [12] and Evofosfamide **5** (TH-302, Threshold Pharmaceuticals Inc., San Francisco, CA, USA) [13]. This reliable tumor candidate underwhelms in phase III studies.

In the last decade, nitroimidazole derivates belonging to bicyclic-fused nitroimidazole has also shown great potential in TB drug discovery [14,15]. The first fused nitroimidazole reported was CGI-17341 **17** (Hindustan Giba-Geigy, Figure 1), which displayed in vitro and in vivo antitubercular activities [16]. CGI-17341 **17** was found to be active against multidrug-resistant strains. Unfortunately, the development of this compound was discontinued due to its mutagenic side effects [17,18]. Almost 10 years after, two new bicyclic nitroimidazoles were developed, OPC-67683 **18** (Delamanid, by Otsuka Pharmaceuticals, Tokyo, Japan) [1] and PA-824 **19** (Pretonamid, by PathoGenesis, USA and TB Alliance, New York, NY, USA) [1] without any mutagenic effects (Figure 1). Delamanid was approved for the pulmonary multidrug-resistant TB by the European Medicines Agency (EMA) in 2014 in adult patients [19]. In contrast, Pretomanid **19** was approved in 2019 for the treatment of TB. Recently, the Global Alliance for TB drug development and the University of Auckland advanced in Phase I of TBA-6354 **21** but showed neurotoxicity in healthy volunteers [20]. This class of nitroimidazole derivatives has also shown promising potential in leishmanasis drug discovery, which needs an effective oral treatment option [21]. This good profile has also attracted significant interest. Another structure in this class, VL-2098 **20**, Ref. [17] has also been reported.

Nitroimidazole represents a unique scaffold in drug discovery and development, has given many successful drugs and clinical candidates, and is still being pursued for medicinal chemistry exploitation. Considering its wide and interesting biological activities, several reviews and books were published. However, most of them were focused on their pharmacological profiles [1,6,18,22,23,24,25]. There are few reviews that cover the partial synthetic aspects of this scaffold [26,27,28], and considering its wide application and the presence of diverse space around this scaffold, there should be a consolidated review covering chemistry, pharmacological and recent development. These wonderful chemical series have given numerous analogs and regio-isomers as potential drug candidates for several therapeutic conditions such as anti-bacterial, anti-cancer, anti-HIV, anti-parasitic, anti-tuberculosis, anti-leishmaniasis agents, etc., as well as imaging agents in medicine. Figure 3 summarizes the transition from simple nitroimidazole to functionalized and optimized nitroimidazole-based drug and candidates.

### Mechanism of Action of Nitroimidazole Derivatives at a Glance

**Figure 4:**
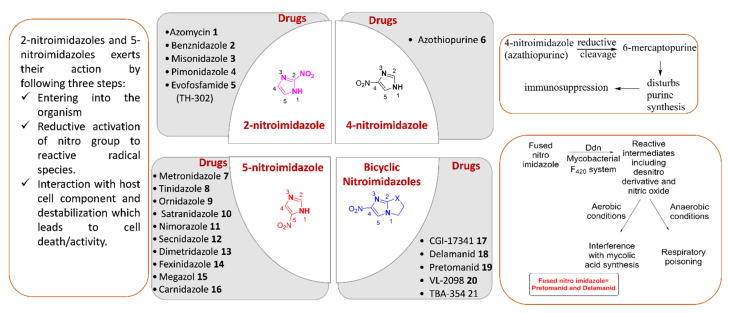
Mechanistic overview of nitroimidazole-based drugs.

These chemotherapeutic agents are known to have diverse pharmacological activities such as anti-bacterial, anti-parasitic, anti-cancer, anti-HIV, anti-tuberculosis, anti-leishmaniasis agents, etc., and imaging agents in medicine. Obviously, these molecules interact with several bio-chemical pathways of hosts and parasites. The exact mechanism of either of these drugs is not known. However, several studies assumed that the reductive bioactivation and generation of reactive intermediates are responsible for the overall effect. In general, nitroimidazole-based drugs and leads are well defined as pro-drug, and the nitro functionality is responsible for the activity. The nitro group converts into nitric oxide (NO) or a related reactive nitrogen species (RNS) by the process of reductive bioactivation, the details being recently compiled and published [29,30]. The activation and interaction of each class depend upon the conditions and diseases on focus. Each class has shown different reduction potential, and their activation depends upon the environment. Here, we briefly summarize the trends in each class (Figure 4).

Among the classes, 2-imidazole-based drugs and leads are being used as potential anti-protozoals as well as radio-sensitizers and diagnostic markers for several cancers. These activities exploit the nitro functionality of the candidate, which undergoes reduction under hypoxia and irradiation and generates the reactive intermediates that ultimately interact with important cellular components such as DNA, RNA and proteins, thus inhibiting growth. 2-Nitroimidazole-based drugs such as Benznidazole **2** show anti-protozoal activity because of the formation of free radicals and electrophilic metabolites, when its nitro group is reduced by the action of nitroreductases. The metabolites covalently bind to macromolecules of the parasite and are responsible for the trypanocidal effect and anti-protozoal effect [31]. There is one drug belonging to nitroimidazole class named as Azathiopurine **6**, which is known for its immunosuppression property. Glutathione and similar compounds of the intestinal wall, liver and red blood cells mediate the reductive cleavage of the thioether (-*S*-) due to which Azathioprine **6** converts to 6-mercaptopurine (6-MP) [32,33,34]. Azathioprine **6** interferes with purine synthesis and disrupts DNA and RNA synthesis, thus causing immunosuppression [35].

Conversely, 5-ntroimdazole-based drugs also exploit the nitro functionalities and, under reduction by several anaerobic bacteria and parasites, generate the reactive radical-based intermediates that ultimately interact with cellular components of bacteria and parasites, stopping their growth and providing therapeutic effects. The fused nitroimidazole-based drugs such as delamanid and pretomanid are known for potential anti-tubercular activity. Their anti-tubercular activity is known to be controlled by their dual mechanism of action (interference with mycolic acid synthesis and respiratory poisoning) [36]. Regarding the mechanistic study, it is assumed that both pretomanid and delamanid have similar mechanisms of action, and most of the research to understand the mechanism has been performed with Pretomanid **19**. The nitroreductase enzymes (deazaflavin-dependent nitroreductase (Ddn)) of *Mycobacterium tuberculosis* selectively bio-activate the fused nitroimidazoles via the denitrification process (releasing nitric oxide) and generate reactive intermediates; however, the bio-activation of both compounds is favored by redox cycling of deazaflavin cofactor 420, or F_420_. The mechanistic study revealed that the inhibition of mycolic acids is responsible for the killing of *Mycobacterium tuberculosis* under aerobic conditions; however, the interaction of released nitric oxide with cytochrome oxidases in the mycobacterial electron transport chain results in disruption of ATP synthesis. The overall effects are responsible for the potential activity of fused nitroimidazole-based drugs and leads. Nitro functionality of the imidazole-based drugs and candidates is also responsible for mutagenic liabilities [37], however, some existing nitroimidazole-based drugs and leads are devoid of this liability [38]. This property depends on the unique balance of electronic and steric factors, which qualifies this scaffold for providing drug candidates. The overall trend of mutagenicity among the class is also discussed in the latter part of this review.

This review surveys and analyzes the synthetic routes to prepare functionalized nitroimidazole scaffolds. This review has been divided based on the different nitroimidazole scaffold types: 2-nitroimidazole, 4-nitroimidazole, 5-nitroimidazole, and fused nitroimidazoles. Importantly, the toxicity of nitroimidazoles based on chemical scaffold types, medicinal chemistry of recently developed candidates, and preclinical and clinical profile of recently developed candidates is also highlighted and analyzed. Finally, a conclusion and perspectives are presented to produce readers our future vision of nitroimidazole scaffold modifications as future new drugs.

## 2. Activity Profile and Synthetic Pathways Developed to Construct Functionalized Nitroimidazole Derivatives

### 2.1. Functionalized 2-Nitroimidazole Scaffold

#### 2.1.1. Azomycin **1**

**Scheme 1:**
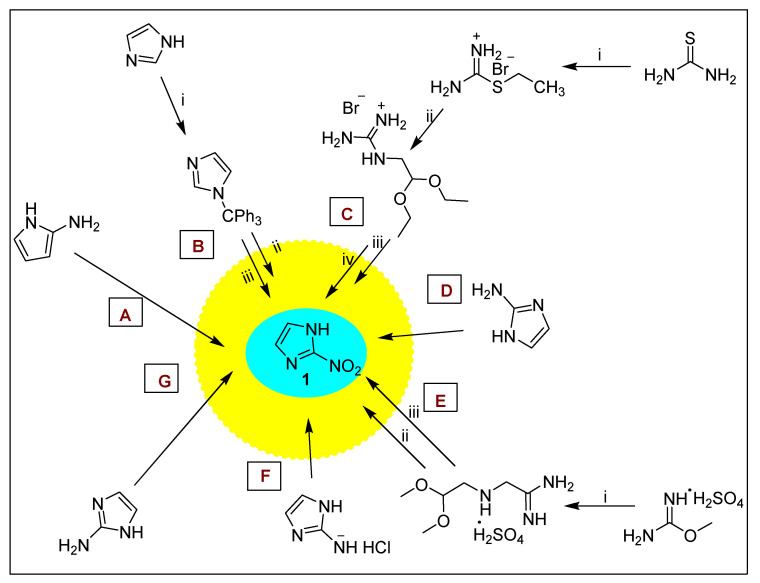
Approaches toward the synthesis of Azomycin **1.** Reagents and conditions: (**A**) NaNO_2_, HNO_3_, CuSO_4_, ~31%; (**B**) (**i**) Ph_3_CCl, Et_3_N, (**ii**) *n*-BuLi, THF, (**iii**) *n*-PrONO_2_; (**C**) (**i**) Ethyl bromide (**ii**) *β*-aminoacetaldehyde dimethyl acetal (**iii**) Conc. HCl, 65 °C; (**iv**) NaNO_2_, HCl, 36% (overall yield); (**D**) NaNO_2_, potters clay, MW, 100 °C, 82%; (**E**) (**i**) Conc. H_2_SO_4_, 50 °C; (**ii**) 2 h, 100 °C; (**iii**) H_2_SO_4_, NaNO_2_, CuSO_4_, NH_3_ Gaseous, 68%; (**F**) NaNO_2_, HBF_4_ followed by addition of copper powder and sodium nitrite ~49%; (**G**) Oxone, water, 40 °C; 83%.

Azomycin **1** represents the first nitroimidazole-based compound for therapeutic application and was first isolated from the bacteria’s culture, followed by attempts toward its synthesis (Scheme 1, routes A–G). In the initial days, the challenges posed for its synthesis led to the discovery of other regio-isomers (5-nitroimidazole and 4-nitroimidazole derivatives). As shown in Scheme 1, in 1965, Beaman et al. made a first successful attempt to synthesize 2-nitroimidazole from 2-aminoimidazole via two-step reactions (i) diazotization followed by (ii) nitration (route A) [39]. In 1982, Cohen et al. developed another synthetic route starting from imidazole, where imidazole was first converted to *N*-trityl imidazole. The *N*-trityl imidazole was then treated with *n*-butyl lithium to obtain 2-lithio intermediate, which upon treatment with *n*-propyl nitrate followed by acid hydrolysis, furnished 2-nitroimidazole **1** (route B) [26].

The promising profile of Azomycin **1** has continuously built interest among researchers, and therefore, several new routes were explored later. In 2001, Qing et al. constructed the 2-nitroimidazole ring using *β*-aminoacetaldehyde di-methyl acetal as the starting material. The *β*-aminoacetaldehyde di-methyl acetal was treated with *S*-ethylisothiourea to obtain *N*-(2,2-dimethoxyethyl)guanidine sulfate, which on treatment with conc. HCl, produced 2-aminoimidazole. The aminoimidazole underwent diazotization followed by nitration to produce Azomycin **1** (route C) [40]. In 2011, Phukan et al. also developed a solvent-free process for its synthesis from 2-nitroimidazole **1**, where potter’s clay and sodium nitrite under microwave conditions were used (route D) [41]. Wilde et al., in 2014, also developed a method where the 2-aminoimidazole ring was first constructed using amino acetaldehyde dimethyl acetal and *O*-methylisourea sulfate as starting materials, which then underwent diazotization, nitration, and finally furnished Azomycin **1** (route E) [42].

In 2014, Hui et al. also described the synthesis of Azomycin **1** from 2-aminoimidazole hydrochloride via diazotization (using 40% fluoroboric acid and sodium nitrite) followed by nitration (sodium nitrite and copper powder) (route F) [43]. In 2014, Zhao et al. developed a green and facile approach for the synthesis of 2-nitroimidazole **1** by treating 2-aminoimidazole with oxone as an oxidant in the presence of water (route G) [44].

#### 2.1.2. Benznidazole **2** and Its Derivatives

**Figure 5:**
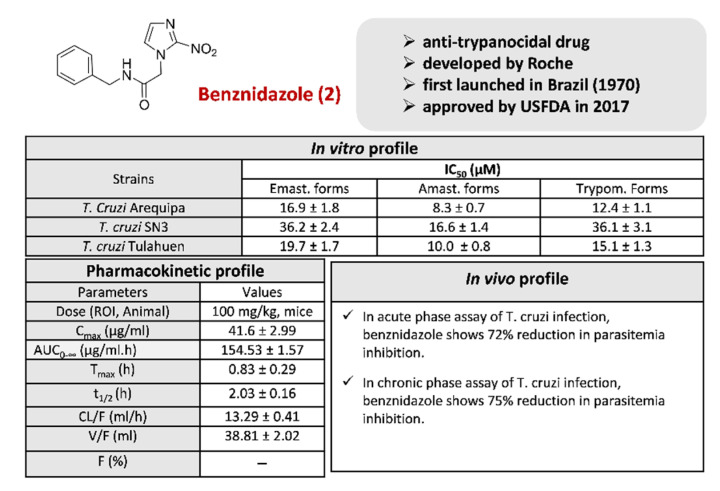
Activity profile of Benznidazole **2**.

Benznidazole **2**, belongs to the 2-nitroimidazole-based drug used in the treatment of Chagas disease, a disease caused by the protozoan parasite, *Trypanosoma cruzi* (Figure 5). Benznidazole **2** was developed and commercialized by Roche and was first launched in Brazil (in 1970) [45]. Recently, in 2017, the FDA has also given an accelerated approval for pediatric use [46]. Benznidazole **2** has shown good in vitro results against several strains of Trypanosoma such as *Trypanosoma cruzi Arequipa*, *Trypanosoma cruzi SN3*, *Trypanosoma cruzi Tulahuen* in all the three different stages, i.e., extracellular epimastigote (Emast.), intracellular amastigote (Amast.) and trypomastigote (Trypom.). Moreover, Benznidazole **2** has shown good in vivo activity against both acute and chronic mice models of infection [47,48]. The pharmacokinetic parameters studied by Perin et al. suggest that Benznidazole **2** has rapid and low absorption after oral administration (100 mg/kg, in mice). In clinical studies, it has been observed that benznidazole **2** has a better parasitological cure rate in patients with acute infection. Benznidazole **2** is an orally administered drug (with a human dose of 5–10 mg/kg/day for 30–60 days) and has been in use for the past ~50 years [49].

**Scheme 2:**
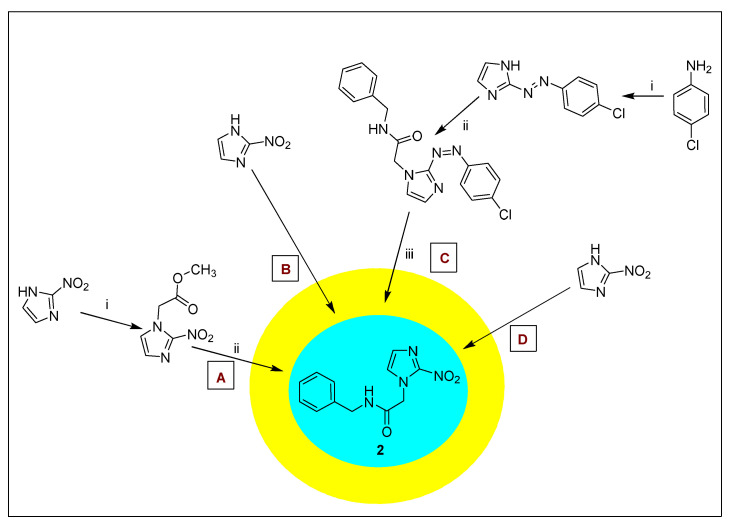
Approaches toward the synthesis of Benznidazole **2**. Reagents and conditions: (**A**) (**i**) methyl chloroacetate, sodium methoxide, DMF, 150 °C; (**ii**) benzylamine, 100 °C; (**B**) *N*-benzyl-2-hydroxyacetamide, MW; (**C**) (**i**) NaNO_2_, HCl, <5 °C followed by addition of imidazole, (**ii**) *N*-benzyl-2-chloroacetamide, disodium carbonate, H_2_O, rt, >−5 °C, (**iii**) NaH, DMF, 60 min, 0–25 °C; (**D**) ethylbromoacetate, K_2_CO_3_, EtOH, 70 °C, 110 min followed by addition of benzylamine at 50 °C for 16 h, 86%.

As shown in Scheme 2 (routes A–D), the effort toward the synthesis of Benznidazole **2** has been made by many researchers. However, its first synthesis was performed by Gamaliel et al. from Hoffmann-La Roche AG in 1969, wherein 2-nitroimidazole was condensed with methyl chloroacetate in the presence of a base to obtain methyl-2-nitro-1-imidazolylacetate, which on subsequent treatment with benzylamine, produced Benznidazole **2** (Scheme 2, route A) [50]. In 2015, Handal et al. from Ministerio de Educacion developed another eco-friendly, economical method for the synthesis of Benznidazole **2** (Scheme 2, route B), where *N*-benzyl-2-hydroxyacetamide was treated with 2-nitro-1*H*-imidazole under microwave conditions [51]. In 2016, Donadio et al. from Consejo Nacional de Investigaciones patented a three-step route for the synthesis of Benznidazole **2**. The synthesis involved (i) diazotization of 4-chloroaniline and coupling with imidazole; (ii) 2-(4-chlorophenyl)azoimidazole underwent *N*-alkylation with *N*-benzy-2-chlorolacetamide and iii) hydrogenation of the synthesized intermediate produced Benznidazole **2** (Scheme 2, route C) [52]. In 2017, Lynsey et al. developed a one-pot method where 2-nitroimidazole was first treated with haloacetate ester, and then the addition of benzylamine produced Benznidazole **2** (Scheme 2, route D) [53].

**Scheme 3:**
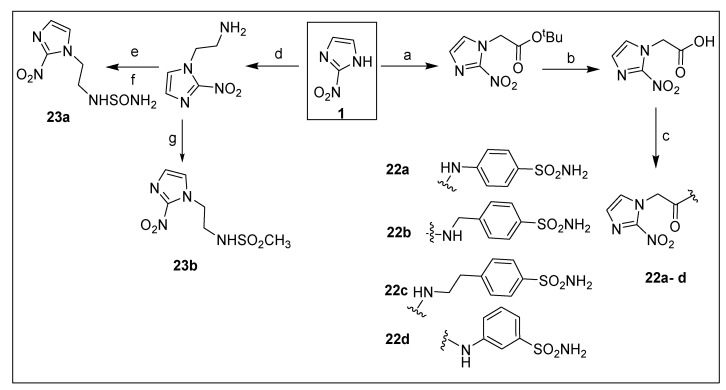
Approaches toward the synthesis of Benznidazole derivatives as hypoxia-targeting carbonic anhydrase IX inhibitors. Reagents and conditions: (**a**) *tert*-butyl bromoacetate, potassium carbonate, ACN, rt, overnight, 71%; (**b**) TFA:Water:Thioanisole (95:2.5:2.5), rt, overnight, 100%; (**c**) Amines, DMAP, EDC, DMA, rt, 2 days, 68–89%; (**d**) potassium carbonate, *tert*-butyl-2-bromoethyl carbamate, 40%; (**e**) TFA-DCM (8:2), rt, 2 h; (**f**) triethylamine, chlorosulfonylisocyante, *tert*-butanol, DCM, rt, overnight, 70%; (**g**) methane sulfonyl chloride, triethylamine, DCM, 45%.

Winum et al. synthesized sulfonamides and sulfamides containing Benznidazole derivatives (**22** and **23**) and evaluated them as radio/chemosensitizing agents that target the tumor-associated carbonic anhydrase (CA) isoforms I, II, IX and XII. Most of the compounds have shown nanomolar activity against CA IX and XII (Scheme 3) [54].

#### 2.1.3. Misonidazole **3** and Its Derivatives

**Figure 6:**
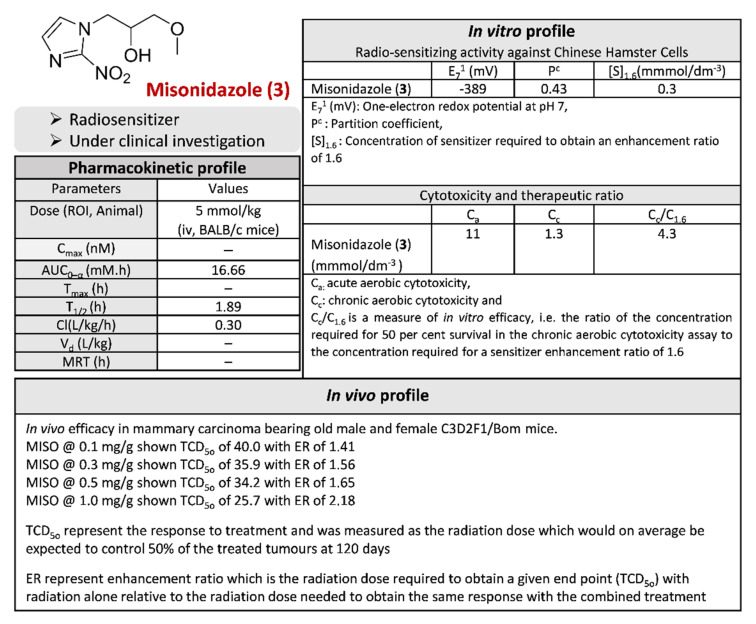
Activity profile of Misonidazole **3** [57,58,59].

Misonidazole (MISO, **3**) is another 2-nitroimidazole derivative discovered as a radiosensitizer to sensitize resistant hypoxic tumor cells toward treatment [55]. Misonidazole **3** has shown good in vitro, in vivo radio-sensitizers properties and pharmacokinetics properties (Figure 6) [55]. In the clinical study, none of the clinical trials demonstrated significant results, except for one where the small degree of radiosensitization was observed at a dose lower than the clinically recommended dose [56].

Nitroimidazoles have been known to undergo reduction to the RNO_2_ radical under hypoxia conditions and to bind to tissue macromolecules and exerts their effects. Considering this property, F-18 fluoromisonidazole (FMISO) was also developed as a diagnostic marker for hypoxia detection in cancer cells. It is extensively studied for in vivo imaging because of its high tissue penetration properties. F-18 FMISO has been evaluated in clinical trials (NCT00038038) against several tumors, including head and neck cancer.

**Scheme 4:**
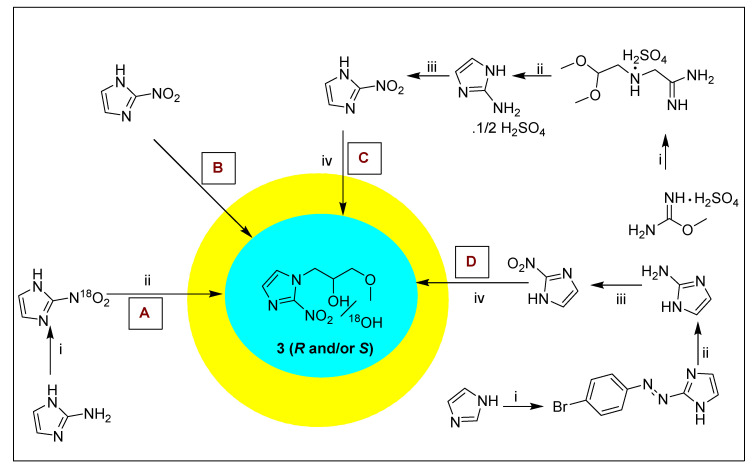
Approaches toward the synthesis of Misonidazole **3.** Reagents and conditions: (**A**) (**i**) NaN^18^O_2_, H_2_SO_4_, CuSO_4_, (**ii**) K_2_CO_3_, 1-2-epoxy-3-methoxypropane, 62%; (**B**) R/S-alkyl glycidyl ether, sodium carbonate, EtOH, reflux, 81/82%; (**C**) (**i**) 2-aminoacetaldehyde dimethyl acetal, 50 °C followed by addition of conc. H_2_SO_4_, (**ii**) 2 h, 100 °C, (**iii**) H_2_SO_4_, NaNO_2_, CuSO_4_, NH_3_ Gaseous, (**iv**) *R*/*S*-2-(methoxy methyl) oxirane, Na_2_CO_3_, EtOH, 5 h, 60 °C, 28/43%; (**D**) (i) 4-bromo aniline, NaNO_2_, HCl, <5 °C, (**ii**) H_2_, Raney nickel, (**iii**) H_2_SO_4_, NaNO_2_, CuSO_4_, NH_3_ gaseous, (iv) 1,2-epoxy-3-methoxypropane, K_2_CO_3_, EtOH, reflux, 5 h, 60 °C.

The first synthesis of Misonidazole **3** was developed by Yang et al. in 1989, starting from 2-aminoimidazole, which on reaction with sodium nitrite and sulfuric acid, produced 2-nitroimidazole. The 2-nitroimidazole upon further reaction with 1,2-epoxy-3-methoxypropane produced [^18^O]-Misonidazole **3** (Scheme 4, route A) [60]. Jin et al. in 2004 synthesized both (*R*)- and (*S*)-Misonidazole **3** by treating 2-nitroimidazole with (*R*)- and (*S*)-epichlorohydrins, respectively (Scheme 4, route B) [61]. In 2014, Wilde et al. synthesized optically pure Misonidazole **3**, wherein the reaction of 2-aminoacetaldehyde dimethylacetal with *O*-methylisourea sulfate led to the formation of the 2-aminoimidazole hemisulfate, which on cyclization and diazotization followed by nitration, produced 2-nitroimidazole [42]. Then, 2-nitroimidazole further underwent a nucleophilic opening reaction with *R* and *S*-2-(methoxy methyl)oxirane and finally led to the optically pure Misonidazole **3** (Scheme 4, route C). In 2016, Donadio et al. from Consejo Nacional de Investigaciones developed the new patented route where imidazole was first treated with *p*-bromobenzenediazonium chloride and generated an azo compound, which upon reduction followed by nitration, produced 2- nitroimidazole. The 2-nitroimidazole on condensation with 1,2 epoxy, 3-methoxy propane produced Misonidazole **3** (Scheme 4, route D) [52].

**Scheme 5:**
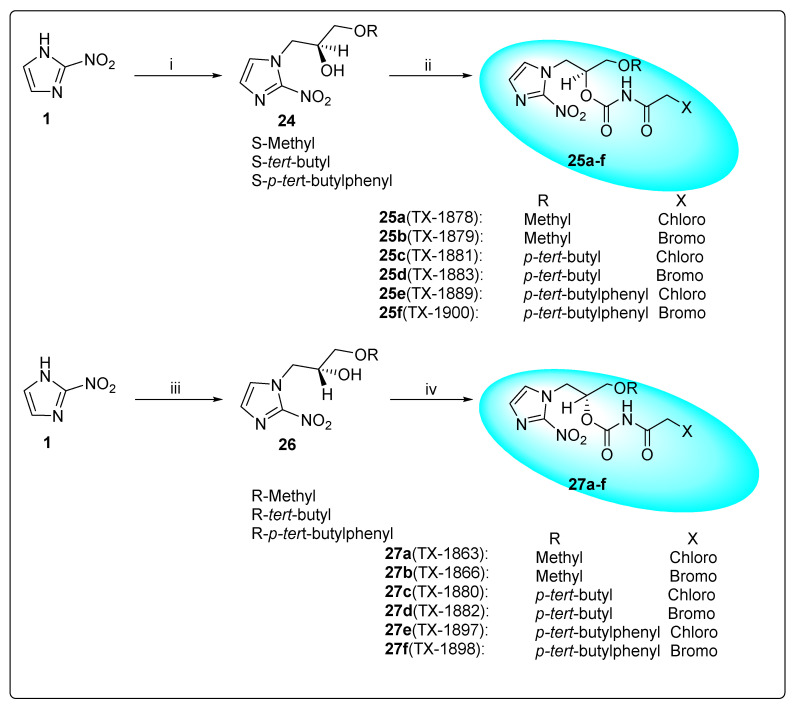
Synthesis of Misonidazole derivatives. Reagents and conditions: (**i**) (*S*)-alkyl glycidyl ether/anhydrous EtOH, Na_2_CO_3_, reflux; (**ii**) haloacetylisocyanate/anhydrous CH_2_Cl_2_, rt, 72–98%; (**iii**) (*R*)-alkyl glycidyl ether/anhydrous EtOH, Na_2_CO_3_, reflux; (**iv**) haloacetylisocyanate/ anhydrous CH_2_Cl_2_, rt, 72–98%.

Jin et al. in 2004 designed the Misonidazole-based derivatives (TX) as dual function anti-angiogenic and hypoxia cell radio-sensitizers, wherein 2-nitroimidazole moiety is for hypoxic cell radiosensitization, and the second part contains a haloacetyl carbamoyl group as an anti-angiogenesis pharmacophore [61]. Twelve compounds were synthesized using epichlorohydrin as a chiral starting material wherein the single stereocenter connects a haloacetylcarbamoyl, 2-nitroimidazolyl, and diverse alkyl or aryl groups of varying steric dimensions (Scheme 5 and Table 1). Then, the synthesized compounds were tested for protease inhibition, in vitro anti-angiogenic activity using RLE cell proliferation assay and a chick embryo CAM assay, as well as for hypoxic cell radiosensitizers. The evaluation in the latter assay revealed that *R*-enantiomer having the bulky 4-*tert*-butylphenyl group displayed higher anti-angiogenic activity, while the enantiomers bearing the less bulky methyl and *tert*-butyl groups did not show any differences in activity. Among all, compound **27f** (**TX-1898**) has been identified as a potent anti-angiogenic hypoxic cell radiosensitizer that can be explored for further development.

#### 2.1.4. Pimonidazole **4** and Its Derivatives

**Figure 7:**
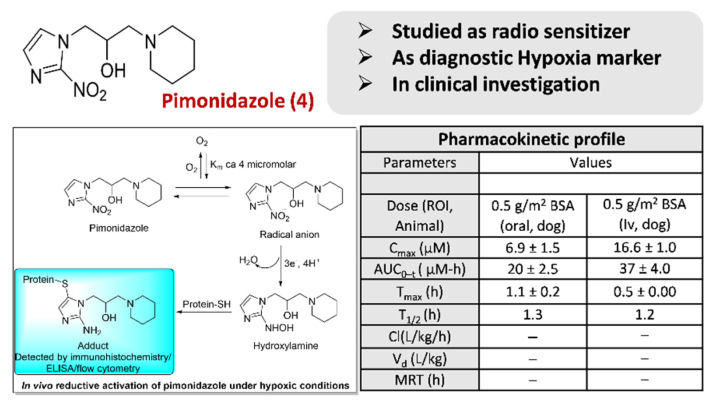
Activity profile of Pimonidazole **4** [34,63].

Pimonidazole **4** was developed as a diagnostic marker for the identification of tumor hypoxia condition. It becomes covalently attached to thiol-containing proteins in hypoxic cells after reductive activation in an oxygen-dependent manner, and the adduct formed can be detected using immunohistochemistry, ELISA, and flow cytometry [46]. Pimonidazole **4** has shown robust and effective diagnostic hypoxia markers and is currently under investigation to diagnose cancers such as prostate cancer and head and neck cancer. Pimonidazole **4** has also shown better oral bioavailability than other hypoxia markers (Figure 7) [34,62]. It has also been reported that Pimonidazole **4** sensitizes the tumor cells toward radiation. The radiosensitizing property is because of the accumulation of resulting complexes in hypoxic tumors, thereby depleting radio-protective thiol compounds [63].

**Scheme 6:**
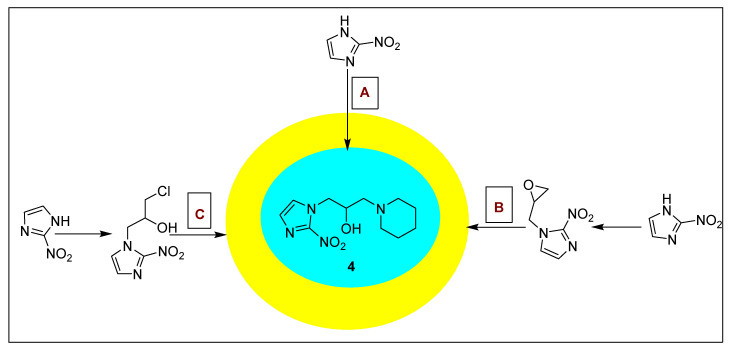
Synthesis of Pimonidazole **4**. Reagent and conditions: (**A**) 3-(1-piperidino) propylene oxide, ethanol, reflux (yield not reported); (**B**) (**i**) 1,3-dichloroprpan-2-ol, potassium carbonate, DMF, (**ii**) piperidine, EtOH, reflux (yield not reported); (**C**) (**i**) epichlorohydrin, K_2_CO_3_, 60 °C, (**ii**) piperidine, EtOH, reflux (yield not reported).

The synthesis of Pimonidazole **4** has been reported by three routes (Scheme 6, route A–C). In the first route, Pimonidazole **4** was synthesized by condensation of 2-nitroimidazole with 3-(1-piperidino)propylene oxide in refluxing ethanol. In route B, nitroimidazole reacted with epichlorohydrin followed by condensation with piperidine in refluxing ethanol. In route C, Pimonidazole **4** was synthesized by reacting 1,3-dichloropropane-2-ol with 2-nitroimidazole followed by condensation of a generated intermediate (1-(3-chloro 2 hydroxypropyl)-2-nitroimidazole) with piperidine in ethanol [64].

**Scheme 7:**
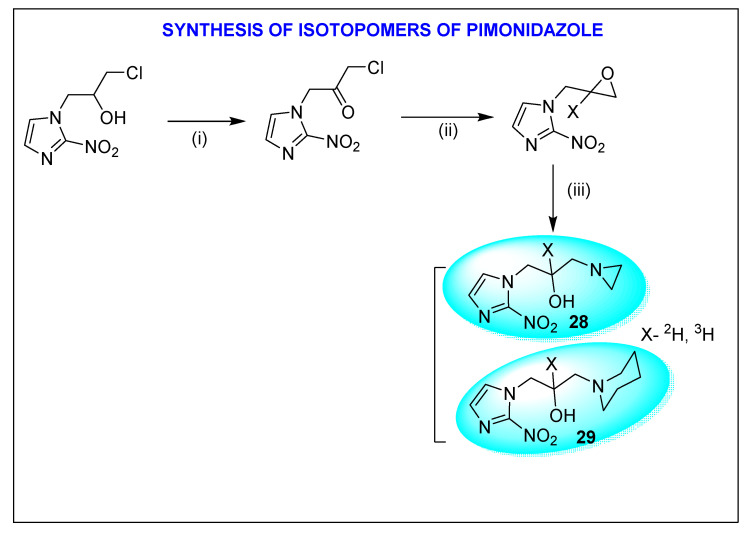
Synthesis of isotopomers of Pimonidazole **28** and **29**. Reagent and conditions: (**i**) Cr_2_O_4_, acidic acetone, 46%; (**ii**) labelled NaBH_4_, 76% (X-^2^H) and 95% (X-^3^H); (**iii**) aq. base-EtOH, followed by aziridine/piperidine, 94% (**28**, aziridine, X-^2^H), 99% (**29**, piperidine, X-^2^H).

Threadgill et al. (1990) synthesized labeled analogs of Pimonidazole **4** and its other related analogs RSU 1069 (having azridine instead of piperdine) to unravel its pharmacological profile (Scheme 7). In this paper, the authors have synthesized ^2^H and ^3^H isotopomers of RSU 1069 **28** and Ro 03-8799 **29** (Pimonidazole). The compounds were synthesized by reduction of l-(3-chloro-2-oxopropyl)-2-nitroimidazole with labeled sodium borohydride, followed by ring closure of the chlorohydrins and treatment of the resulting epoxides with aziridine or piperidine. Both compounds have shown the specific activities of 200 mCi mmol^−1^ and radiochemical yields of 86%. Both aziridinyl- and piperidinyl-containing compounds represent the second generation radiosensitized and have better activity and safety than Misonidazole. The aziridine-containing compounds have even shown better selective toxicity toward the hypoxic cells due to their bifunctional electrophilic behavior under hypoxia conditions versus the standard compound Misonidazole [65].

#### 2.1.5. Evofosfamide **5**

**Figure 8:**
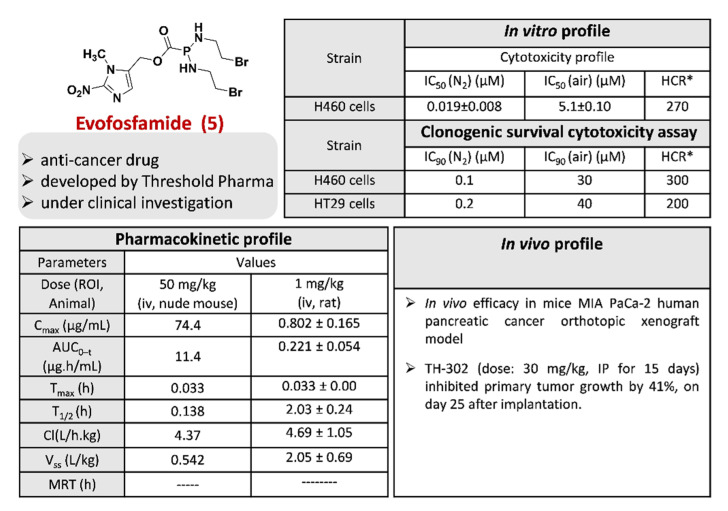
Activity profile of Evofosfamide **5** [67,68,69,70]. *: Hypoxia cytotoxicity ratio (HCR) was determined by the differential cytotoxicity under normoxic and hypoxic conditions: HCR=IC_50_(air)/IC_50_(N_2_).

Evofosfamide **5** (formerly known as TH-302) is an investigational hypoxia-activated drug developed by Threshold Pharmaceuticals in 2012 and is in clinical development for cancer treatment (Figure 8) [66,67,68,69,70]. Evofosfamide was designed by coupling 2-nitroimidazole and brominated derivative of isophosphoramide mustard. The prodrug is activated under hypoxic conditions typical of solid tumor cancer cells [70]. Evofosfamide **5** undergoes a one-electron reduction by ubiquitous cellular reductases, which under low oxygen conditions, releases a DNA-crosslinking anti-cancer agent, phosphoramidate. Evofosfamide **5** was selectively potent under hypoxia conditions and stable toward the hepatocytes. In an in vivo efficacy study, evofosfamide was found to be active in a pancreatic (MIA PaCa-2) cancer orthotopic xenograft model as a monotherapy. Moreover, its combination with gemcitabine also showed dramatic efficacy [14]. It is being studied in clinical trials as a therapeutic agent [66].

**Scheme 8:**
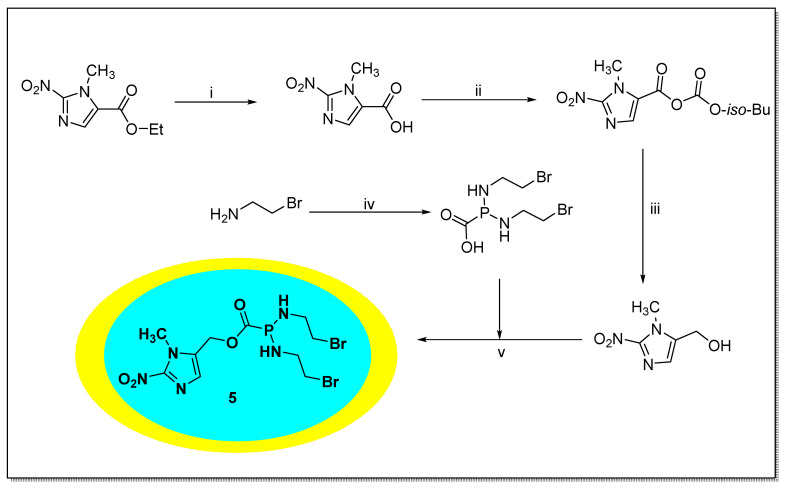
Duan et al. method of synthesis of Evofosfamide **5.** Reagents and conditions: (**i**) NaOH, (**ii**) *iso*-BuOCOCl, Et_3_N, (**iii**) NaBH_4_, NaOH, (**iv**) POCl_3_, POBr_3_, Et_3_N, (**v**) PPh_3_, DIAD, (overall yield ~48%).

Duan et al. in 2008 developed a method for the synthesis of Evofosfamide **5** (Scheme 8) [13]. In this method, synthesis of Evofosfamide **5** was carried out by hydrolysis of ethyl-1-methyl-2-nitroimidazole-5-carboxylate followed by a reaction with isobutylchlorocarbonate, resulting in the formation of anhydride (Scheme 8). The reduction of anhydride intermediate produced 1-methyl-2-nitroimidazole-5-yl methanol, which underwent Mitsunobu reaction to produce the desired product.

**Scheme 9:**
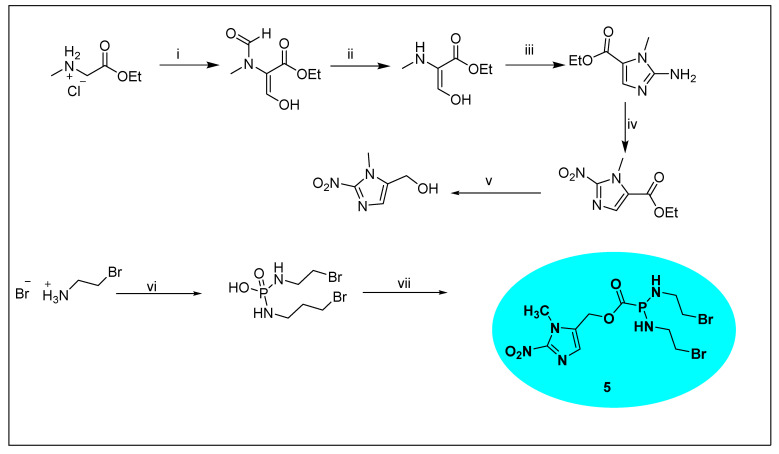
Connor et al. synthesis of Evofosfamide **5**. Reagents and conditions: (**i**) EtOCHO, THF, NaH, 3 h, (**ii**) EtOH, conc. HCl, 90 °C, 2 h, (**iii**) EtOH, H2O, NH2CN, pH 3, 100 °C, 1.5 h, 48–54% (over three steps), (**iv**) AcOH, NaNO2 (aq), rt, 4 h, 72%, (**v**) NaBH4, THF, MeOH, 0 °C → rt, 2 h, 66%, (**vi**) POCl3, Et3N, CH2Cl2, −10 °C (yield not reported), (**vii**) PPh3, DIAD, THF, 0 °C → rt, 3 h, 62%.

Connor et al. (2015) developed another route for the synthesis of Evofosfamide **5** (Scheme 9). This method involves the use of sarcosine ethyl ester hydrochloride as a starting material, which was converted into *N*-formylated enolate followed by deformylation, treatment with cyanamide, diazotization, and a reduction produced the main intermediate, 2-nitroimidazole alcohol. The bromoisophosphoramide mustard intermediate was synthesized from the corresponding 2-bromoethylamine hydrobromide salt, which on subsequent treatment with the 2-nitroimidazole alcohol under Mitsunobu conditions, produced Evofosfamide **5** (TH-302) [71].

#### 2.1.6. Miscellaneous 2-Nitroimidazole Derivatives

**Scheme 10:**
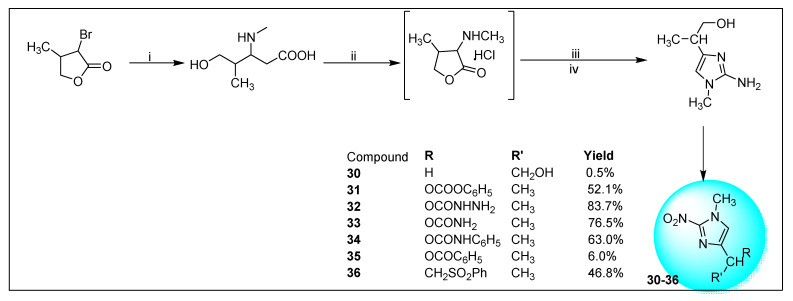
Synthesis of 4-substituted 2-nitromimdazole derivatives. Reagents and conditions: (**i**) CH_3_NH_2_, (**ii**) HCl/EtOH, (**iii**) HgNa/EtOH, (**iv**) NH2CN (yields not reported), (**v**) HBF_4_, NaNO_2_, Cu, NaNO_2_ (yields given above).

Cavalleri et al. performed the modification of 2-nitromimdazole and evaluated them as antitrichomonas agents. In this study, the authors synthesized several analogs (**30**–**36**), and among them, one molecule **30** has shown better potency and lesser toxicity as compared to the standard molecule (Scheme 10 and Table 2) [72].

**Scheme 11:**
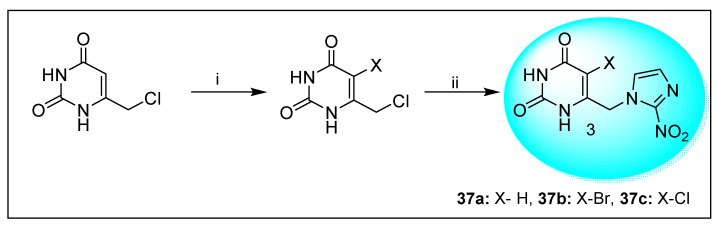
Synthesis of nitro and aminoimidazolyluracil conjugates. Reagents and conditions: (**i**) NCS or NBS, AcOH, 60 °C, **37a** (67%), **37b** (77%); (**ii**) 1-potassio-2-nitroimidazole, DMF, rt, N2, 24 h; **37a** (61%), **37b** (75%), **37c** (35%).

Cole et al. (2003) developed the 2-nitroimidazolylmethyluracils **37a**–**c** and 2-aminoimidazolylmethyluracils (Scheme 11) as inhibitors of thymidine phosphorylase (TP). In this study, the authors identified 2-aminoimidazolylmethyluracils as potent TP inhibitors with IC_50_ values of ~20 nM as compared with 2-nitroimidazolylmethyluracil (as bio-reductively activated) prodrugs (**37b**/**37c**), which were 1000-fold less active with IC_50_ values ranging from 22–24 µM [73].

**Scheme 12:**
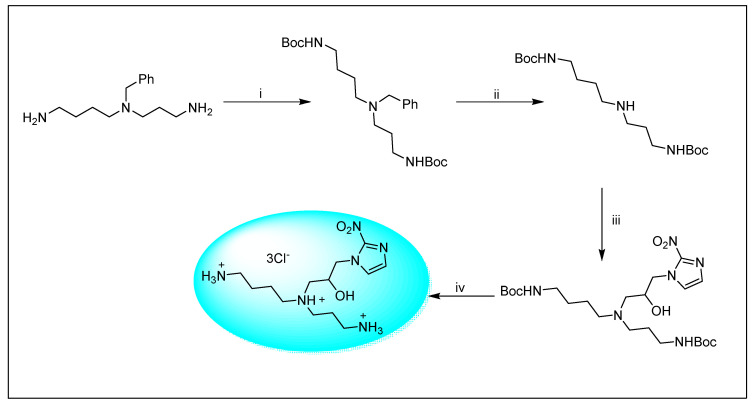
Synthesis of nitroimidazole-spermidine derivatives. Reagents and conditions: (**i**) (*t*-Boc)_2_O, Et_3_N, THF, rt, 85%; (**ii**) Pt/C, H_2_, MeOH, 98%; (**iii**) 1-(oxiranylmethyl)-2-nitroimidazole, EtOH, reflux, 65%; (**iv**) HCl gas in dioxane, THF 97%.

Papadopoulou et al. in 2004 synthesized nitroimidazole-spermidine derivatives as a cancer-targeted hypoxia-selective cytotoxin. In this, (*R*,*S*)-*N*4-[3-(2-nitro-1-imidazolyl)-2-hydroxypropyl]-spermidine trihydrochloride **38** was synthesized and evaluated as a hypoxia-selective cytotoxin and radiosensitizer in V79 cells (Scheme 12) [74].

**Scheme 13:**
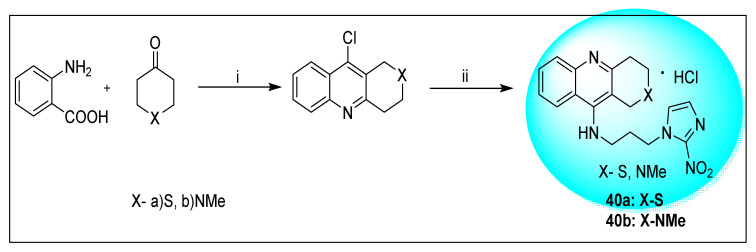
Synthesis of 2-nitroimidazole-tethered tricyclic quinolones. Reagents and conditions: (**i**) POCl3, reflux, **40a** (55%), **45b** (47%); (**ii**) phenol, NaI, 3-(2-nitro-1-imidazolyl)propylamine, 130 °C, 15 min, followed by addition of HCl gas in dioxane, **40a** (16–27%), **40b** (7–22%).

Papadopoulou in 2004 also synthesized novel nitroimidazole-based bioreductive compounds, 10-[3-(2-nitroimidazolyl)-propylamino]-3,4-dihydro-1*H*-thiopyrano [4,3-*b*]quinoline hydrochloride (**39a**) and 10-[3-(2-nitroimidazolyl)propylamino]-2-methyl-1,2,3,4-tetrahydro-benzo[*b*]-1,6-naphthyridine hydrochloride (**39b**) and evaluated in V79 cells hypoxia-selective cytotoxins and radiosensitizers that target DNA through weak intercalation. Both compounds were relatively good radiosensitizers (C_1.6_ values of 40.0 ± 0.8 and 59.0 ± 0.4 μM for **39a** and **39b**, respectively) but less potent cytotoxins (Scheme 13) [75].

**Scheme 14:**
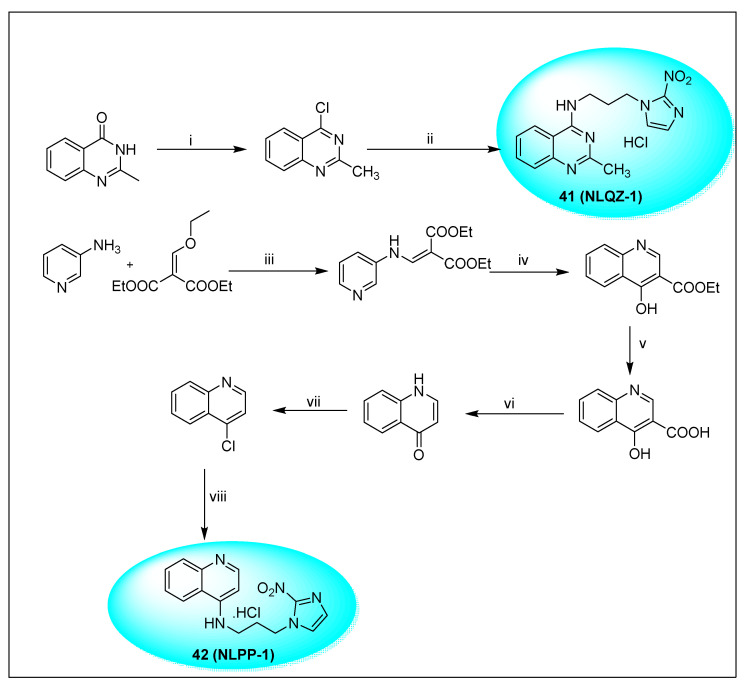
Synthesis of nitroimidazole-based quinazoline or naphthyridine moiety. Reagents and conditions: (**i**) POCl_3_, 2.5 h, 50%; (**ii**) 3-(2-mitro-1*H*-imidazol-1-yl)propan-1-amine, propanol, reflux, 12 h, 70%; (**iii**) dowtherm A, 150 °C; (**iv**) reflux, 250 °C, 100%; (**v**) NaOH, reflux, 78%; (**vi**) mineral oil, 320 °C, 95%; (**vii**) POCl_3_, reflux, 62%; (**viii**) 3-(2-mitro-1*H*-imidazol-1-yl)propan-1-amine, propanol, reflux, 12 h, 97 °C, 58%.

Papadopoulou (2009) synthesized nitroimidazole-based bio-reductive compounds having a quinazoline (**41**, NLQZ-1) or a naphthyridine moiety (**42**, NLPP-1, (Scheme 14). The synthesized compounds were evaluated against V79 and A549 cells for their cytotoxicity, radiosensitization and interaction with chemotherapeutic agents by using the clonogenic assay, and the results revealed that the hypoxic selectivity was slightly increased as in the case of **42** (NLPP-1), where it ranged from 12–19 in V79 and 15–26 in A549 cells. Both compounds have shown better radiosensitizer against hypoxic V79 cells at nontoxic concentrations, as shown in Table 3. Both compounds have shown a synergistic effect with cisplatin or melphalan in V79 cells under hypoxic pre-exposure conditions [76].

**Scheme 15:**
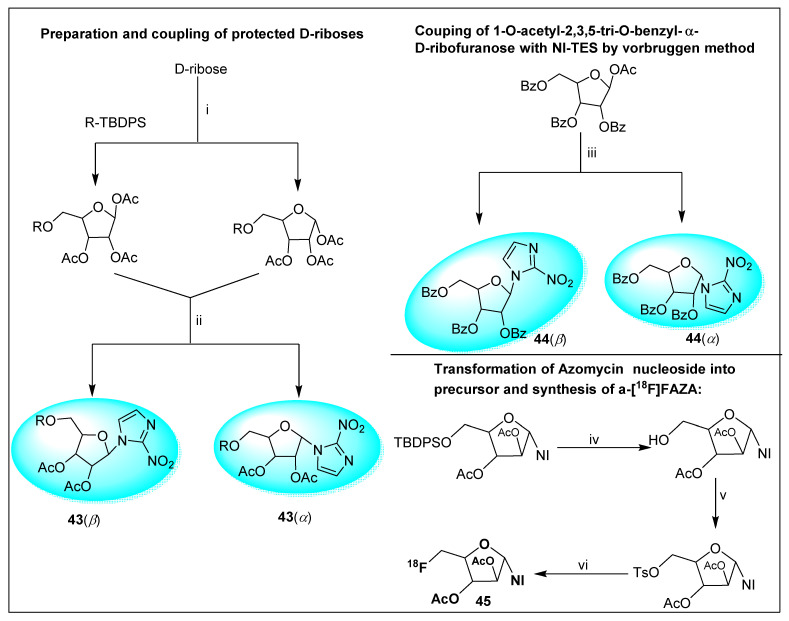
Synthesis of 2-nitroimidazole-based nucleosides. Reagents and conditions: (**i**) TBDPSCl/Pyridine, −20 °C-rt, Ac_2_O, 50 °C, 28% and 33%; (**ii**) NI-TS/TfOTES, 60%; (**iii**) NI-TS/TfOTES, 31% and 42%; (**iv**) KF/PhCOOH, ACN, reflux; (**v**) TsCl/Pyridine, 94%; (**vi**) K^18^/Kryptofix, DMSO, heat, NaOH (yield not reported).

Schweifer et al. (2011) designed the nucleosides derived from 2-nitroimidazole and D-arabinose, D-ribose, and D-galactose and evaluated their potential as tracers to image hypoxia. 2-Nitroimidazole was first silylated with hexaethyldisilazane and then coupled with 1-*O*-acetyl derivatives of D-arabinose, D-ribose, and D-galactose under Vorbruggen conditions. When the C-5 hydroxyl group of D-arabinose and D-ribose were silylated with *tert*-butyldiphenylsilyl chloride followed by acetylated in a one-pot reaction, mixtures of anomeric 1-*O*-acetyl derivatives were obtained. These were then coupled by using the Vorbruggen method followed by cascades to furnish the precursors for tracers to image hypoxia. [77] The representative example of nitroimidazole-ribose-based conjugate and arabinose-based hypoxia images is shown in Scheme 15.

**Scheme 16:**
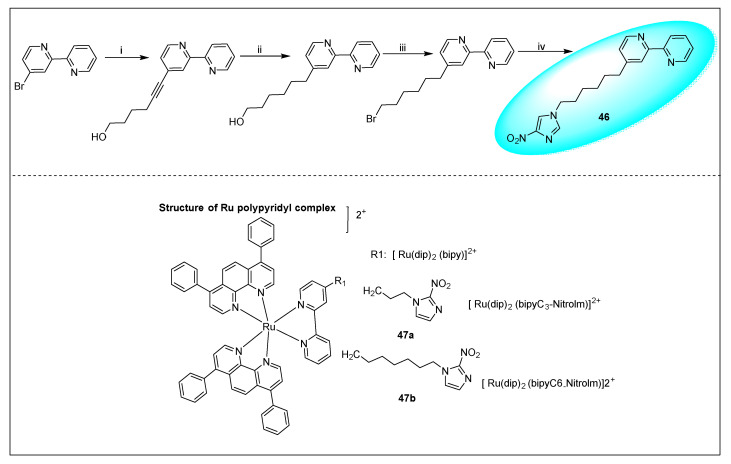
Synthesis of bipyridyl complexes. Reagents and conditions: (**i**) 5-hexyn-1-ol, Et_3_N, Pd(PPh_3_), DME, 120 °C, 2.5 h, 63%; (**ii**) H_2_, Pd/C, EtOH, rt, 4 days, 77%; (**iii**) CBr_4_, PPh_3_, DCM, rt, overnight, 60%; (**iv**) 2-nitroimidazole, K_2_CO_3_, DMF, 110 °C, overnight, 79%.

Mazuryk et al. (2017) designed the nitroimidazole-based derivatives of polypyridyl ruthenium complexes **47** as anticancer agents (Scheme 16). The detailed biological investigation was performed for the ruthenium polypyridyl complexes comprising two 4,7-diphenyl -1,10-phenanthroline ligands; one unmodified 2,2′-bipyridyl or modified with 2-nitroimidazole moiety attached by shorter or longer linkers induced cell death. They evaluated the cytotoxicity and proliferation assays of Ru polypyridyl complexes **47** that reveal toxic potential of these compounds against human pancreas carcinoma PANC-1 cell line versus normal human keratinocytes HaCaT with IC_50_ values of 3-5μM. Then, the authors revealed the mechanism of Ru complexes toward their anti-cancer potential. The Ru complexes accumulate in the mitochondria and inhibit DNA synthesis, arresting the cell cycle in S-phase [78].

### 2.2. Functionalized 4-Nitroimidazole Scaffold

#### 2.2.1. Azathioprine **6** and Its Derivatives

**Figure 9:**
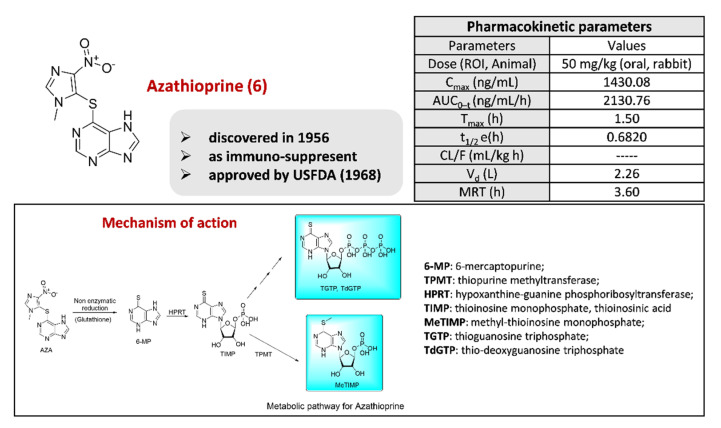
Activity profile of Azathioprine **6** [85].

Azathioprine **6** (AZA, Figure 9) is a prodrug of 6-mercaptopurine (named *BW 57-322*), first synthesized in 1956 to produce a derivative of 6-mercaptopurine in a metabolically active but masked form with a better therapeutic index [79]. It is used in rheumatoid arthritis, granulomatosis with polyangiitis, Crohn’s disease, ulcerative colitis, and in kidney transplants to prevent rejection. It is taken either by mouth or intravenously with good oral bioavailability. It is approved by the USFDA for use in kidney transplantation and rheumatoid arthritis and is on the WHO’s list of essential medicines [80,81,82,83,84]. Azathioprine **6** is sold under the brand name Imuran. Glutathione and similar compounds of the intestinal wall, liver and red blood cells mediate the reductive cleavage of the thioether (-*S*-) due to which Azathioprine **6** converts to 6-mercaptopurine (6-MP) [32,33,34]. Azathioprine **6** interferes with purine synthesis and disrupts the DNA and RNA, thus causing immunosuppression [35].

**Scheme 17:**
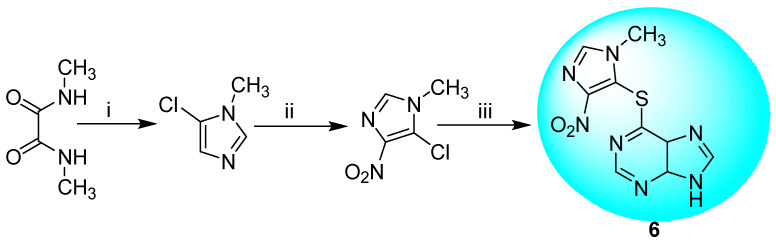
Synthesis of Azathioprine **6**. Reagents and conditions: (**i**) PCl_5_, (**ii**) KNO_3_, H_2_SO_4_, (**iii**) 6-mercaptopurine, DMSO, NaOAc (yields not reported).

Elion et al. (1965) developed a new patented route for synthesizing Azathioprine **6** (Scheme 17). In this, *N,N*-dimethyloxamide reacted with phosphorus pentachloride to produce 5-chloro-1-methylimidazole [86]. Then, 1-methylimidazole on treatment with potassium nitrate and concentrated sulfuric acid furnished 4-chloro-1-methyl-5-nitro-1*H*-imidazole**.** Then, the final step involves the condensation of 4-chloro-1-methyl-5-nitro-1*H*-imidazole with 6-mercaptopurine using dimethyl sulfoxide as solvent and sodium acetate as base.

**Scheme 18:**
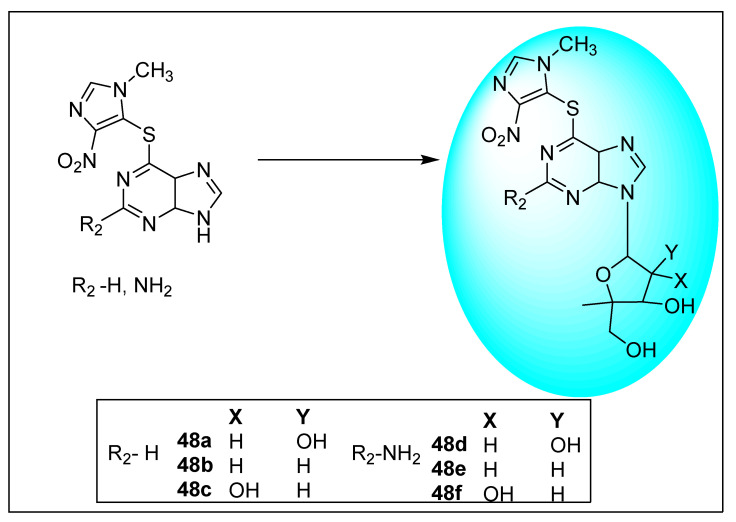
Synthesis of nucleosides of Azathioprine **6** and thiamiprine. Reagents and conditions: Purine nucleoside phosphorylase (EC 2.4.2.1) (yield not reported).

Krenitsky et al. (1989) synthesized nucleosides of Azathioprine **6** and Thiamiprine (**48a**–**f**) as antiarthritics agents. The reaction was catalyzed by purine nucleoside phosphorylase (EC 2.4.2.1). In this study, ribosides, deoxyribosides and arabinosides of azathiopurine and its 2-amino congener thiamiprine were synthesized, and their in vitro evaluation and cytotoxicity studies were also performed (Scheme 18) [87]. It was found that none of the congeners studied were superior to Azathioprine **6** itself.

**Scheme 19:**
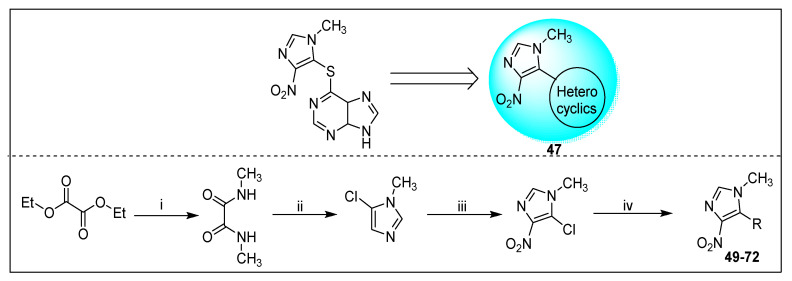
Synthesis of immunosuppressive Azathioprine analogs. Reagents and conditions: (**i**) MeNH_2_, (**ii**) PCl_5_, (**iii**) HNO_3_, H_2_SO_4_ (yield not reported), (**iv**) RSH, NaOH, H_2_O, 51–86%.

**Figure 10:**
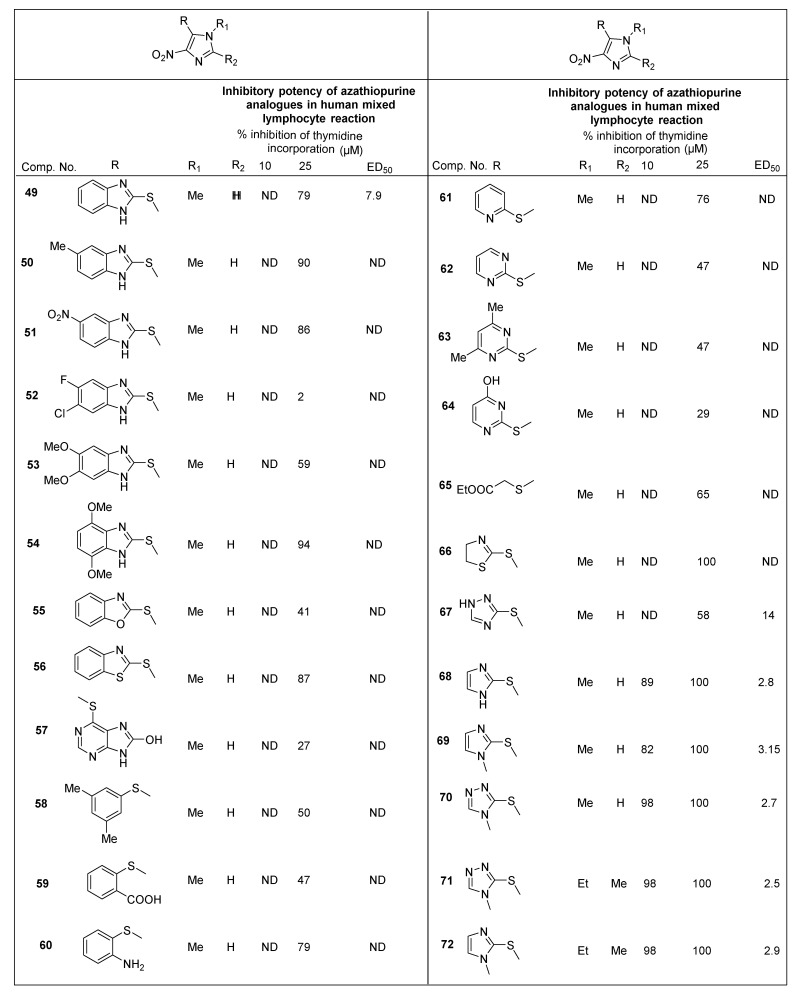
Inhibitory potency of Azathioprine analogs in a human-mixed lymphocyte reaction.

Crawford et al. (1996) designed the novel analogs of Azathioprine (**49**–**72**) lacking the 6-mercaptopurine substituent and evaluated their potential for retaining or having enhanced immunosuppressive effects (Scheme 19) [88]. Here, 24 analogs of Azathioprine **6** lacking a 6-mercaptopurine substituent were synthesized, and it was found that immunosuppressive effects are retained or even enhanced in these molecules. In this study, ten compounds have shown more potent activity than Azathioprine **6** in in vitro assays, and two analogs (**69** and **70**) have shown better potency in an in vivo assay (Figure 10) [88].

#### 2.2.2. Miscellaneous 4-Nitroimidazole Derivatives

**Scheme 20:**
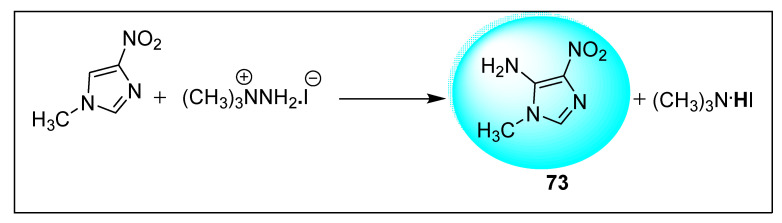
New method for the C-amination of 1-methyl-4-nitroimidazole. Reagents and conditions: *t*-BuOK(MeONa), DMSO, rt, 22 h, 56%.

Donskaya et al. (2002) developed a new method for the C-amination of 1-methyl-4-nitroimidazole. 1-Methyl-4-nitroimidazole was treated with (1-methylhydrazin-1-ium-1-ylidene)iodate in the presence of dry sodium methylate or potassium *tert*-butylate in DMSO to produce 5-amino-1-methyl- 4-nitroimidazole **73** in 56% yield (Scheme 20) [89].

**Scheme 21:**
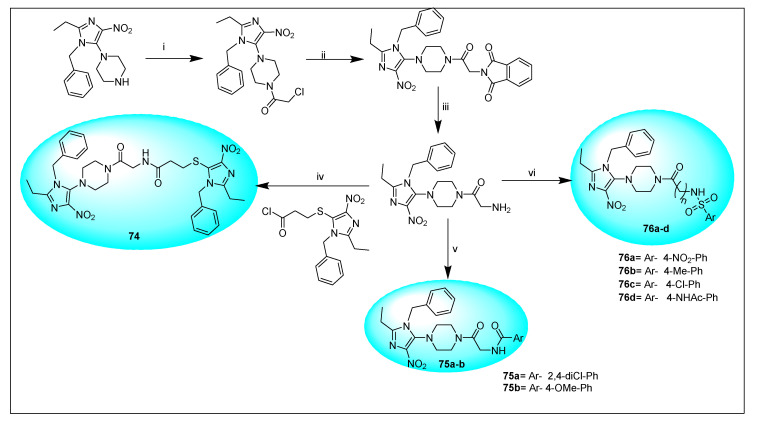
Synthetic pathway of [(4-nitro-1*H*-imidazol-5-yl)piperzin-1-yl)2-oxoethyl]aryl/alkyl-sulphonamide and benzamide derivatives as anti-HIV agents. Reagents and conditions: (**i**) 2-chloroacetyl chloride, Et_3_N, CH_2_Cl_2_, 23 °C, 3 h (yield not reported); (**ii**) potassium phthalimide, DMF, 120–130 °C, 24 h (yield not reported); (**iii**) NH_2_NH_2_.H_2_O, reflux, 4 h, 78%; (**iv**) Et_3_N, CH_2_Cl_2_, 23 °C, 16 h, 43%; (**v**) ArCOCl, Et_3_N, CH_2_Cl_2_, 23 °C, 16 h, 54% and 47%; (**vi**) RSO_2_Cl, Et_3_N, CH_2_Cl_2_, 23 °C, 16 h, 39–47%.

Al-Masoudi et al. developed new-generation 5-substituted piperazinyl-4- nitroimidazole derivatives (**74**–**76**) as anti-HIV agents (Scheme 21). [90] The analog **76c** was found to selectively inhibit HIV-1 replication.

**Scheme 22:**
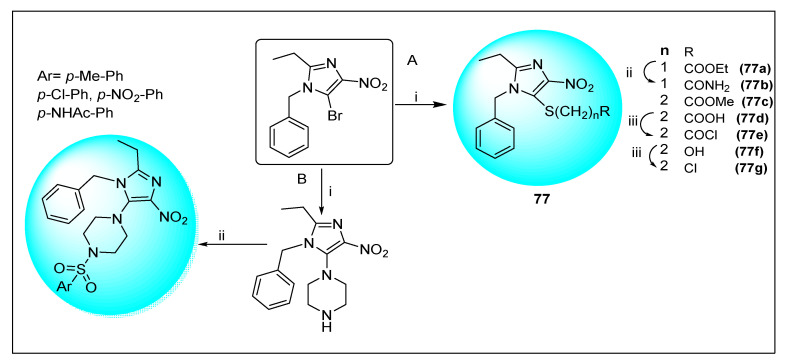
Synthesis of 5-alkylsulfanyl and 5-(4-arylsulfonyl)piperazinyl-4- nitroimidazoles as anti-HIV agents. Reagents and conditions: (**A**) (**i**) R(CH_2_)_n_SH, K_2_CO_3_, *i*-PrOH, 60–70 °C; 4 h, 60–83%; (**ii**) NH_3_/MeOH, 23 °C, 10 h, 90%; (**iii**) SOCl_2_, CHCl_3_, 23 °C, 18 h, 30%; (**B**) (**i**) piperazine, DMF, 70–80 °C, 6 h, 72%; (**ii**) ArSO_2_Cl, Et_3_N, CH_2_Cl_2_, 23 °C, 4 h, 43–53%.

Al-Soud et al. (2007) designed and synthesized the new 5-alkylsulfanyl and 5-(4-arylsulfonyl)piperazinyl-4-nitroimidazole-based derivatives **77** and **78**, respectively, as anti-HIV agents (Scheme 22). In this, the authors synthesized 15 new derivatives evaluated against HIV-1, and among them, two molecules **77e** and **77g** showed better EC_50_ inhibitions and a better safety index [91].

**Scheme 23:**
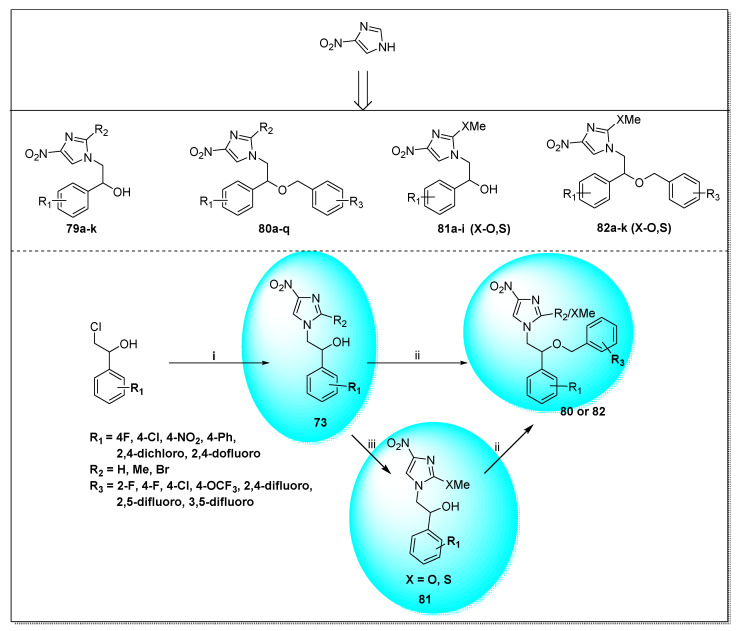
Synthesis of 4-nitroimidazole derivatives with antitubercular activity. Reagents and conditions: (**i**) 4-nitroimidazoles, TBAI, K_2_CO_3_, MeOH or EtOH, reflux, 12 h, 38–50%; (**ii**) benzyl bromides, NaH, TBAI, DMF, −78 °C to rt, 2.5 h, 90–94%; (**iii**) NaOMe or NaSMe, MeOH, rt, 12 h, 60–93%.

Lee et al. (2011) designed and synthesized econazole-based nitroimidazoles analogs (**79**–**82**) and evaluated them for antitubercular activity. The introduction of a nitro group at the 4-position of the imidazole of econazole abolished the anti-tubercular (Scheme 23) [92]. However, the introduction of an oxygen atom at the 2-position of nitroimidazoles helps to increase antitubercular activity.

**Scheme 24:**
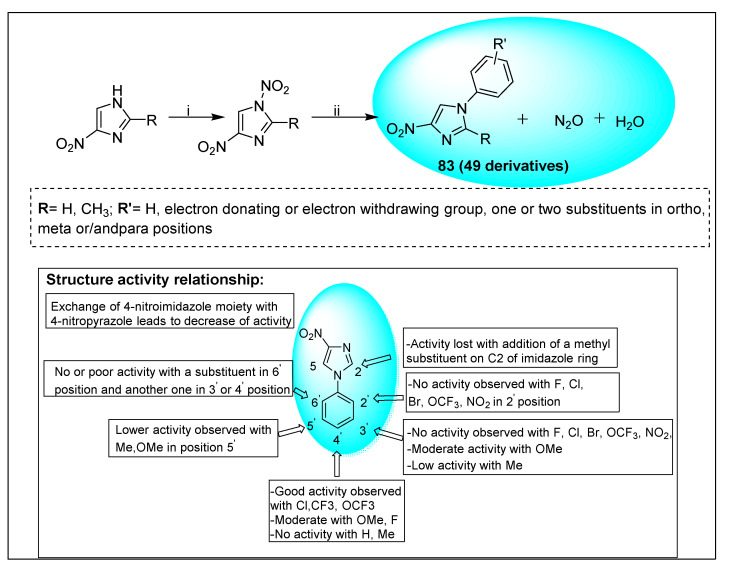
Synthesis of 4-nitroimidazole derivatives to treat human African trypanosomiasis. Reagents and conditions: (**i**) HNO_3_, Ac_2_O/AcOH; (**ii**) anilines, CH_3_OH-H_2_O, 40–90%.

Trunz et al. developed novel arylated analogs of 4-nitroimidazoles **83** and evaluated them for treating human African trypanosomiasis (Scheme 24) [93]. This series of forty-nine 1-aryl-4-nitro-1*H*-imidazoles was prepared, and extensive SAR was also studied, while two compounds, namely 4-nitro-1-{4-(trifluoromethoxy)phenyl}-1*H*-imidazole and 1-(3,4-dichlorophenyl)-4-nitro-1*H*-imidazole, were effective in mouse models of both acute African trypanosomiasis (oral dose of 25–50 mg/kg for 4 days) and chronic African trypanosomiasis (oral dose of 50–100 mg/kg for 5 days). Both compounds demonstrated potent and selective anti-trypanosomal activity, including the stringent model of second-stage human African trypanosomiasis, the chronic CNS model. The compound with OCF_3_ substituent at the fourth position of the aryl group is considered as a promising lead for further development.

**Scheme 25:**
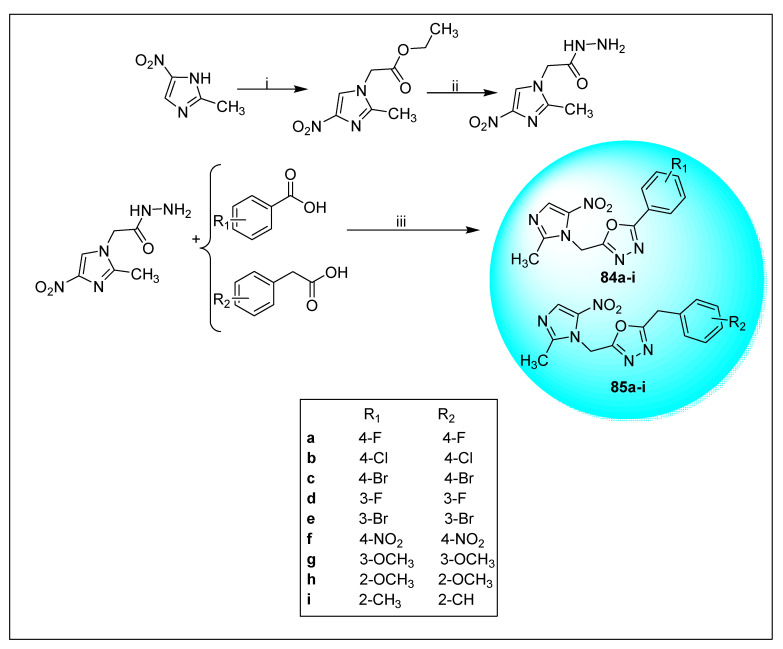
Synthesis of 4-nitroimidazole derivatives with antibacterial activity. Reagents and conditions: (**i**) ClCH_2_COOC_2_H_5_, K_2_CO_3_, acetone; (**ii**) H_2_NNH_2_.H_2_O, MeOH; (**iii**) POCl_3_ (yields not reported).

Li et al. designed and synthesized 4-nitroimidazole derivatives containing 1,3,4-oxadiazole (**84a**–**i**) and (**85a**–**i**) as FabH inhibitor-based anti-microbial activities (Scheme 25) [94]. Among all the synthesized compounds, **84h** and **85i** were proven to be the most potent inhibitors of FabH (IC_50_ of 5.3 and 4.1 µM) along with MIC of 1.56–3.13 µg/mL and 1.56–6.25 lg/mL, respectively, against the tested bacterial strains such as *E. coli*, *P. aeruginosa*, *B. subtilis* and *S. aureus*.

**Scheme 26:**
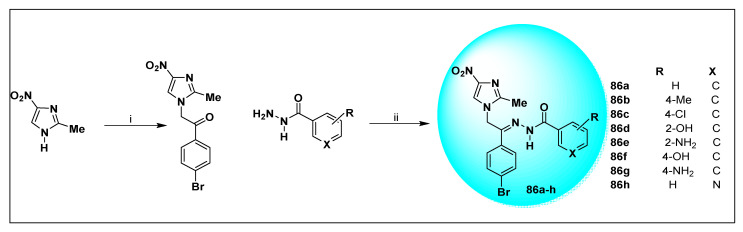
Synthesis of 4-nitroimidazole derivatives as antibacterial, EGFR-based anticancer agents. Reagents and conditions: (**i**) 2-bromo-1-(4-bromophenyl)ethan-1-one, K_2_CO_3_, TBAB, MeCN (yield not reported); (**ii**) Ni(NO_3_)_2_, ethanol, rt, 54–88%.

Based on the same strategy, Makawana et al. developed Schiff’s base derivatives bearing nitroimidazole moiety (**86a**–**h**) (Scheme 26) [95] and screened against anti-bacterial as well as EGFR inhibitory activity with the goal to develop a more effective target molecule. Among these, compounds **86d**, **86f** and **86g** were found to be most effective for antiproliferation and inhibition of EGFR. Conversely, the compounds **86b**, **86c**, **86e** and **86h** were found effective as antibacterial activity. Compound **86f** has shown effective inhibition with an IC_50_ of 0.21 ± 0.02 µM by binding to the active pocket of EGFR receptor with minimum binding energy (ΔG_b_ = −49.4869 kcal/mol).

**Scheme 27:**
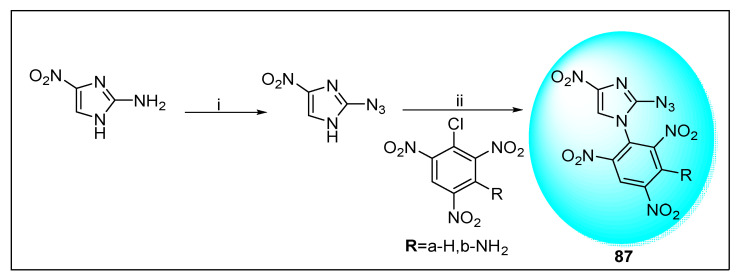
Synthesis of 2-azido-4-nitroimidazoles. Reagents and conditions: (**i**) NaNO_2_, NaN_3_, H_2_O, H_2_SO_4_, 78%; (**ii**) KHCO_3_, TBAB, IPA, 6 h, 85 °C, 72% and 76%.

Hou et al. (2013) synthesized 2-azido-4-nitroimidazole and its derivatives (**87a-b**) for “high-energy materials” (Scheme 27) [96].

**Scheme 28:**
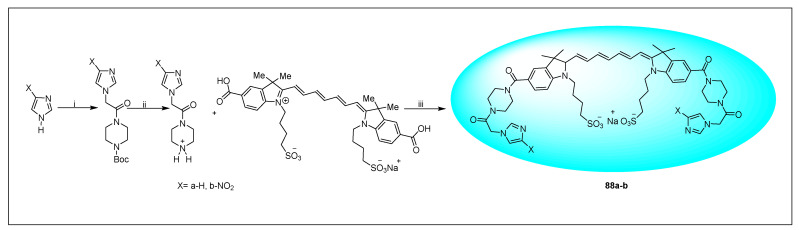
Synthesis of 4-nitromidazole as dye conjugates. Reagents and conditions: (**i**) *tert*-butyl-4-(2-bromoacetyl) piperazine-1-carboxylate, NaH, DMF, 0 °C-rt, 12 h, 67% and 76%; (**ii**) CF_3_COOH, CHCl_3_, 0 °C-rt, 61% and 74%; (**iii**) DMF, 0 °C, pyBop, HOBt, rt, 12 h, 15% and 21%.

Abuteen et al. developed 4-nitroimidazole bearing dye-conjugate (**88a-b**) for the imaging of tumor hypoxia (Scheme 28) [97]. The design is based on the nitroimidazoles property as molecular probes because they diffuse freely in the body and are irreversibly trapped by covalent binding to proteins in low oxygen environments. In the present study, it has been found that the cells treated with **88b** under hypoxic conditions showed a higher fluorescence yield when compared to the cells kept under normoxic conditions.

**Scheme 29:**
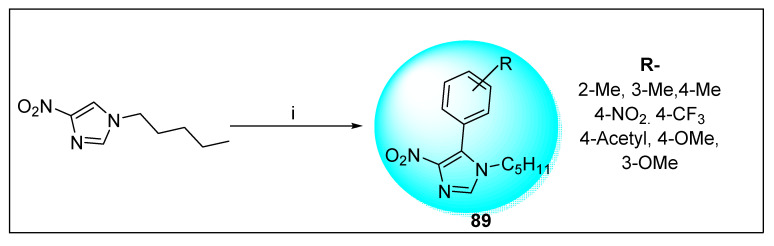
New method for the C–H arylation of 4-nitroimidazoles. Reagents and conditions: (**i**) Cu_2_O/Pd-Fe_3_O_4_ catalyst, 1-butyl-4-nitro-1*H*-imidazole, iodobenzene, sodium acetate, DMSO, 85%.

Woo et al. (2016) developed a new strategy for C–H arylation of 4-nitroimidazoles by using the hybrid Cu_2_O/Pd–Fe_3_O_4_ nanocatalyst system (Scheme 29) [98].

### 2.3. Functionalized 5-Nitroimidazole Scaffold

#### 2.3.1. Metronidazole **7**

**Figure 11:**
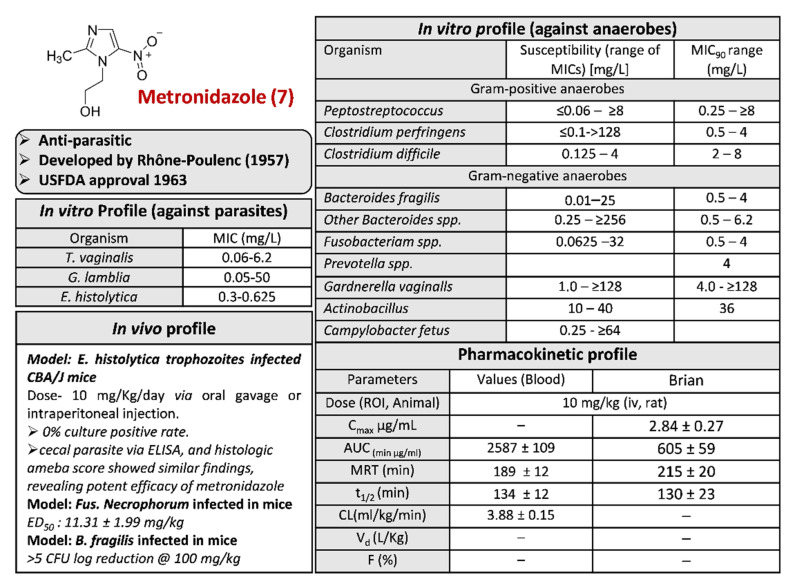
Activity profile of Metronidazole **7** [8,100,101,102].

Metronidazole **7** is generally used as an antibiotic. Its original indication was for the treating infections of trichomoniasis, which is caused by a parasite, namely *Trichomonas vaginalis*. Still, over the years, it has been discovered to be useful in treating a variety of infections caused by various organisms (Figure 11) [2,8,24,99]. Currently, it is frequently used to treat other parasitic infections such as gastrointestinal infections, giardiasis (*G. duodenalis*), and amoebiasis (caused by *E. histolytica*) [100,101]. It has potent activity against a number of Gram-positive and Gram-negative bacterial strains. It has excellent oral absorption with bioavailability often reported as ˃90% [8,102]. The antibacterial action of Metronidazole **7** depends on reduction of its nitro group to form active intermediates. This reduction product then reacts with DNA, disrupting transcription and replication. Metronidazole **7** also reacts with other target sites such as RNA and cellular proteins [103]. Only anaerobic bacteria are capable of performing this reduction, probably through a ferrodoxin system, which could be the reason for the activity of Metronidazole **7** against anaerobes [104]. It is generally available in the form of a capsule, tablet, topical form, and suppository preparations for the management of various infections. Metronidazole **7** was initially developed by Rhône-Poulenc and has been in use since 1960 in France. Metronidazole **7** was approved by the US-FDA in 1963. It is relatively inexpensive and is the safest and most effective medicine needed in a health system with availability in most countries.

**Scheme 30:**
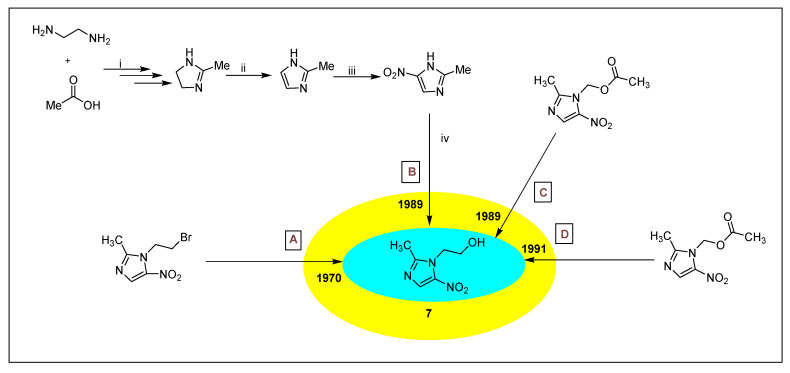
Approaches to prepare Metronidazole **7**. Reagents and conditions: (**A**) formic acid, aqueous formamide, 115 °C, 69%; (**B**) (**i**) 220–250 °C, CaO, 88–95%; (**ii**) Raneynickel, 90%; (**iii**) HNO_3_/H_2_SO_4_, Na_2_SO_4_/NaHSO_4_, 63–66%; (**iv**) H_3_PO_4_/Ac_2_O, 64%; (**C**) *bis*-(2-acetoxyethyl) sulfate/dimethyl sulfate, sulfuric acid, EtOH, reflux (yield not reported); (**D**) ethylene oxide, sulfur trioxide followed by aqueous sulfuric acid, 85 °C (yield not reported).

As shown in Scheme 30, several approaches are described to prepare Metronidazole **7**. Fajdiga et al. (1970) developed a method using bromoethyl imidazole as a starting material. In this case, bromoethyl imidazole was hydrolyzed using formic acid in aqueous formamide (route A) [105]. Kraft et al. (1989) performed the synthesis of metronidazole via formation of 2-methylimidazole, which was in turn prepared using the Debus–Radziszewski approach (route B). In this, ethylenediamine reacted with acetic acid, which led to the formation of diacetic acid salt of ethylenediamine. *N*,*N*’-diacetylethylenediamine, upon treatment with lime, produced 2-methylimidazoline, which was further treated with Raney nickel to produce 2-methylimidaole. 2-Methylimidazole was then nitrated to synthesize 2-methyl-4(5)-nitroimidazole, followed by alkylation, which provided Metronidazole **7** [106]. Buforn et al. (1989) from Rhone Poulenc (route C) synthesized the title compound by alkylation of 1-(acetoxymethyl)-2-methyl-4-nitroimidazole with either ethylene sulfate or by reaction of bis-(2-acetoxyethyl) sulfate (generated from ethylene glycol diacetate) and either dimethyl sulfate or H_2_SO_4_ followed by hydrolysis or alcoholysis treatment [107]. In another attempt, Lavigne et al. (1991) from Rhone Poulenc (route D) synthesized metronidazole by reacting 1-(acetoxy methyl)-2-methyl-4-nitroimidazole with ethylene oxide in the presence of sulfur trioxide followed by hydrolysis in aqueous sulfuric acid [108].

#### 2.3.2. Ornidazole **8**

**Figure 12:**
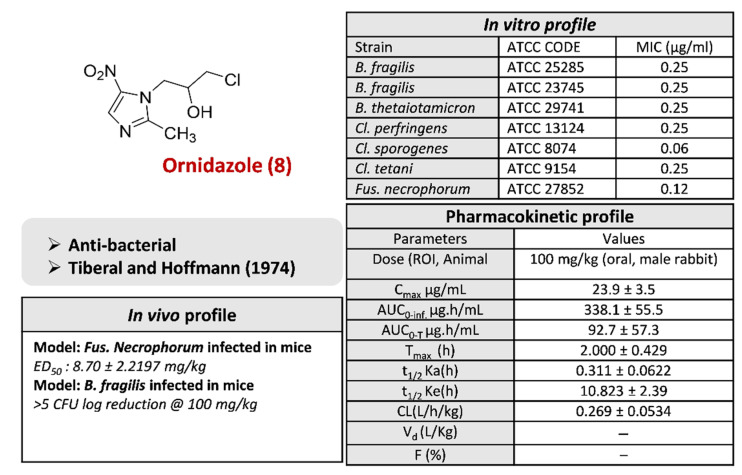
Activity profile of Ornidazole **8** [50,101,109,110,111,113].

Ornidazole **8** (Tiberal^®^) is an antibiotic used to treat protozoan infections (Figure 12). [101,109,110,111] It has been shown to produce successful cures in 87% of cases. It was discovered by Tiberal and Hoffmann in the year 1974 and is marketed by Roche. It was first used for treating trichomoniasis and later on was also recognized for its broad anti-protozoal and anti-anaerobic-bacterial capacities in the micromolar range [50]. Ornidazole **8** has shown pronounced potency against *E. histolytica*, *T. Vaginalis* and *T. foetus* infection models as well as a decent PK profile in comparison to metronidazole. Ornidazole **8** is a 5-nitroimidazole derivative known to have a similar mechanism of action to nitroreduction via bacterial nitroreductases followed by DNA/RNA/protein damage. [111] It is also used in Crohn’s disease after bowel resection [101]. Ornidazole **8** was approved for marketing as a new antimicrobial agent in China in 2009. The Ornidazole **8** used in the clinical setting is mostly racemic, and the number of side effects was observed. Some reports suggested that dextrornidazole is the major component of Ornidazole **8** contributing to the toxicity of the central nervous system. However, Levornidazole is similar to or slightly better than racemic ornidazole in terms of activities and pharmacokinetic properties [112].

**Scheme 31:**
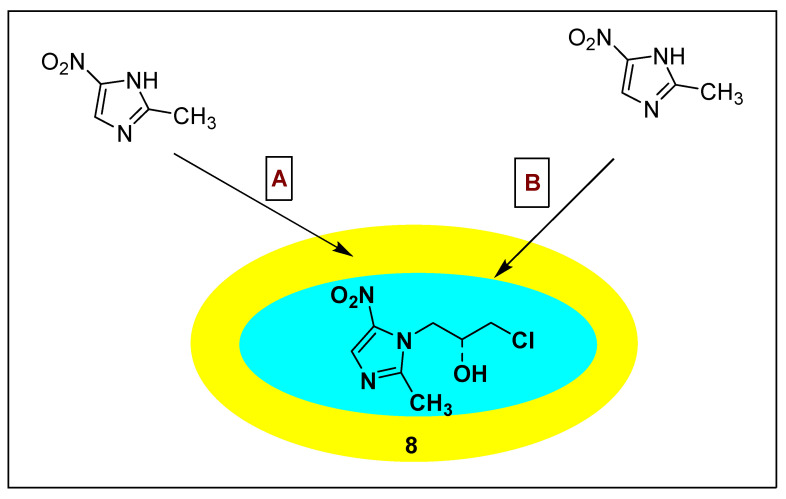
Synthesis of Ornidazole **8**. Reagents and conditions: (**A**) epichlorohydrin, K_2_CO_3_, DMF, 43%; (**B**) AlCl_3_, EtOAc, epichlorohydrin, 0 °C-rt, 24 h, 79%.

Skupin et al. (1997) developed an enzymatic method to synthesize optically pure enantiomers of Ornidazole **8** (Scheme 31, route A). In this method, 2-methyl-4-nitroimidazole on reaction with epichlorohydrin provided halohydrins (ornidazole **8**), which then underwent subsequent esterification with lipase and furnished optical pure isomers of Ornidazole **8** [114]. Mandalapu et al. (2016) synthesized the racemic form of Ornidazole **8** by reacting 2-methyl-(5)-nitroimidazole with epichlorohydrin in basic conditions (route B) [115].

#### 2.3.3. Tinidazole **9**

**Figure 13:**
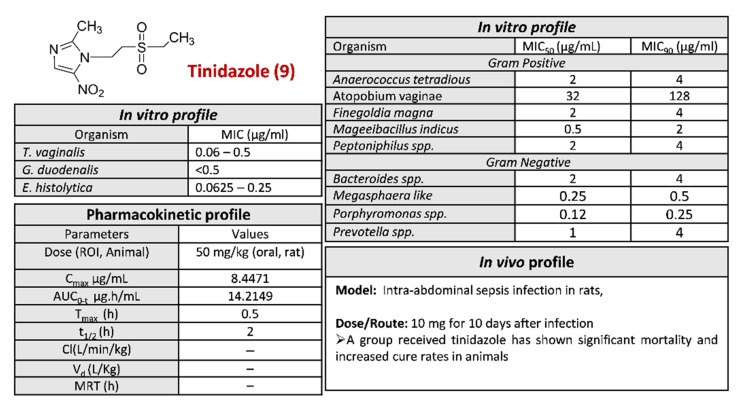
Activity profile of Tinidazole **9** [100,116,118,119].

Tinidazole **9** is a 5-nitroimidazole-based drug effective against trichomoniasis, giardiasis, intestinal amebiasis and amoebic liver abscess infections (Figure 13) [101,116]. Tinidazole **9** is also a prodrug; the nitro group of tinidazole converts into free nitro radical through reduction by a ferredoxin-mediated electron transport system. It also shows antiprotozoal activity. Tinidazole **9** was developed in 1969 and has been widely used in Europe and developing countries for more than two decades with established efficacy and acceptable tolerability [99]. Tinidazole **9** has shown efficacy against protozoal infections (such as trichomonal vaginitis, amoebiasis, and giardiasis) and anaerobic infections (respiratory tract infections, intra-abdominal sepsis, and obstetrical, and gynecological infections. In addition, Tinidazole **9** has also been used as a prophylaxis agent for the treatment of elective colonic and abdominal surgeries, emergency appendectomy, and gynecological surgery, either alone or in combination with other antimicrobial agents [117,118]. It is marketed under the brand names Tindamax and Fasigyn.

**Scheme 32:**
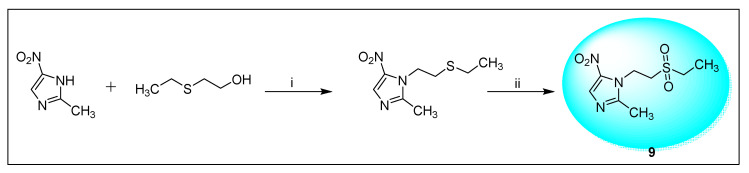
Synthesis of Tinidazole **9**.Reagents and conditions: (**i**) MoO_3_/SiO_2_, H_2_SO_4_, 80–85 °C, 9 h (yield not reported); (**ii**) MoO_3_/SiO_2_, H_2_O_2_ (excess), 70%.

Chandorkar et al. (2007) developed a benign method for the synthesis of Tinidazole **9** (Scheme 32). In this method, 2-methyl-5-nitroimidazole underwent condensation with 2-ethyl-thio-ethanol by using MoO_3_/SiO_2_ as a catalyst and produced 1-(2-ethyl-thio-ethanol)-2-methyl-5-nitroimidazole, which further underwent oxidation in the presence of hydrogen peroxide and MoO_3_/SiO_2_ and produced Tinidazole **9**. The catalyst used in this reaction can be recycled five times without any loss in selectivity [120].

#### 2.3.4. Satranidazole **10**

**Figure 14:**
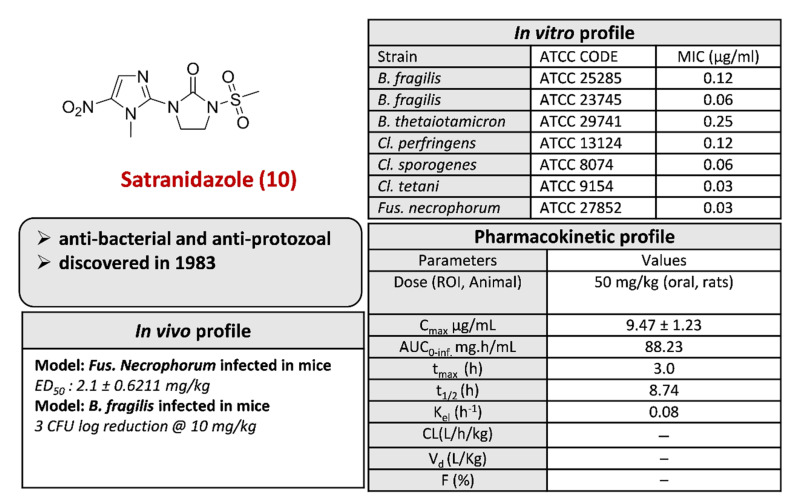
Activity profile of Satranidazole **10** [29,101].

Satranidazole **10** (trade name Satromax) is a potential antibacterial and antiprotozoal drug of the 5-nitroimidazole class used to manage amoebiasis. Satranidazole, also known as an anti-diarrheal agent, inhibits histamine’s action on proteins, thus avoiding the complications of infections such as hepatic amoebiasis. The potent activity of Satranidazole **10** in animal models of anaerobic infection, its long half-life, and a good tolerability observed in volunteers and patients with protozoal infections hold out for a great potential for Satranidazole **10** in the treatment and prophylaxis of anaerobic infections (Figure 14) [22,101].

**Scheme 33:**
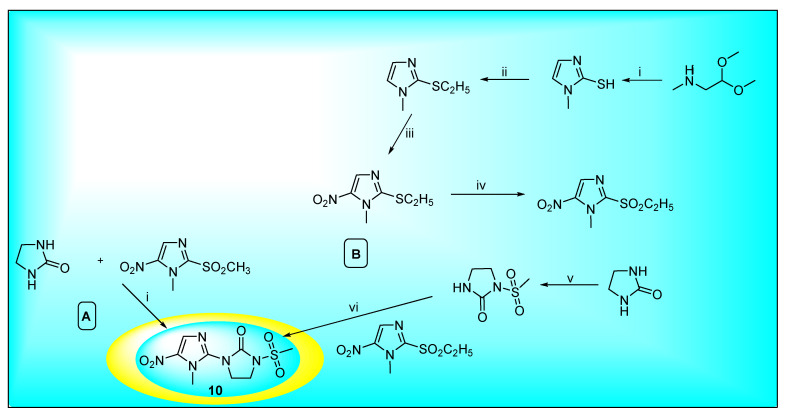
Synthesis of Satranidazole **10**. Reagents and conditions: (**A**) (**i**) dry DMF, NaH, 80%; (**B**) (**i**) KCNS, HCl, (**ii**) C_2_H_5_Br, NaOH, (**iii**) HNO_3_, (**iv**) H_2_O_2_, HCOOH, (**v**) CH_3_SO_2_Cl, (**vi**) NaOH, DMF (yields not reported).

Nagarajan et al. (1982) was the first to synthesize Satranidazole **10** as shown in Scheme 33 (Route A). In route A, 1-methyl-2-(methylsulfonyl)-5-nitroimidazole was condensed with the sodium salt of the monosubstituted 2-imidazolidinone in DMF to furnish Satranidazole **10** in 80% yield [121]. Rao (2003) has patented another route for the synthesis of Satranidazole (route B). Here, the authors synthesized 1-methyl-2-(methylsulfonyl)-5-nitroimidazole starting from *N*-methylamino acetaldehyde dimethylacetal. *N*-methylamino acetaldehyde dimethylacetal was treated with salt of thiocyanic acid followed by in situ alkylation using ethyl bromide to furnish 1-methyl-2-(ethylmercapto)–imidazole. The 1-methyl-2-(ethylmercapto)–imidazole was then treated with nitric acid (70%) to provide 1-methyl-2-(ethylmercapto)–5-nitroimidazole, which was further oxidized by slow addition of 30% hydrogen peroxide. Finally, the condensation of 1-methylsulphonyl-2-imidazolidinone with 1-methyl-2-(ethylsulphonyl)-5-nitroimidazole furnished Satranidazole **10** [122].

#### 2.3.5. Nimorazole **11**

**Figure 15:**
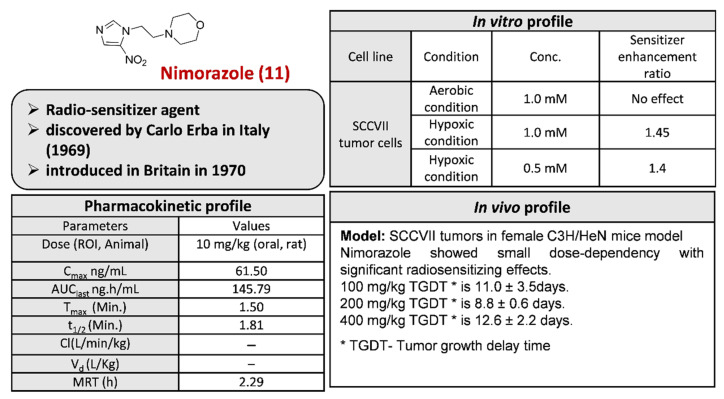
Activity profile of Nimorazole **11** [125,126]. *: TGDT–Tumor Growth delay time.

Nimorazole **11**, previously known as nitrimidazine, was the second nitroimidazole introduced as a radiosensitizer and is known as an anti-infective and anti-protozoal (against trichomoniasis) agent (Figure 15). Nimorazole **11** has been used in trials for the treatment of hypoxia, radiotherapy, hypoxic modification, gene profile, gene signature, head and neck squamous cell carcinoma, etc. It was discovered by the research workers of Carlo Erba in Italy in the year 1969. It was introduced in Britain in 1970 [14].

**Scheme 34:**
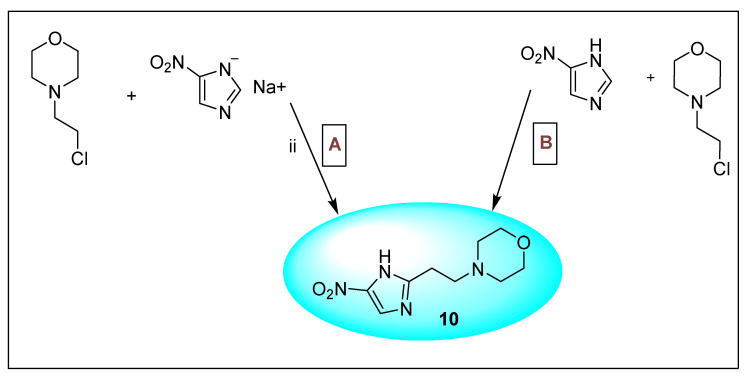
Synthesis of Nimorazole 11. Reagents and conditions: (**A**) (**ii**) sodium salt of nitroimidazole, dry toluene reflux (yield not reported); (**B**) K_2_CO_3_, toluene, reflux, methanol, 99%.

Nicola and Vittorio (1968) from Carlo Erba developed a route for the synthesis of Nimorazole **11** (Scheme 34) by coupling sodium salt of nitroimidazole with *β*-chloroethyl morpholine [123]. Naik in 2012 developed another modified route for the synthesis of Nimorazole **11** [124].

#### 2.3.6. Secnidazole **12**

**Figure 16:**
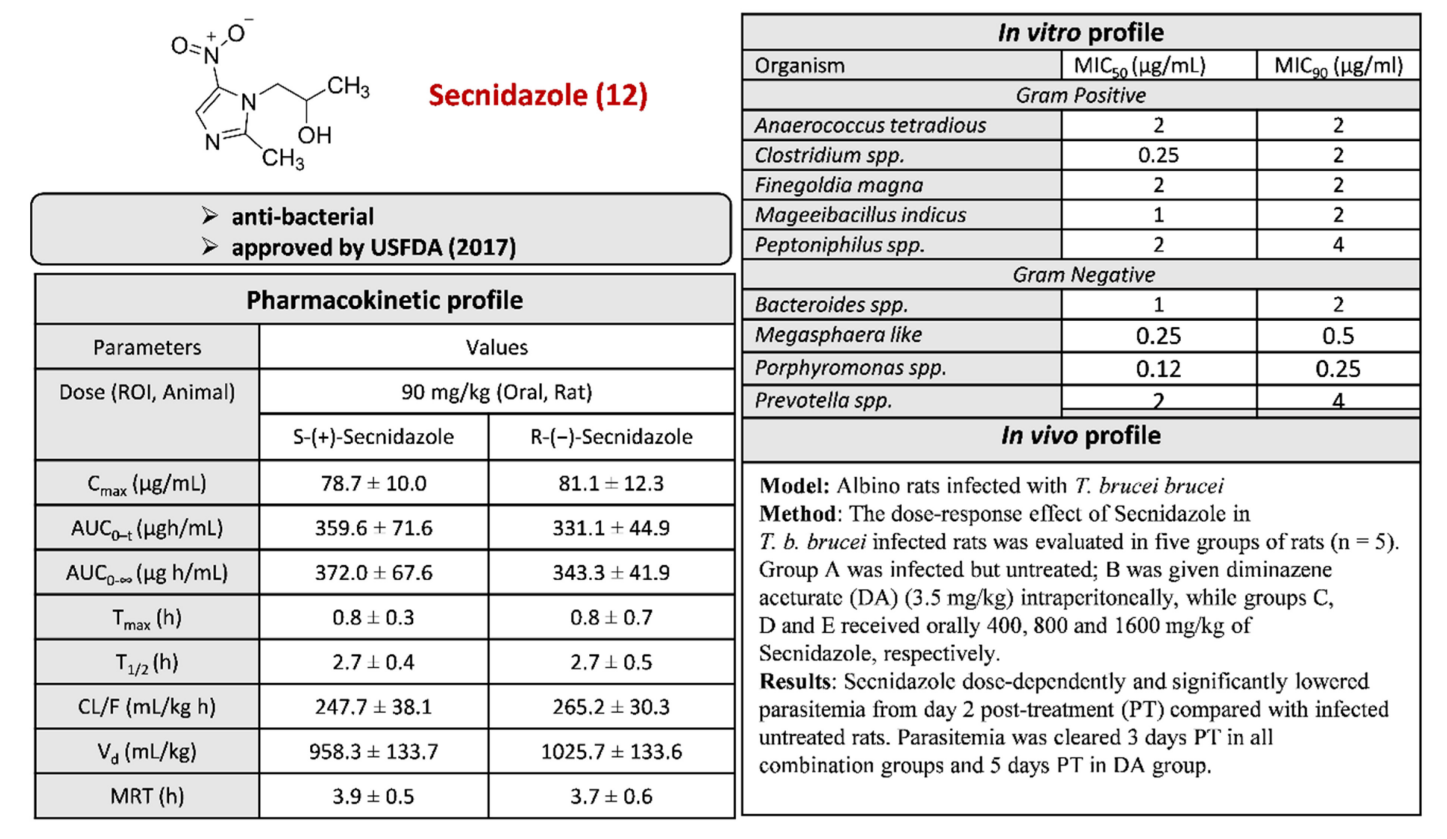
Activity profile of Secnidazole **12** [127,128,129].

Secnidazole **12** is a second-generation antimicrobial and is structurally related to Metronidazole **7** and Tinidazole **9**. Secnidazole displayed improved oral absorption and a longer terminal elimination half-life than antimicrobial agents in this class (Figure 16). Secnidazole **12** is safe and well tolerated, and it is widely used for amoebiasis, giardiasis, trichomoniasis, and genitourinary infections [97]. The antiprotozoal and anti-amoebic activities of Secnidazole **12** are due to the reduction of the nitro group of nitroimidazole by ferredoxin [127]. Secnidazole **12** is completely absorbed after oral administration. Secnidazole has been available in many other countries for decades and was recently approved in the United States (2017) for bacterial vaginosis therapy.

**Scheme 35:**
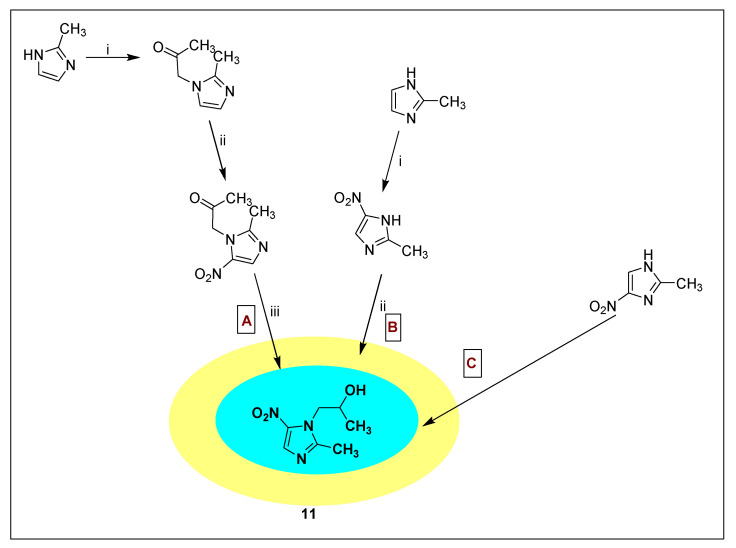
Synthesis of Secindazole **12**. Reagents and conditions: (**A**) (**i**) Chloroacetyl chloride, Potassium carbonate, acetone, reflux (**ii**) Nitric acid, P_2_O_5_, (**iii**) sodium borohydride, MeOH, rt (yields not reported); (**B**) (**i**) H_2_SO_4_, HNO_3_, (**ii**) epoxypropane, ethanol, 20 °C/chloroacetyl chloride, potassium carbonate, acetone, reflux (yield not reported); (**C**) epichlorohydrin, AlCl_3_, EtOAc, 1.5 h, 4 5 °C, 75–80%.

Three routes are available for the synthesis of Secnidazole **12** (Scheme 35). In route A, Jeanmart et al. from Rhone Poulenc patented a route for the synthesis of Secnidazole by reacting 2-methyl imidazole with chloroacetyl chloride to obtain (2-methylimidazol-1-yl)acetone, which upon reaction with HNO_3_ and P_2_O_5_, produced the corresponding nitro compound. Finally, this product upon reduction with NaBH_4_ produced Secnidazole **12** [130]. Hillier et al. (1979) from Rhone Poulenc patented another route (route B), which involved the nitration of 2-methyl imidazole with HNO_3_ and H_2_SO_4_ followed by condensation with 1-chloro-isopropanol or with propylene oxide to produce Secnidazole **12** [131]. Kuang et al. (2020) from Faming Zhuanli Shenqing developed another route (route C) for the synthesis of Secnidazole **12**. Here, 2-methyl-5-nitroimidazole was reacted with epichlorohydrin to obtain the required product **12** [132].

#### 2.3.7. Dimetridazole **13**

**Figure 17:**
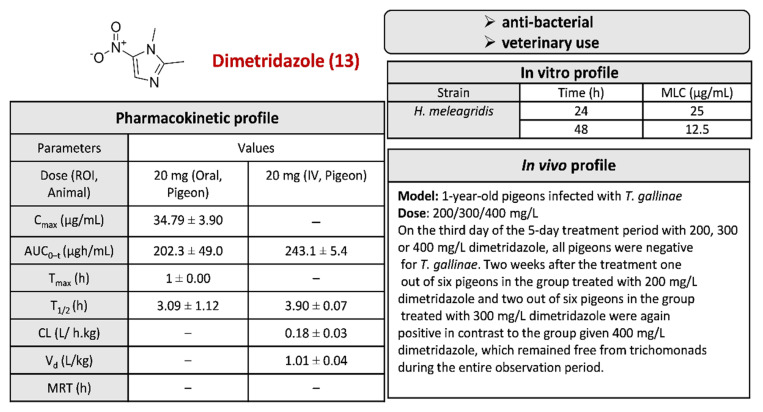
Activity profile of Dimetridazole **13** [133,134].

**Figure 18:**
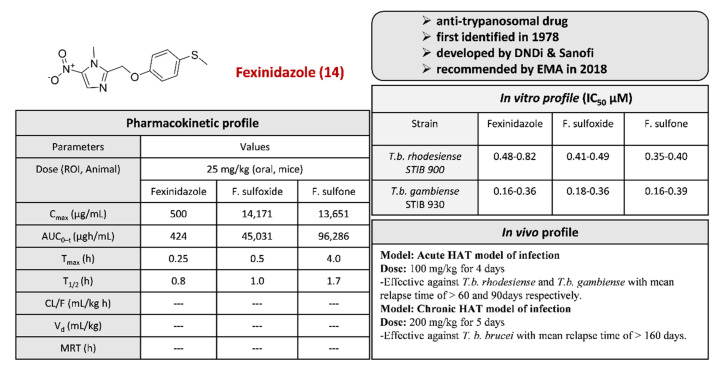
Activity profile of Fexinidazole **14** [10].

Dimetridazole **13**, another 5-nitroimidazole, has been developed and used since the 1960s to treat and prevent histomoniasis (a disease caused by protozoan flagellate *Histomonas meleagridis*) and coccidiosis (parasitic disease of animal caused by coccidian protozoa) in poultry and game birds (Figure 17). It has also been used for the treatment of genital trichomoniasis in cattle and hemorrhagic enteritis in pigs. Dimetridazole **13** on testing has shown good in vitro minimum lethal concentrations (MLC), which is used to determine drug efficacy and parasite viability after removal of residual drugs, and the data are presented in Figure 18. Dimetridazole **13** has shown oral PK exposure and efficacy in *T. gallinae*-infected pigeon models [133]. However, Dimetridazole **13** was banned in the European Council and the US in 1995 and 1997, respectively, because of its carcinogenic nature.

**Scheme 36:**
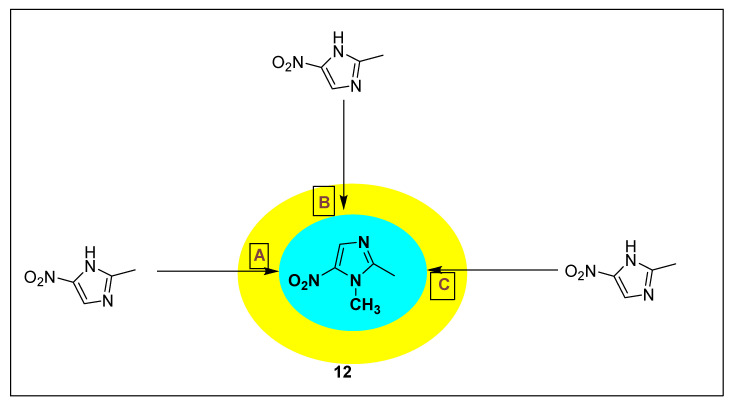
Synthesis of Dimetridazole **13**. Reagents and conditions: (**A**) Dimethyl sulfate, HCOOH, 4 h, 87%; (**B**) MeI, Cs_2_CO_3_, DMF (yield not reported); (**C**) dimethyl sulfate, H_2_SO_4_, MeOH, 95 °C, 5 min, 97%.

As shown in Scheme 36, in 2013, the first attempt to synthesize dimetridazole **13** was performed by refluxing 2-methyl-4-nitroimidazole with dimethyl sulfate (route A) [135]. In 2017, Estrada et al. from Denali Therapeutics INC (US) synthesized Dimetridazole **13** by treating 2-methyl-4-nitroimidazole with methyl iodide (route B) [136]. Later, in 2018, Yao, F. et al. developed a new method to synthesize Dimetridazole **13** (route C) by reacting 2-methyl-4-nitroimidazole with dimethyl sulfate at a better yield (87%) [137].

#### 2.3.8. Fexinidazole 14

Fexinidazole **14**, is used for the treatment of African human trypanosomiasis (HAT), commonly known as sleeping sickness and Chagas disease. Fexinidazole **14** shows excellent in vitro activity against *Tb. rhodesiense* and *Tb. gambiense*, having IC_50_ value ranges from 0.48–0.85 μM and 0.16–0.36 μM, respectively (Figure 18) [10]. Fexinidazole **14** becomes metabolized into fexinidazole sulfoxide (M1) and fexinidazole sulfone metabolite (M2), and these metabolites have in vitro activity with IC_50_ values within the μM range. The pharmacokinetic study of Fexinidazole **14** and their metabolites M1 and M2 showed good plasma exposure and in vivo activity. Fexinidazole **14** was first identified in 1978 and developed by DNDi (Drugs for Neglected Diseases Initiative) in collaboration with Sanofi for sleeping sickness and was recommended by the European Medicines Agency in 2018 [138,139]. In 2019, Fexinidazole **14** was added to the WHO’s list of essential medicines due to its efficacy and safety profile [73,84].

**Scheme 37:**
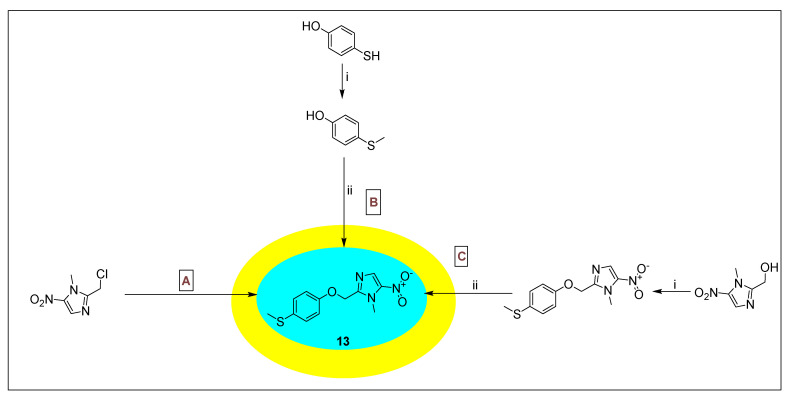
Synthesis of Fexinidazole **14**. Reagents and conditions: (**A**) 4-methyl mercaptophenol, MeCN, Ar, 80 °C, 24 h, 60%; (**B**) (**i**) methyl iodide, trimethylamine, dry THF, rt, 36%; (**ii**) 2-(chloromethyl)-1-methyl-5-nitro-1*H*-imidazole, Cs_2_CO_3_, DMF, 0 °C, rt, 3 h, 28%; (**C**) (**i**) sulfonyl chloride, K_2_CO_3_, acetone, rt; (**ii**) 4-methyl meracptophenol, acetone.

As shown in Scheme 37, the first attempt to synthesize Fexinidazole **14** was made by Samant et al. in 2011 (route A). In this pathway, 2-(chloromethyl)-1-methyl-5-nitro-1H-imidazole was treated with 4-methyl mercaptophenol in acetonitrile under argon atmosphere to furnish Fexinidazole **14** in 60% yield [140]. In 2011, Fexinidazole **14** was synthesized by Fontana et al. (route B), wherein 4-methyl mercaptophenol was first treated with methyl iodide in the presence of trimethylamine in dry THF to obtain 4-(methylthio)phenol, which upon further treatment with 2-(chloromethyl)-1-methyl-5-nitro-1*H*-imidazole in dimethyl formamide, produced deuterium-labeled Fexinidazole **14** [141]. In 2014, Zsolt et al. from Drugs for Neglected Diseases Initiative (DNDI) (CH) patented another route (route C) for the synthesis of Fexinidazole **14**. Here, 1-methyl-2-hydroxymethyl-5-nitroimidazole was reacted with sulfonyl chloride in the presence of potassium carbonate to obtain 1-methyl-2-((4-(methylthio)phenoxy)methyl)-5-nitro-1*H*-imidazole, which upon further treatment with 4-methyl meracptophenol, furnished Fexinidazole **14** [142].

#### 2.3.9. Megazol **15**

**Figure 19:**
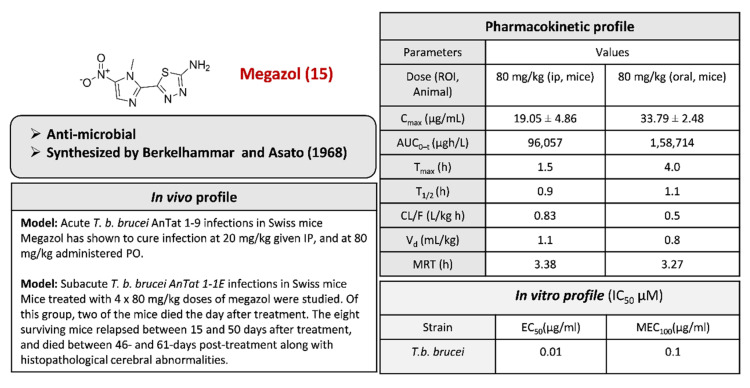
Activity profile of Megazol **15** [144,145].

Megazol **15** was developed as an anti-microbial agent and was first synthesized by Berkelhammer and Asato from the American Cyanamid Company in 1968. Megazol **15** was tested against a wide variety of Gram-negative and Gram-positive bacteria in chicks and mice as well as against a number of parasitic infections in rodents and was found as effective as furazolidone. The effective oral dose is between 1 and 90 mg/kg, depending upon the microbe [143]. Megazol **15** has also shown activity against Human African trypanosomiasis (HAT) or sleeping sickness with in vitro activity against *T. b. brucei* with an EC_50_ of 0.01 µg/mL and was found to be effective in curing the acute disease condition (Figure 19) [144]. Megazol **15** has good oral exposure with the highest AUC and C_max_ values when compared to the intraperitoneal route [145].

**Scheme 38:**
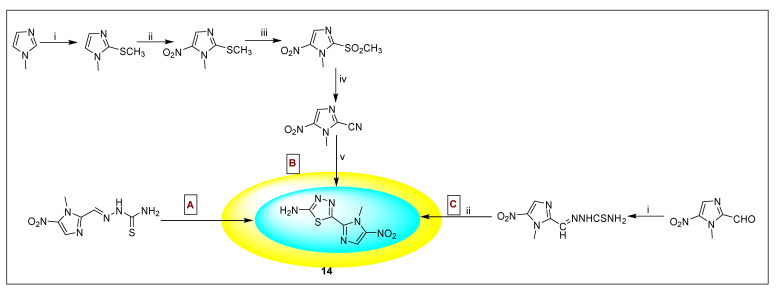
Synthesis of Megazol **15**. Reagents and conditions: (**A**) NH_4_Fe(SO_4_)_2_, hot water, 81%; (**B**) (**i**) BuLi, −30 °C, (CH_3_S)_2_, 100%; (**ii**) HNO_3_, 70 °C, 45%, (**iii**) H_2_O_2_, CF_3_COOH, 90%; (**iv**) KCN, DMSO, 80%; (**v**) H_2_NNHCSNH_2_, 50%; (**C**) (**i**) thiosemicarbazide, ethanol, reflux; (**ii**) ammonium ferric sulfate, H_2_O, reflux (yields not reported).

As shown in Scheme 38, the first attempt to synthesize Megazol **15** was performed by Berkelhammer et al. in 1968 (route A) [143]. In this route, Megazol **15** was synthesized by ferric ammonium sulfate catalyzed oxidative cyclization of 1-methyl-5-nitroimidazole-2-carboxaldehyde thiosemicarbazone in hot water. In 2003, Chauviere et al. proposed a new synthetic route for the synthesis of Megazol **15** (route B) [146]. Here, the carbanion at position 2 of 1-methylimidazole was quantitatively thiomethylated with dimethyl disulfide followed by nitration to furnish the corresponding 5-nitroimidazole. Then, oxidation by hydrogen peroxide led to the sulfone where a nucleophilic substitution by cyanide anion produced the corresponding carbonitrile. Finally, a condensation with thiosemicarbazide in trifluoroacetic acid followed by cyclization and isomerization produced Megazol **15**. In 2008, Foroumadi et al. synthesized Megazol **15** from 1-methyl-5-nitro-1H-imidazole-2-carbaldehyde (route C) [147], wherein 1-methyl-5-nitro-1*H*-imidazole-2-carbaldehyde was refluxed with thiosemicarbazide in ethanol, producing 2-((1-methyl-5-nitro-1H-imidazol-2-yl)methylene)hydrazine-1-carbothioamide, which upon further reaction with ferric ammonium sulfate, furnished Megazol **15**.

#### 2.3.10. Carnidazole **16**

**Figure 20:**
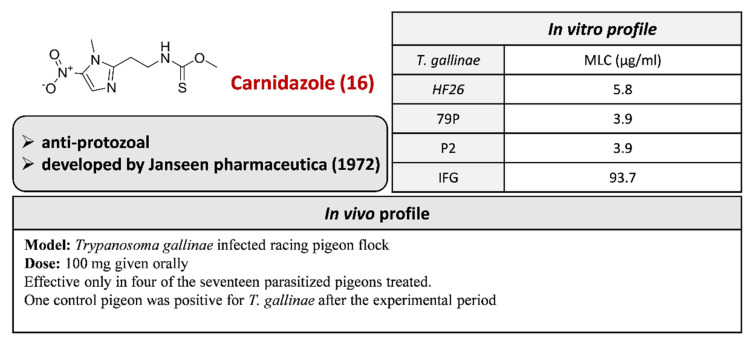
Activity profile of Carnidazole **16** [149].

Carnidazole **16** (trade name Spartrix) is an antiprotozoal drug of the nitroimidazole class. It is found to be highly effective against trichomoniasis (Figure 20). Carnidazole has been used in Belgium since December 1974 as veterinary medicine, mainly in pigeons. Carnidazole **16** was also tried in human trichomoniasis due to its good efficacy proven in animals. The first clinical trials of Carnidazole **16** (given orally) in Brazil (Nogueira, 1975) showed a high percentage of gastrointestinal side effects [148].

**Scheme 39:**
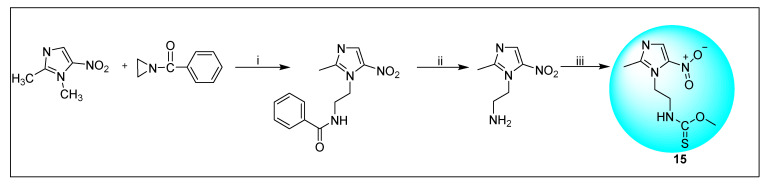
Synthesis of Carnidazole **16**. Reagents and conditions: (**i**) BF_3_.Et_2_O, HOAc, CHCl_3_, −10 to 10 °C; (**ii**) 48% HBr solution, reflux; (**iii**) O-methylcarbonochloridothioate, NaHCO_3_, toluene (yields not reported).

Janseen Pharmaceutica in 1977 patented a route for the synthesis of Carnidazole **16** from 1,2-dimethyl-5-nitroimidazole (Scheme 39). The reaction of 1,2-dimethyl-5-nitroimidazole with 1-benzoylaziridine produced a benzoylated intermediate, which upon refluxing with hydorbromic solution, produced 2-(2-methyl-5-nitroimidazol-1-yl)ethan-1-amine, which upon further reaction with *O*-methylcarbonochloridothioate, produced the final product **16** [150].

#### 2.3.11. Miscellaneous 5-Nitroimidazole Derivatives

**Scheme 40:**
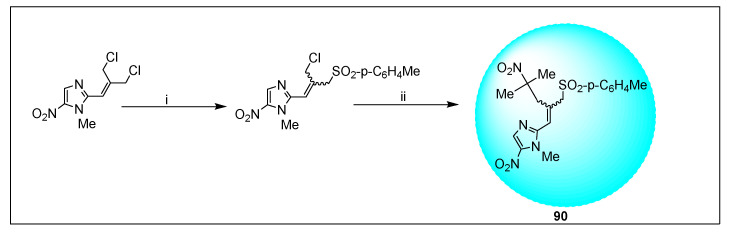
Synthesis of 5-nitroimidazole-based sulfones as antibacterial agents. Reagents and conditions: (**i**) sodium *p*-toluenesulfinate, DMSO, rt, *E*/*Z*-70%; (**ii**) TBHP, Me-C_6_H_5_, H_2_O, hv, *E*-40%, Z-20%.

Benakli et al., in the year 2002, developed the synthesis of 5-nitroimidazole-based sulfones (**90**), having activity against metronidazole-susceptible and -resistant *Giarda*, *Trichomonas*, and *Entamoeba* spp. (Scheme 40) [151].

**Scheme 41:**
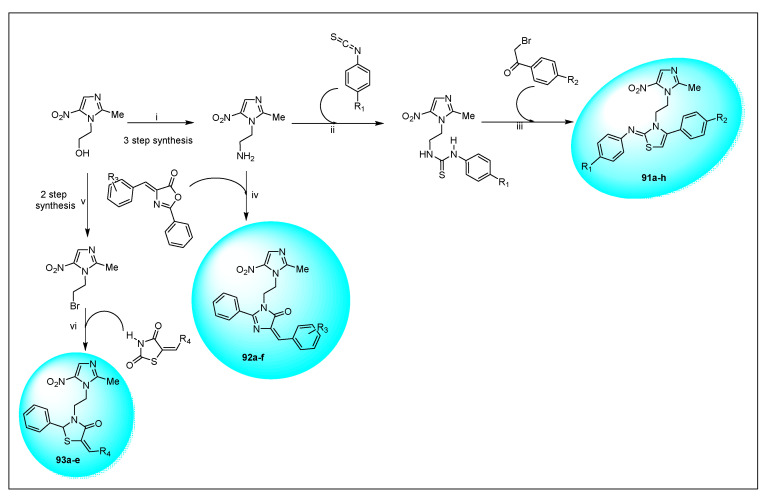
Synthesis of 5-nitroimidazole derivatives as antimicrobial agents. Reagents and conditions: (**i**) MeSO_2_Cl, pyridine then NaN_3_, DMF, and then PPh_3_, THF, and then HCl, H_2_O (yield not reported); (**ii**) MeCOONa, EtOH, reflux, 69–73%; (**iii**) NaHCO_3_, EtOH, reflux, 69–82%; (**iv**) MeCOONa, MeCO_2_H, reflux, 45–50%; (**v**) MeSO_2_Cl, Pyridine then KBr, DMF (yield not reported); (**vi**) K_2_CO_3_, MeCOMe, reflux, 62–83%.

Benkli et al. (2003) obtained some new nitroimidazole derivatives from 2-(2-methyl-5-nitro-l*H*imidazol-l-yl)ethylamine dihydrochloride and 1-(2-bromoethyl)-2-methyl-5-nitroimidazole, prepared using metronidazole. 2-(2-Methyl-5-nitro-l*H*imidazol-l-yl)ethylamine dihydrochloride underwent reaction with arylisothiocyanates to produce 1-[2-(2-methyl-5-nitroimidazol-l-yl)ethyl]-3-arylthioureas, and the intermediate was then reacted with bromoacetophenones to furnish 3-[2-(2-methyl-5-nitroimidazol-l-yl)ethyl]-2-arylimino-4- aryl-4-thiazolines as the final product (**91a–h**) (Scheme 41). In addition, 1-[2-(2-methyl-5-nitroimidazol-l-yl)ethyl]-2-phenyl-4-arylideneimidazolin-5-ones were prepared (**92a–f**) by reacting 2-(2-methyl-5-nitro-l*H*-imidazol-l-yl)ethylamine dihydrochloride with 2-phenyl-4-arylidene-5-oxazolones. The reaction of 1-(2-bromoethyl)-2-methyl-5-nitroimidazole with 5-arylidenethiazolidin-2,4-dione produced 3- [2-(2-methyl-5-nitroimidazol-l-yl)ethyl]-5-arylidenethiazolidin-2,4-dione derivatives (**93a–e**). The synthesized derivatives showed moderate activity [152].

**Scheme 42:**
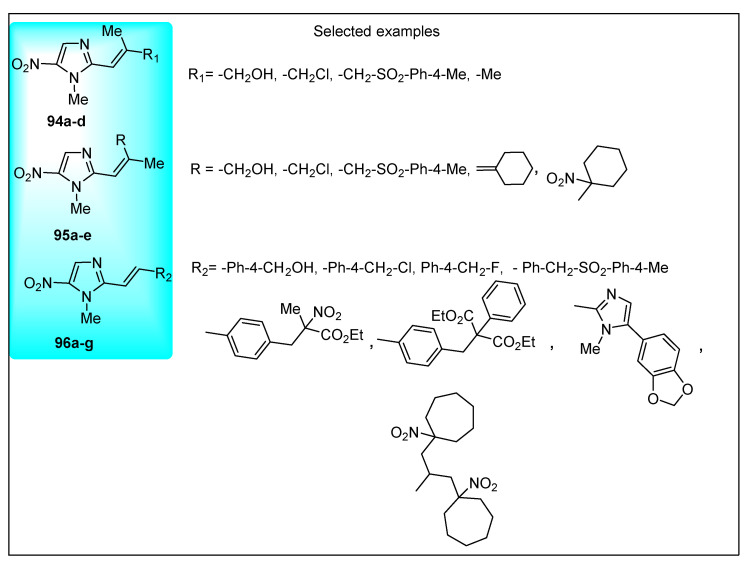
Synthesis of 5-nitroimidazoles as anti-parasitic drugs. Valdez et al. (2009) synthesized the 2-ethenyl and 2-ethanyl derivatives of 5-nitroimidazole (**97a–z**’) as antimicrobial agents against *Giardia lamblia* (Scheme 43) [154].

Upcroft et al. in 2006 performed a study that provided the motive for the continued design of 5-nitroimidazole drugs (**94a–d**, **95a–e**, **96a–g**), to neglect the cross-resistance among established 5-nitromidazole anti-parasitic drugs (Scheme 42). One of the newly synthesized compounds showed activity against metronidazole (Mz)-resistant *Giardia* and *Trichomonas strains*. In addition to this, five other compounds were also found effective against some of the Mz-resistant parasites [153].

**Scheme 44:**
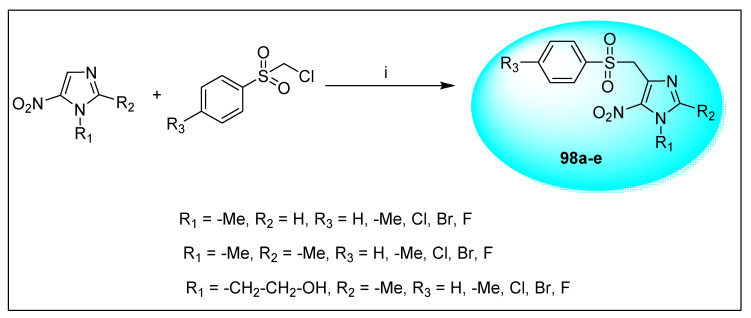
Synthesis of (arylsulfonylmethy)-5-nitroimidazoles as antiparasitic agents. Reagents and conditions: (**i**) KOH/DMSO, 17–54%.

Crozet et al., in 2009, to improve the anti-parasitic pharmacophore, prepared twenty 5-nitroimidazoles (**98a–e**) bearing an arylsulfonylmethyl group from commercial imidazoles (Scheme 44) [155]. These molecules were tested for antiparasitic activity against *Trichomonas vaginalis*. The in vitro cytotoxicity and the mutagenicity of these compounds were also evaluated. All the compounds showed lower IC_50_ values against *T. vaginalis* than metronidazole. Moreover, 11 derivatives had a better safety index (SI) than metronidazole. The results also revealed that those molecules with an additional methyl group on the 2-position were less mutagenic than metronidazole. The present study provided three derivatives with low mutagenicity and efficient anti-trichomonas activity.

**Scheme 45:**
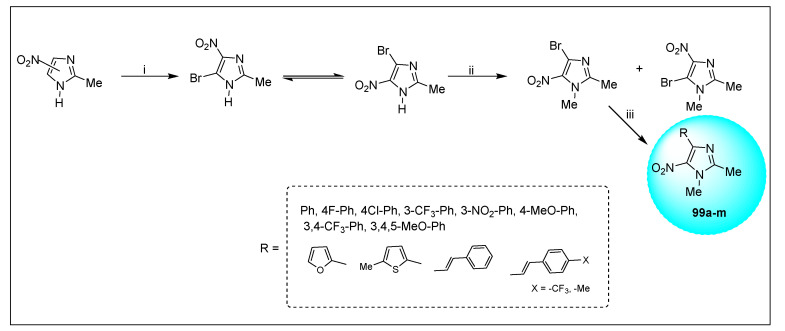
Efficient microwave-assisted palladium-catalyzed Suzuki–Miyaura cross-coupling reactions in 5-nitroimidazole series. Reagents and conditions: (**i**) Br_2_, Na_2_CO_3_, DMF, 70 °C, 2 h, 93%; (**ii**) DMS, DMF, 100 °C, 2.5 h, 23% and 24% (**iii**) RB(OH)_2_, Pd(PPh_3_)_4_, Na_2_CO_3_, MW 150W, DME-EtOH, 68–98%.

Crozet et al. (2009) developed the novel various aryl, heteroaryl- and styryl-based 5-nitroimidazole derivatives (**99a–m**) via microwave-assisted palladium-catalyzed Suzuki–Miyaura cross-coupling reaction (Scheme 45) [156].

**Scheme 46:**
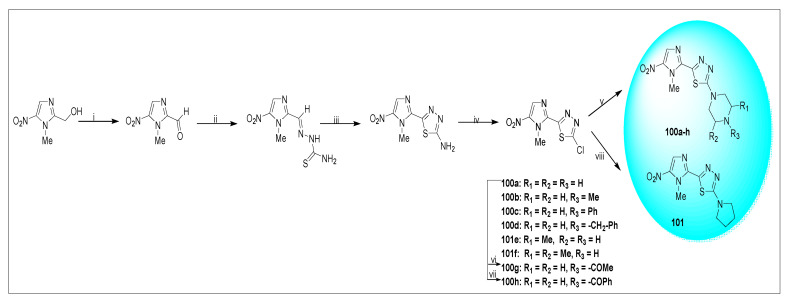
Synthesis of 5-nitroimidazole-based 1,3,4-thiadiazoles as antibacterial agents. Reagents and conditions: (**i**) MnO_2_, CHCl_3_, rt; (**ii**) Thiosemicarbazide, EtOH, HCl, reflux; (**iii**) NH_4_Fe(SO_4_)_2_, H_2_O, reflux then NaNO_2_, HCl, Cu, 0 °C; (**iv**) NaNO_2_, HCl, Cu, 0 °C → rt., 3 h; (**v**) Piperazine derivatives, NaHCO_3_, EtOH, reflux, 25–89%; (**vi**) *N*-Ethyl diisopropylamine, acetyl chloride, THF, reflux; (**vii**) PhCOCl, pyridine in dry benzene, 0 °C → rt; (**viii**) Pyrrolidine, NaHCO_3_, EtOH, reflux, 53%.

Moshafi et al. in 2011 prepared a series of 5-nitroimidazole-based 1,3,4-thiadiazoles (**100a–h** and **101**), and the antibacterial activity of these molecules was assessed against *Helicobacter pylori* (Scheme 46) [157] and compared with antimicrobial metronidazole. The activity results of the synthesized compounds against 20 clinical isolates revealed that five derivatives having piperazinyl, 4-methylpiperazinyl, 3-methylpiperazinyl, and 3,5-dimethylpiperazinyl analogs (**100a**, **100b**, **100e**, and **100f**, respectively) and pyrrolidine derivative **101** have shown strong activity at 0.5 mg/disc (average of inhibition zone >20 mm), while metronidazole had no activity at this dose. Compound **100f** having the 3,5-dimethylpiperazinyl moiety was the most potent compound tested at low concentrations and produced a new promising lead for developing an effective anti-*Helicobacter* agent.

**Scheme 47:**
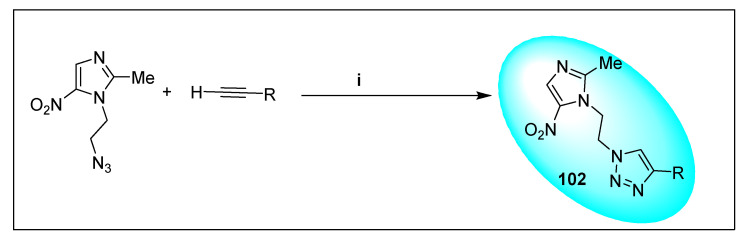
Synthesis of 5-nitroimidazoles as antimicrobial agents. Reagents and conditions: (**i**) CuSO_4_/NaAsc, *t*-BuOH:H_2_O (yield not reported).

Miyamoto et al. (2013) developed the next-generation 5-nitroimidazole-based analogs **102** as antimicrobial agents with broad structural diversity (Scheme 47). In this, the authors had synthesized more than 650 compounds with structural diversity in various functional groups and found compounds with improved activity against various microbes, including the pathogenic protozoa *Giardia lamblia* and *Trichomonas vaginalis*, and the bacterial pathogens *Helicobacter pylori*, *Clostridium difficile*, and *Bacteroides fragilis* [158].

**Scheme 48:**
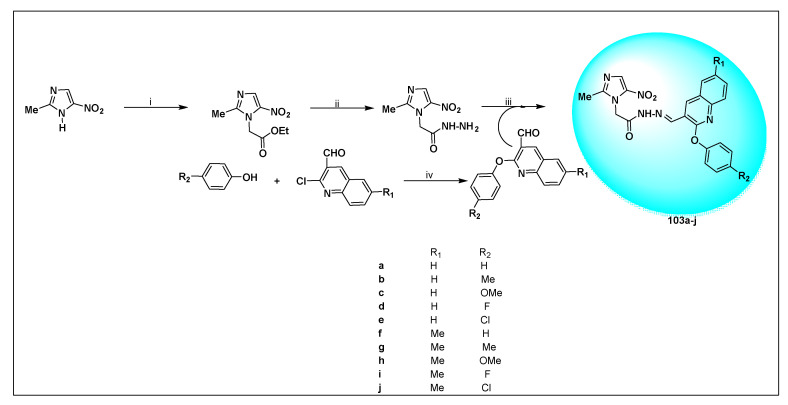
5-Nitroimidazole derivatives as anticancer agents. Reagents and conditions: (**i**) ClCH_2_CO_2_Et, K_2_CO_3_, MeCOMe (yield not reported); (**ii**) NH_2_-NH_2_.H_2_O, MeOH; (**iii**) EtOH, reflux, 78-92%; (**iv**) DMF, K_2_CO_3_ (yield not reported).

Makawana et al. (2014) synthesized new Schiff’s base derivatives (**103a**–**j**) by reaction between 2-phenoxyquinoline-3-carbaldehydes and 2-(2-methyl-5-nitro-1*H*-imidazol-1-yl)acetohydrazide (Scheme 48). All compounds were evaluated for anticancer activity and EGFR inhibition, and the results revealed that the majority of the compounds showed effective anti-proliferation and inhibition of EGFR and HER-2 activities [159]. In this study, compound **103d** showed the most effective inhibition with an IC_50_ of 0.37 ± 0.04 µM.

**Scheme 49:**
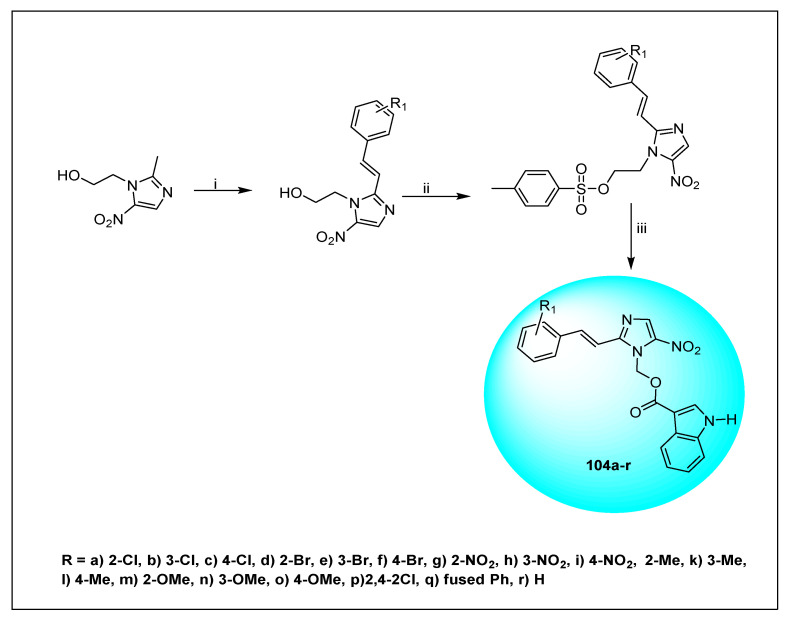
Synthesis of 1-indolyl acetate-5-nitroimidazoles as anticancer agents. Reagents and conditions: (**i**) Sodium methoxide, different aldehydes, DMSO, methanol, room temperature, 4 h, 63–83%; (**ii**) 4-toluene sulfonyl chloride; CH_2_Cl_2_, TEA, room temperature, 5 h, 62–79%; (**iii**) Indole-3-carboxylic acid, DMF, K_2_CO_3_, reflux, overnight, 62–68%.

Duan et al. (2014) designed and synthesized series of 18 novel 1-indolyl acetate-5-nitroimidazole derivatives (**104a–r**) and assessed their activity for potential tubulin polymerization inhibitors (Scheme 49) [160]. (*Z*)-2-(2-(2,4-dichlorostyryl)-5-nitro-1*H*-imidazol-1-yl)ethyl 1*H*-indole-3-carboxylate (**104o**) has shown strong antitumor activity against A549, Hela and U251 with an IC_50_ of 2.00, 1.05, 0.87 μM, respectively. The compound **104o** was found to inhibit PLK1 activity with an IC_50_ of 2.4 μM.

**Scheme 50:**
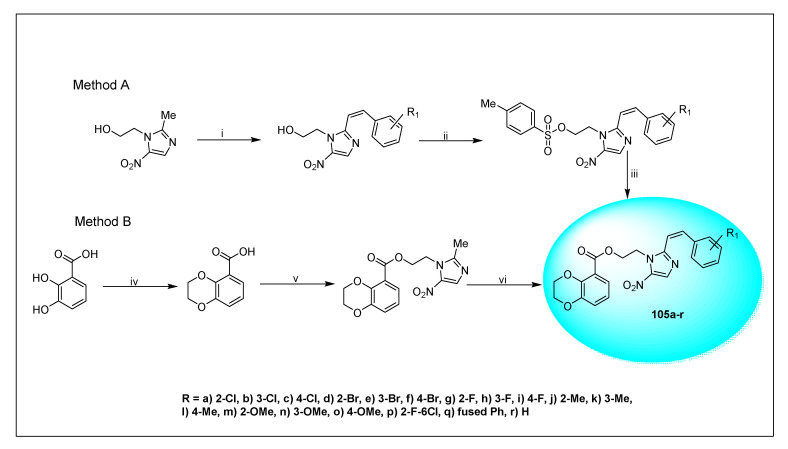
Synthesis of 2-styryl-5-nitroimidazole derivatives. Reagents and conditions: (**i**) NaOMe, aldehydes, DMSO, MeOH, rt (yield not reported); (**ii**) 4-toluene sulfonyl chloride, DCM, TEA, rt (yield not reported); (**iii**) 2,3-dihydro-1,4-benzodioxine-5-carboxylic acid, DMF, K_2_CO_3_, reflux, 61–68%; (**iv**) MeOH, H_2_SO_4_, 90 °C, DCM, K_2_CO_3_, MeCOMe, 70 °C then NaOH (aq), MeOH, THF, 85–95%; (**v**) SOCl_2_, DMF, reflux then DCM, TEA; (**vi**) NaOH, different aldehydes, DMSO, MeOH, rt, 61–68%.

Duana et al. (2014) synthesized series of 2-styryl-5-nitroimidazole derivatives (**105a–r**) containing 1,4-benzodioxane moiety and them evaluated for biological activities as anti-proliferation and focal adhesion kinase (FAK) inhibitors (Scheme 50) [161]. Among them all, compounds **96p** and **96q** displayed the most potent anticancer activities (IC_50_ = 3.11, 2.54 and 5.01, 4.95 µM against A549 and Hela, respectively) as compared to positive control staurosporine with an IC_50_ of 3.05, 2.72 µM against A549 and Hela.

**Scheme 51:**
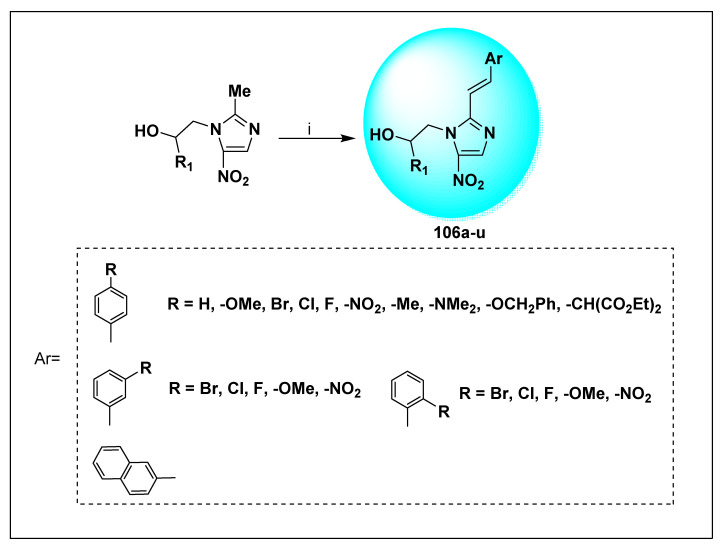
Synthesis 2-styryl-5-nitroimidazole derivatives as antibacterial agents. Reagents and conditions: (**i**) benzaldehydes, NaOMe, DMSO, MeOH, rt, 64–83%.

Duan et al. (2014) designed and synthesized series of novel twenty one 1-(2-hydroxypropyl)-2-styryl-5-nitroimidazole derivatives (**106a–w**) and evaluated them as potentiators of antibacterial agents (Scheme 51) [162]. Their biological activities were evaluated against two Gram-negative bacterial strains: *Escherichia coli* and *Pseudomonas aeruginosa* and two Gram-positive bacterial strains: *Bacillus thuringiensis* and *Bacillus subtilis* by MTT method as potential FabH inhibitor. Among the synthesized analogs, 1-(2-hydroxypropyl)-2-*p*-nitrostyryl-5-nitroimidazole derivative has shown potent activity against *E. coli* FabH.

**Scheme 52:**
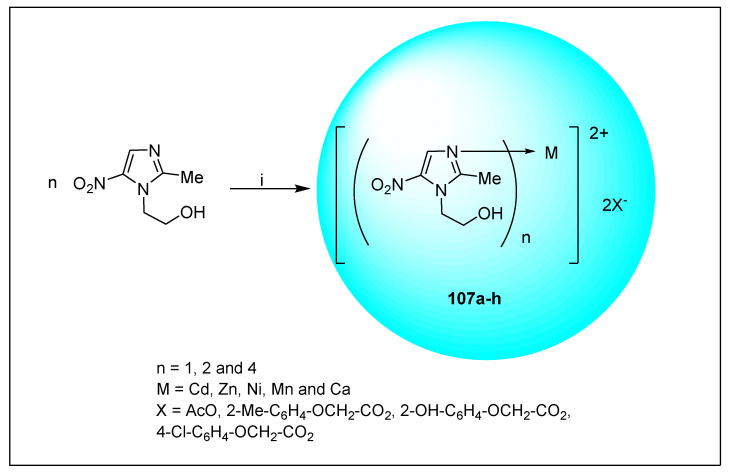
Synthesis complexes of 1-(2-hydroxyethyl)-2-methyl-5-nitroimidazoles. Reagents and conditions: (**i**) MX_2_, MeOH, 2O °C, 10–20 min, 99%.

Adamovich et al., in the year 2014, performed the reaction of 1-(2-hydroxyethyl)-2-methyl-5-nitroimidazole (metronidazole) with salts of arylchalcogenylacetic acids, producing novel and physiologically active metal complexes (**107a–h**) (Scheme 52) [163].

**Scheme 53:**
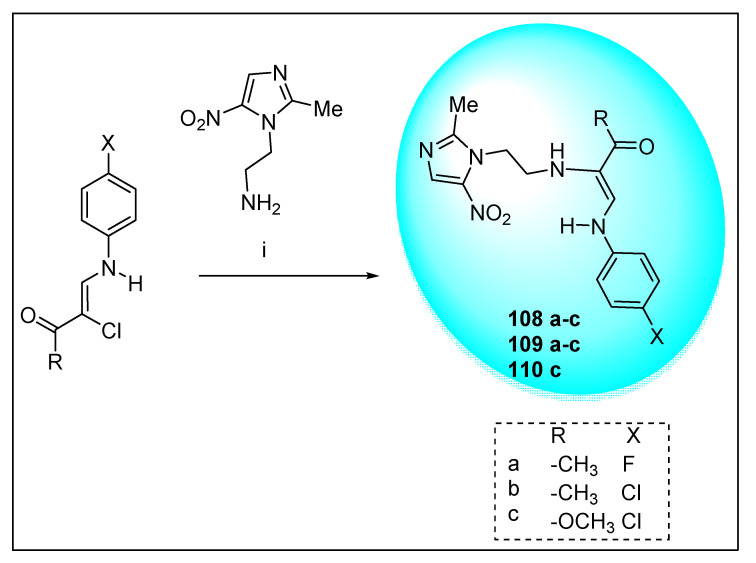
Synthesis of amidrazo-5-nitroimidazole derivatives as antibacterial agents. Reagents and conditions: (**i**) Et_3_N/EtOH/0 °C to rt, 46% and 37%.

Saadeh et al. (2015) synthesized series of new amidrazones (**109a–b**) as shown in Scheme 53. These amidrazones were then evaluated for antitumor, antibacterial, and antiparasitic activities [164]. Compounds **108c** and **109c** displayed strong anticancer activity against all tested cancer cell lines. In addition, compounds **108a** and **109a** displayed stronger antimicrobial potency against microaerophilic bacteria than metronidazole. The compounds **109a**, **110c**, and **109c** exhibited good antigiardial activity better than metronidazole. Compounds **109a**, **109b**, and **109c** also exhibited antigiardial activity as well as antitrichomonal activity.

**Scheme 54:**
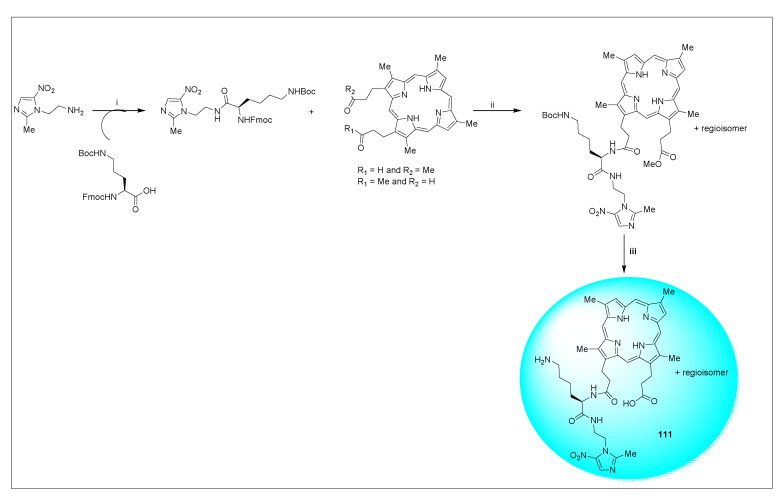
Synthesis of porphyrin-nitroimidazole derivatives as antibiotics. Reagents and conditions: (**i**) HBTU, DIPEA, N_2_, 3 h, and then HOBt, 80%; (**ii**) DBU, DCM, HOBt, HBTU, DIPEA, 90%; (**iii**) Method 1: LiOH, MeOH, THF, H_2_O then TFA, DCM, N_2_, 3 h, 69%, Method 2: TFA, DCM, N_2_, 3 h then LiOH, MeOH, THF, H_2_O, 65%.

Dingsdag et al. in the year 2015 effectively synthesized the “Trojan horse” ester and amide-linked deuterporphyrin-nitroimidazole (DPIX-Nim) adducts to inhibit P. gingivalis (periodontal pathogen) growth. *L*-amino acids were then incorporated into adducts as linkers to improve uptake (Scheme 54). Ten 13- and 17-propionic amide regio-isomers of L-amino acid-linked deuterporphyrin-nitroimidazole adducts were synthesized using a peptide-coupling approach [165].

**Scheme 55:**
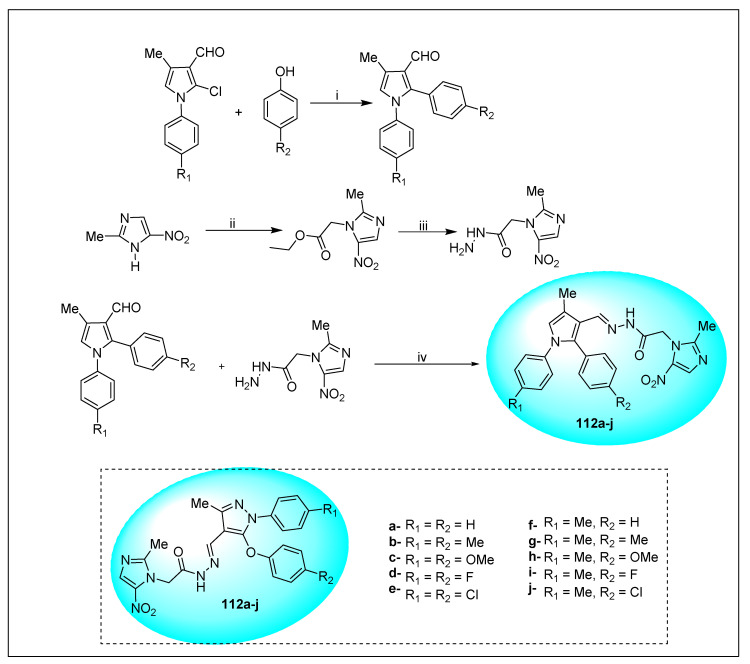
Synthesis of pyrazolo-5-nitroimidazole derivatives as antibacterial agents. Reagents and conditions: (**i**) K_2_CO_3_, DMF (yield not reported); (**ii**) NH_2_-NH_2_.H_2_O, MeOH (yield not reported); (**iii**) ClCH_2_CO_2_Et, K_2_CO_3_, MeCOMe (yield not reported); (**iv**) Ni(NO_3_)_2_.6H_2_O, EtOH, rt, 75–88%.

Zhang et al. (2015) designed, synthesized, and evaluated new Schiff’s base derivatives (**112a**–**j**) by reaction between 5-aryloxypyrazole-4-carbaldehydes and 2-(2-methyl-5-nitro-1*H*-imidazol-1-*yl*)acetohydrazide (Scheme 55). All the synthesized compounds were tested for antibacterial properties and *E. coli* FabH inhibitors. The results revealed that most of the compounds have shown effective antibacterial properties and inhibition of *E. coli* FabH [166].

**Scheme 56:**
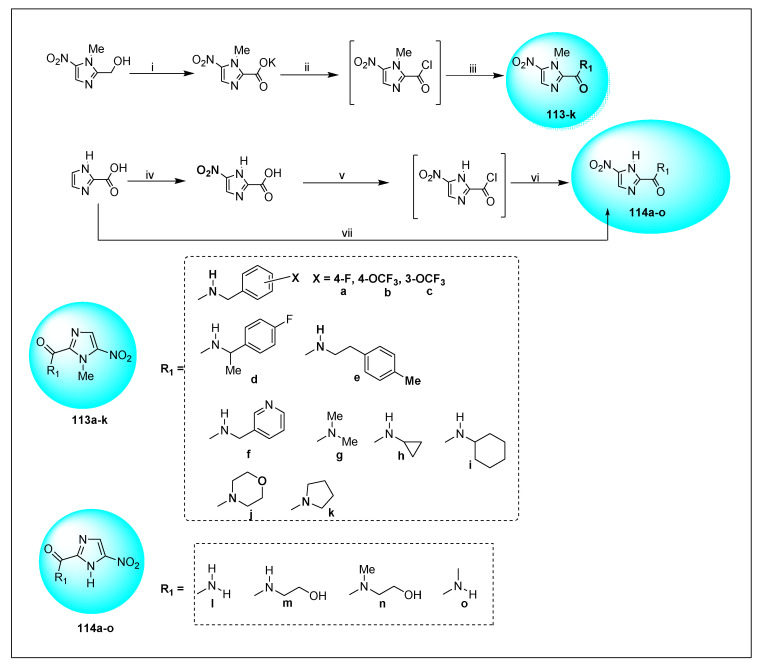
Synthesis of nitroimidazole carboxamide derivatives as antiparasitic agents. Reagents and conditions: (**i**) KMnO_4_, MeCOMe, −5 °C-rt, 85%; (**ii**) oxalyl chloride, cat. DMF, DCM, 0 °C-rt (yield not reported); (**iii**) amine, TEA, DCM, 0 °C-rt, 19-58%; (**iv**) HNO_3_, H_2_SO_4_, 80 °C, 54%; (**v**) oxalyl chloride, cat. DMF, DCM, 0 °C-rt; (**vi**) amine, TEA, DCM, 0 °C-rt, 12–93%; (**vii**) amine, PyBOP, DIPEA, 6–75%.

Jarrad et al. in 2016 re-examined “old” nitroimidazoles and developed new-generation derivatives (**113a**–**k**), (**114a**–**o**) (Scheme 56) [167]. Thirty-three novel nitroimidazole carboxamides were synthesized and evaluated for activity against *G. lamblia* and *E. histolytica*. Most of the new compounds displayed potent activity against *G. lamblia* strains, including metronidazole-resistant strains of *G. lamblia* (EC_50_ = 0.1–2.5 μM cf. Metronidazole EC_50_ = 6.1–18 μM). The other compounds have shown improved activity against *E. histolytica* (EC_50_ = 1.7–5.1 μM cf. Metronidazole EC5_0_ = 5.0 μM), potent activity against *Trichomonas vaginalis* (EC_50_ = 0.6–1.4 μM cf. metronidazole EC_50_ = 0.8 μM) and moderate activity against the intestinal bacterial pathogen *Clostridium difficile* (0.5–2 μg/mL, cf. metronidazole = 0.5 μg/mL). The new compounds showed lower toxicity against mammalian kidney and liver cells (CC_50_ > 100 μM).

**Scheme 57:**
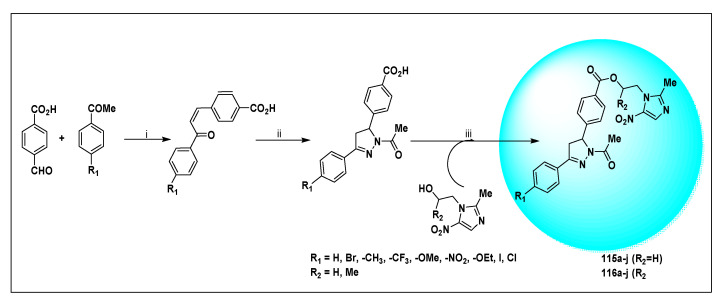
Synthesis of pyrazolylnitroimidazole derivatives as anticancer agents. Reagents and conditions: (**i**) EtOH, NaOH/H_2_O, rt, 8 h (yield not reported); (**ii**) CH_3_COOH, hydrazine hydrate, reflux, 12 h (yield not reported); (**iii**) DCM, EDC, DMAP, reflux, 12 h, 58–72%.

Tao et al. (2016) designed a series of novel pyrazole-nitroimidazole derivatives (**115a**–**j**), and these compounds were tested for EGFR/HER-2 tyrosine kinase inhibitory activity and anti-proliferative properties against cancer cell lines (MCF-7, Hela, HepG2, B16-F10) (Scheme 57). In this study, most of the synthesized compounds exhibited potential anti-proliferation activity, with the IC_50_ values ranging from 0.13 µM to 128.06 µM in all four tested tumor cell lines. [168] The compound **115c** (R_1_ = CF_3_, R_2_ = H) has shown potent inhibitory activity against EGFR/HER-2 tyrosine kinase with IC_50_ values of 0.26 µM/0.51 µM, respectively.

**Scheme 58:**
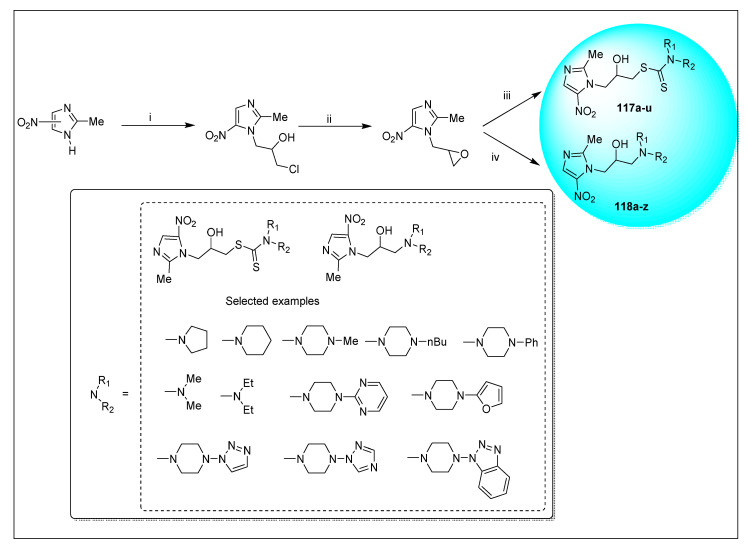
Synthesis of 2-Methyl-4/5-nitroimidazole derivatives as antibacterial agents. Reagents and conditions: (**i**) (±)-Epichlorohydrin, AlCl_3_, EtOAc, 0 °C-rt, 24–26 h, 79%; (**ii**) Aq. NaOH, DCM, 2 h, 91%; (**iii**) substituted-1-carbodithioate, H_2_O:acetone, 0 °C, 2–4 h, 68-91%; (**iv**) substituted amines, MeCN, 80 °C, overnight, 40–85%.

Mandalapu et al. (2016) synthesized a library of sixty 2-methyl-4/5-nitroimidazole derivatives (**117a**–**u**), (**118a**–**z**), and evaluated these compounds against drug-susceptible and resistant *Trichomonas vaginalis* (Scheme 58). All the molecules except for two were found to be active against both susceptible and resistant strains with MICs ranging from 8.55–336.70 μM and 28.80–1445.08 μM, respectively. Most of the compounds showed better activity than the standard metronidazole. The potent compounds were also found to be safe against human cervical HeLa cells with a good selectivity index [115].

**Scheme 59:**
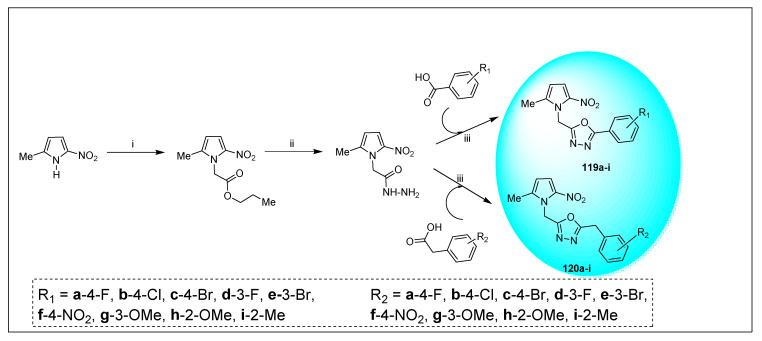
Synthesis of secnidazole analogs as antibacterial agents. Reagents and conditions: (**i**) ClCH_2_CO_2_Et, K_2_CO_3_, MeCOMe (**ii**) H_2_N-NH_2_.H_2_O, MeOH (**iii**) POCl_3_, reflux (yields not reported).

Li et al. in 2012 designed and synthesized a series of secnidazole analogs (**119a-i** and **120a–i**) based on the oxadiazole scaffold. These compounds were tested for antibacterial activities against *Escherichia coli*, *Pseudomonas aeruginosa*, *Bacillus subtilis*, and *Staphylococcus aureus* (Scheme 59) [94]. These new nitroimidazole derivatives had shown strong antibacterial activities. The compounds 2-(2-methoxyphenyl)-5-((2-methyl-5-nitro-1*H*-imidazol-1-yl)methyl)-1,3,4-oxadiazole (**119h**) with MIC of 1.56–3.13 μg/mL against the tested bacterial strains and 2-((2-methyl-5-nitro-1H-imidazol-1-yl)methyl)-5-(2-methylbenzyl)-1,3,4-oxadiazole (**119i**) with MIC of 1.56–6.25 μg/mL were the most potent inhibitors of *Escherichia coli* FabH.

**Scheme 60:**
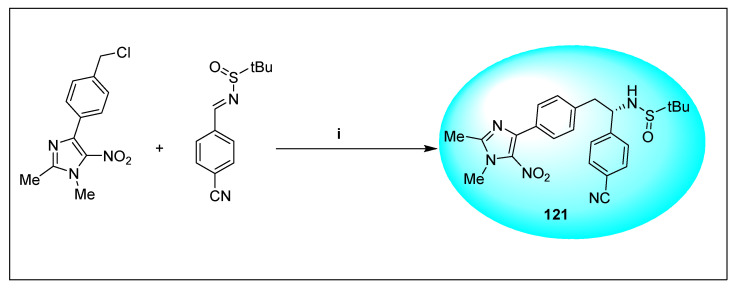
Synthesis of 5-nitroimidazole-based enantiopure amides as antibacterial agents. Reagents and conditions: (**i**) TDAE, DMF, −20 °C to rt, 76%.

Spitz et al. (2016) developed the metal-free synthesis of 5-nitroimidazole-based enantiopure amides (**121**). The new method having mild reaction conditions and tolerance of various substitutions makes this approach effective for the construction of pharmacologically active compounds (Scheme 60) [169].

### 2.4. Scaffold

#### 2.4.1. CGI-17341 **17** and Its Derivatives

**Figure 21:**
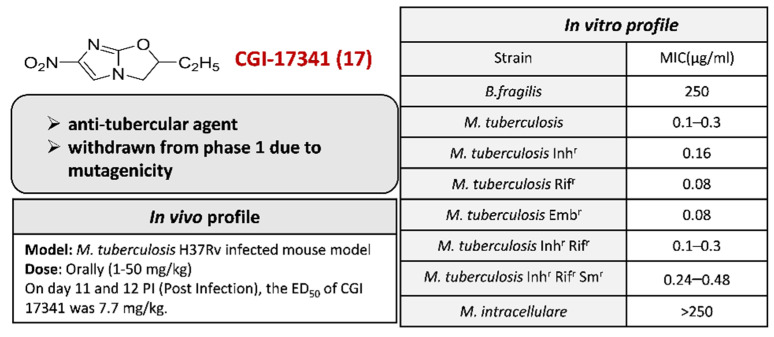
Activity profile of CGI-17341 **17** [16].

CGI-17341 **17** was the first molecule belonging to the bicyclic nitroimidazole analog, developed by Hindustan Ciba-Geigy in 1989, that was active against drug-susceptible as well as MDR TB. CGI-17341 showed a minimum inhibitory concentration (MIC) of 0.06 µg/mL in vitro assay. In addition, it showed excellent efficacy in an in vivo mice model (Figure 21) [16]. It was the first molecule from the nitroimidazole class to enter the clinical trials against tuberculosis, but later on, it was halted due to its mutagenic behavior from phase I clinical trials.

**Scheme 61:**
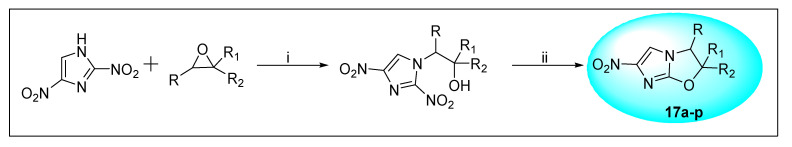
Synthesis of CGI-17341 **17** as anti-tubercular agent. Reagents and conditions: (**i**) sodium acetate, EtOH, 70 °C, 12 h (yield not reported); (**ii**) NaH, dioxane, 50 °C, 1 h, 9–35%.

Nagrajan et al. (1989) discovered CGI-17341 **17b** as anti-tubercular agents (Scheme 61). In this, the authors had synthesized a series of fused bicyclic compounds bearing aliphatic moiety. Among the synthesized compounds, most of the compounds had shown MIC values less than 1 μg/mL (mostly in the range of 0.95–0.0037 μg/mL). Among them, CGI-17341 **17b** had shown a better in vitro as well as in vivo profile (Table 4). The synthetic strategy involved the nucleophilic epoxide opening with 2,4-dinitroimidazole followed by the base catalyzed cyclisation [17].

#### 2.4.2. Delamanid **18** and Its Derivatives

**Figure 22:**
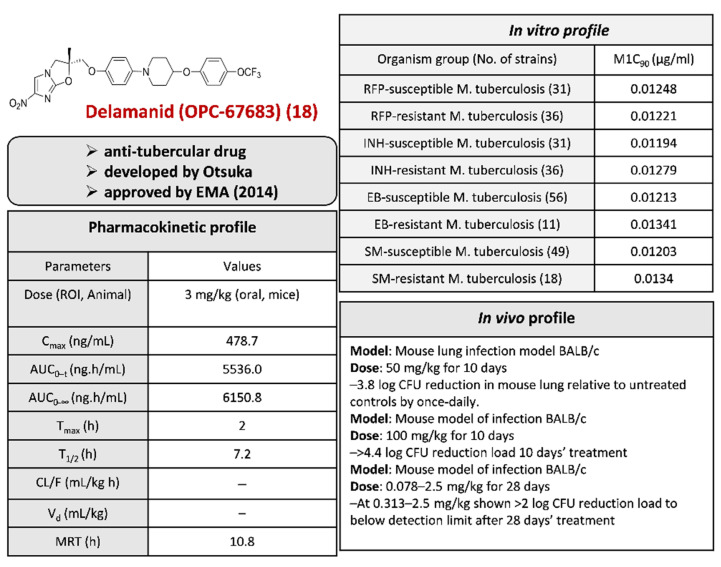
Activity profile of Delamanid **18** [170,171].

Delamanid **18** is an anti-tuberculosis agent, belonging to the class of nitro-dihydroimidazooxazole, developed and marketed by Otsuka Pharmaceutical Co. Ltd. (Tokyo, Japan, Figure 22). It has a dual mechanism of action by inhibiting mycolic acid biosynthesis and is an NO donor. It is used in the treatment of multidrug-resistant (MDR-TB) and extensively drug-resistant tuberculosis (XDR-TB) in a combination regimen. Delamanid **18** has the highest potency in vitro testing against sensitive and resistant strains, i.e., 0.006 µg/mL, including Rif^R^, INH^R^ and, non-replicating phase of bacteria **[170,171,172]**. Delamanid **18** has also shown good plasma exposure in a pharmacokinetic study in all three species, i.e., mice, rats, and dogs, and has in vivo efficacy in both acute and chronic mice models of MTB infection. The clinical studies also reveal that Delamanid **18** has better potency against multidrug-resistant tuberculosis. It was approved by the European Union of Medicine (EMA) in 2014 and is marketed under the trade name Deltyba as oral tablet.

**Scheme 62:**
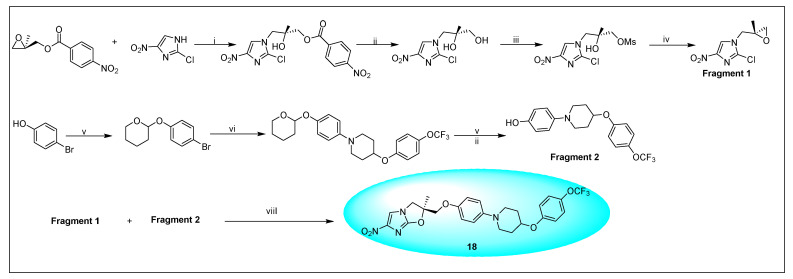
Synthesis of OPC-67683 **18** as anti-tubercular agent (Sasaki et al.). Reagents and conditions: (**i**) Et_3_N, AcOEt, 60–65 °C, 6 h, 87%; (**ii**) K_2_CO_3_, MeOH, rt, 2 h, 97%; (**iii**) MsCl, C_5_H_5_N, <15 °C, 2 h (yield not reported); (**iv**) DBU, AcOEt, rt, 2 h, 75%; (**v**) 3,4-dihydro-2*H*-pyran Et_3_N, EtOH, 60-65 °C, 6 h (yield not reported); (**vi**) 4-(4-(trifluoromethoxy)phenoxy)piperidinePd_2_(dba)_3_, rac-BINAP, Cs_2_CO_3_, toluene, 70 °C, 6 h (yield not reported); (**vii**) pyridinium *p*-toluenesulfonate, EtOH, 70 °C, 24 h (yield not reported); (**vii**) NaH, DMF, 50 °C, 2 h, 48%.

The first synthesis of Delamanid **18** was performed by Sasaki et al. in 2006, which involved the preparation of two key fragments (fragments 1 and 2) (Scheme 62). The synthesis of fragment 1 involved the nucleophilic attack of imidazole on epoxide in the presence of base trimethylamine, followed by deprotection, mesylation and epoxide formation. Simultaneously, 2-(4-bromophenoxy)tetrahydropyran was reacted with 4-(4-trifluoromethoxy)phenoxypiperidine to produce the intermediate, which upon deprotection with pyridinium *p*-toluenesulfonate in ethanol, produced the desired fragment 2. Then, the final step involved coupling and cyclisation of both fragments in the presence of sodium hydride, to give Delamanid **18** [172].

**Scheme 63:**
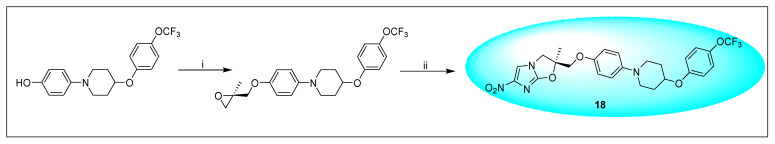
Synthesis of OPC-67683 **18** as anti-tubercular agent (Tsubouchi et al.). Reagents and conditions: (**i**) R-(2-methyloxiran-2-yl)methyl-4-nitrobenzenesulfonate, NaO*t*Bu, acetone, 93%; (**ii**) 2-bromo-5-nitroimidazole, NaOAc, 110 °C, 2 h, and then DMF, −5 °C, NaO*t*Bu, 3 h, and then H_2_O, EtOAc, 60 °C, 71%.

In 2008, Hidetsugu et al. from Otsuka Pharmaceuticals developed an alternative approach for Delamanid **18**, wherein an epoxy containing fragment was synthesized by treating 4-(4-(trifluoromethoxy)phenoxy)piperidin-1-yl)phenol with (2-methyloxiran-2-yl)methyl 4-nitrobenzenesulfonate in the presence of sodium *tert*-butoxide as base. The intermediate obtained then underwent nucleophilic reaction with 2-bromo-5-nitroimidazole followed by cyclization-furnished Delamanid **18** in 71% yield. (Scheme 63) [173].

**Scheme 64:**
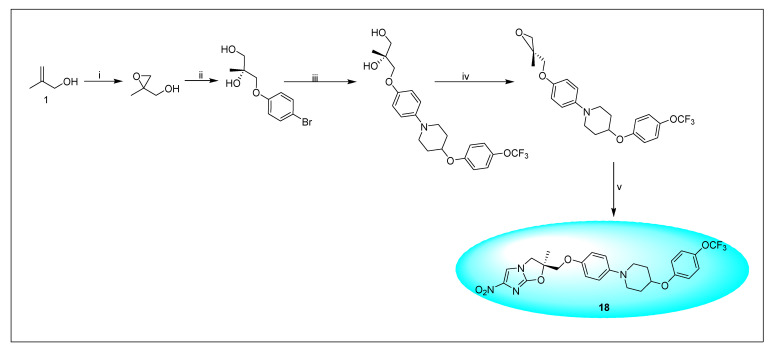
Synthesis of OPC-67683 **18** as anti-tubercular agent (Yamamoto et al.). Reagents and conditions: (**i**). *D*-(-)-diisopropyl tartrate, toluene, −18 °C; Ti(OPr-*i*)_4_, cumene hydroperoxide, -18 °C, DMSO, rt; (**ii**) 4-bromo phenol, toluene, 25% aq. NaOH, 40 °C, 9 h; (**iii**) 4-(4-(trifluoromethoxy)phenoxy)piperidine, Pd_2_(dba)_3_, NaO*t*Bu, *t*BuXPhos, toluene, 70 °C, 6 h; (**iv**) E_t3_N, EtOAc, MeSO_2_Cl; (**v**) K_2_CO_3_, MeOH, 0 °C to rt; (**v**) 2-bromo-4-nitro-1*H*-imidazole, AcONa, *t*-BuOAc, 3.5 h, 100 °C, and then MeOH, aq. NaOH, 42% (overall yield).

Akihiro et al. (in 2011) from Otsuka pharmaceuticals developed the new synthetic strategy (Scheme 64) [174]. In this method, *β*-methallyl alcohol was converted to (*S*)-2-methylglycidol via sharpless epoxidation, which was then treated with bromophenol to produce diol and then to epoxide. Then, the final step involved nucleophilic addition of an imidazole ring to epoxide, followed by ring closure to furnish **18** with high enantiomeric excess (99.4% *ee*).

**Scheme 65:**
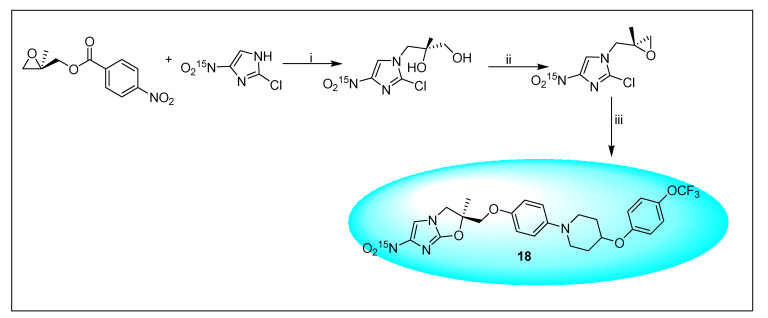
Synthesis of OPC-67683 **18** as anti-tubercular agent (Graham et al.). Reagents and conditions: (**i**) Et_3_N, EtOAc, 60 °C, 6 h, and then K_2_CO_3_, MeOH, rt, 12 h (yield not reported); (**ii**) MsCl, Pyridine, <15 °C, 2 h, and then DBU, EtOAc, rt, 2 h (yield not reported); (**iii**) 4-(4-(trifluoromethoxy)phenoxy)piperidine, NaH, DMF, 50 °C (yield not reported).

In 2015, Timmins et al. patented another route for the synthesis of N^15^-labeled Delamanid **18** (Scheme 65). In this approach, epoxide fragment and 2-chloro-5-nitroimidazole were reacted in the presence of base triethylamine to produce intermediate, which upon further treatment with methanol, produced the diol product. The diol was mesylated followed by cyclization-produced epoxide, and finally this epoxide was coupled with 4-(4-(trifluoromethoxy)phenoxy)piperidine to furnish the objective N^15^-labeled Delamanid **18** [175].

**Scheme 66:**
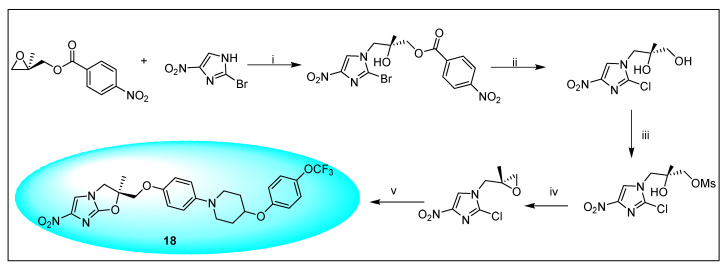
Synthesis of Delamanid **18** (Fairlamb et al.). Reagents and conditions: (**i**) DIPEA, AcOEt, 60–65 °C, 20 h (yield not reported); (**ii**) K_2_CO_3_, MeOH, rt, 16 h (yield not reported); (**iii**) MsCl, C_5_H_5_N, DCM, 0 °C-rt 16 h (yield not reported); (**iv**) DBU, AcOEt, rt, 16 h (yield not reported); (**v**) 4-(4-(trifluoromethoxy)phenoxy)piperidine, NaH, DMF, 50 °C, 1.5–4 h (yield not reported).

Patterson et al. synthesized Delamanid **18** by using 2-bromo-4-nitroimidazole instead of chloro-imidazole used by the Sasaki et al. in the same route as shown in Scheme 66 [176].

**Scheme 67:**
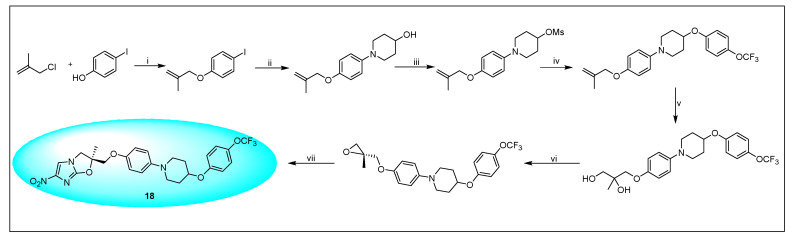
Synthesis of Delamanid **18** (Sharma et al.) [177]. Reagents and conditions: (**i**) K_2_CO_3_, DMF, 60 °C, 97%; (**ii**) 4-hydroxypiperidine, CuI, L-Proline, DMF, 10–24 h, 81%, (**iii**) mesyl chloride, Et_3_N, DCM, 0 °C to rt, 93%; (**iv**) 4-trifluoromethoxyphenol, PPh_3_, DEAD, THF, rt, 24 h, 70%; (**v**) AD mix-β, tert-butanol: water (1:1), 0 °C, 6h, 89%; (**vi**) mesyl chloride, Et_3_N, DCM, 0 °C to rt, and then, DBU, rt, 2 h, 82%; (**vii**) 2-bromo-4-nitroimidazole, DIPEA, 115 °C, and then, Cs_2_CO_3,_ DMF, 50 °C, 2 h, 70%.

Recently, our group (2020) developed a concise and sequential route for the synthesis of Delamanid **18** (Scheme 67). In this method, 2-methylallyl chloride 1 (or 3-chloro-2-methylprop-1-ene) was taken as the starting material, which underwent sequential reaction cascades viz. allylation, selective *N*-arylation, Mitsunobu etherification, Sharpless asymmetric dihydroxylation, and epoxidation to furnish chiral epoxide as the key starting material. The coupling of chiral epoxide with 2-bromo-4-nitroimidazole furnished the required product **18** with an overall yield of 27% [177].

#### 2.4.3. Pretonamid (PA-824) (**19**)

**Figure 23:**
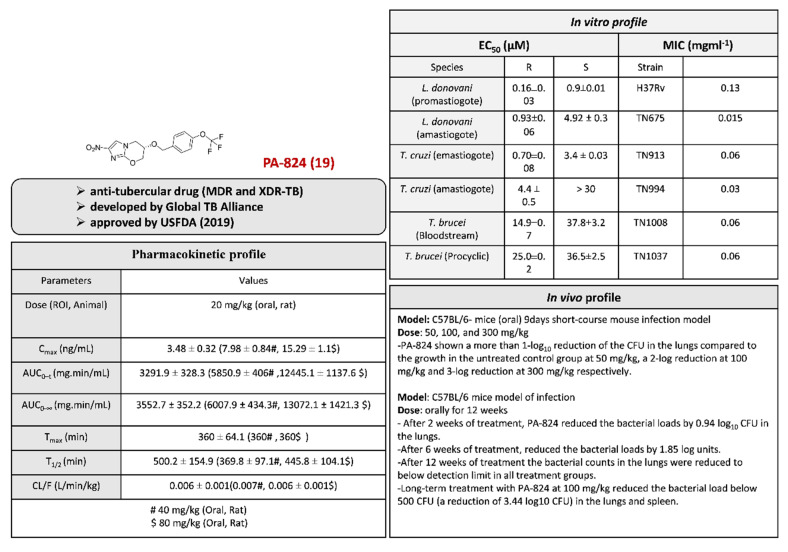
Activity profile of PA-824 **19** [26,178,179,181].

Pretomanid, also known as PA-824 **19** (Figure 23), belongs to nitroimidazooxazine. Pretomanid was recently approved (2019) for the MDR and XDR *Mycobacterium tuberculosis*. Inspired from the promising activity profile of CGI-17341 **17**, Pathogenesis Corporation (Novartis) had made an extensive effort and came up with the discovery of pretomanid (PA-824) with improved potency and without any mutagenicity. Pretomanid (PA-824) has promising bactericidal activity against replicating and non-replicating phases of MTB as well as potent activity against MDR-TB along with oral bioavailability and excellent in vivo activity [178,179]. Pretomanid **19** has also shown an excellent activity profile against various strains of leishmaniasis and was provided another opportunity for its development as an anti-leishmanial drug [180].

**Scheme 68:**
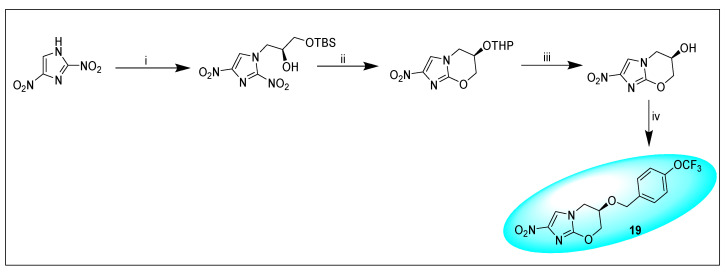
Synthesis of PA-824 **19** (Baker et al.). Reagents and conditions: (**i**) *tert*-butyldimethyl(oxiran-2-ylmethoxy)silane, DIPEA, toluene, 70 °C (yield not reported); (**ii**) DHP, *p*-TsOH, TBAF, THF (yield not reported); (**iii**) CH_3_COOH, THF (yield not reported); (**iv**) 4-trifluoromethoxy benzyl bromide, NaH, DMF, 70%.

Baker et al. (2000) developed the synthetic route for the synthesis of PA-824 **19**. In this, the authors started the synthesis of a targeted molecule from 2,4-dinitroimidazole (Scheme 68) [182]. The first step involved the epoxide opening of *tert*-butyldimethyl(oxiran-2-ylmethoxy)silane with 2,4-dinitroimidazole, which led to the formation of intermediate (S)-1-((tert-butyldimethylsilyl)oxy)-3-(2,4-dinitro-1H-imidazol-1-yl)propan-2-ol, which subsequently underwent protection of secondary alcohol with dihydropyran followed by deprotection of *tert*-butyldimethylsilyl group and cyclisation leading to the formation of an oxazine ring, i.e., (R)-2-nitro-6,7-dihydro-5H-imidazo[2,1-*b*][1,3]oxazin-6-ol, which was then coupled with 4-trifluoromethoxy benzyl bromide to produce the target molecule **19**.

**Scheme 69:**
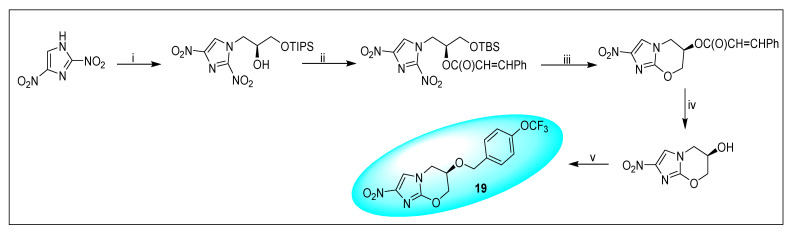
Synthesis of PA-824 **19** (Orita, Akihiro et al.). Reagents and conditions: (**i**) Glycidyl silyl ether, EtOH, 70 °C, 85%; (**ii**) Cinnamic acid, DCC, DMAP, toluene, rt, 92%; (**iii**) TBAF, THF, rt, 1 h, 91%; (**iv**) Ti(O-*i*-Pr)_4_, MeOH, reflux, 24 h, 93%; (**v**) 1-(bromomethyl)-4-(trifluoromethoxy)benzene, NaH, DMF, rt, 70%.

Orita et al. (2007) developed another process with improved yield for the synthesis of PA-824 **19** [183]. The yield was improved from 53% to 80%. The process involved the ring opening of glycidyl ether with 2,4-dinitroimidazole followed by the protection of primary hydroxyl group with triisopropylsilane (better protecting group compared to TBS) under the solventless conditions. Another advantage of this route was the use of cinnamyl ester instead of THP, which increased the yield of cyclic intermediate to 66%. The overall yield was increased nearly 2.5 times as compared with the previous method (Scheme 69).

**Scheme 70:**
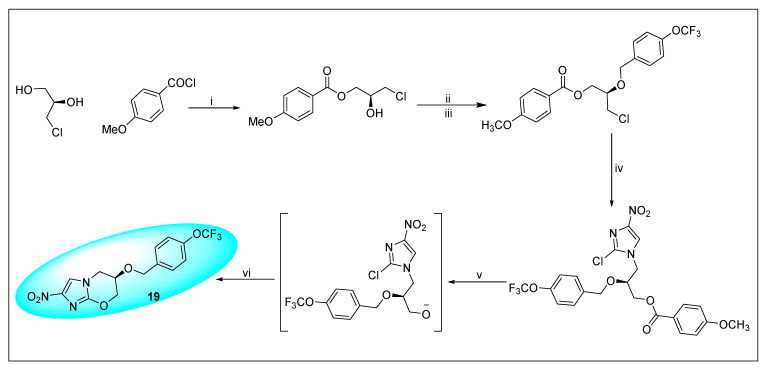
Synthesis of PA-824 **19** (Erik J. Sorensen et al.). Reagents and conditions: (**i**) imidazole, CH_2_Cl_2_, 0 °C to rt, 78–82%; (**ii**) (4-(trifluoromethoxy)phenyl)methanol, NaH, Cl_3_CCN, TBME, heptane, 0 °C to rt, 98%; (**iii**) TfOH, CH_2_Cl_2_, 0 °C to rt, 80%; (**iv**) 2-chloro-5-nitroimidazole, K_2_CO_3_, NaI, DMF, 120 °C, 40–50%; (**v**) KOH, MeOH, 0 °C to rt; (**vi**) 0 °C to rt, 1 h, 60–70%.

Marsini et al. (2010) developed a concise and convergent synthesis of PA-824 **19**. The synthetic route involved in this process was safest and practical. The starting material used in the previous method was explosive (Scheme 70) [184]. In this protocol, the key strategic concern was based on the straight, convergent coupling of a safer starting material, i.e., 2-chloro-4-nitroimidazole with an appropriately functionalized glycidol derivative. The key steps involved N-alkylation of 2-chloro-4-nitroimidazole with chloro-intermediate in the presence of sodium iodide and potassium carbonate, which produced the key intermediate followed by saponification of benzoate ester at 0 °C to room temperature, which involved the spontaneous anionic cyclization–elimination to produce the product with >99.9% *ee.*

**Scheme 71:**
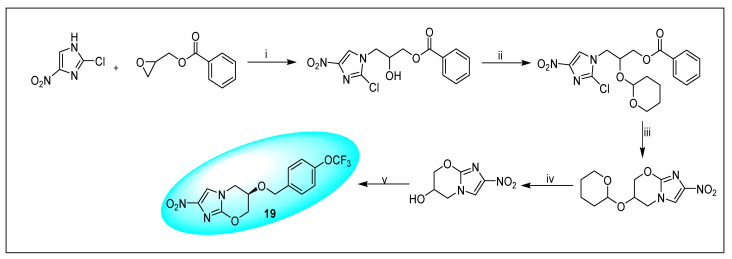
Synthesis of PA-824 **19** (Rahul R. et al.). Reagents and conditions: (**i**) EtOAc, Et_3_N, rt, 65%; (**ii**) 3,4-dihydro-2*H*-pyran, Pyridine, *p*-MePhSO_3_H, 98%; (**iii**) KOH, MeOH, 85%; (**iv**) *p*-methylsulfonic acid, MeOH, rt, 70%; (**v**) 1-(bromomethyl)-4-(trifluoromethoxy)benzene, *t*-BuONa, DMF, 70%.

Recently, Rao et al. (2018) described the synthesis of PA-824 **19** in a similar way to that of the strategy by Baker et al. (Scheme 71). The only difference was the use of starting material 2-chloro-4-nitroimidazole instead of 2,4-dinitroimidazole. The current developed strategy was amenable to the bulk scale synthesis of the drug candidate [185].

#### 2.4.4. VL-2098 (**20**) and Its Derivatives

**Figure 24:**
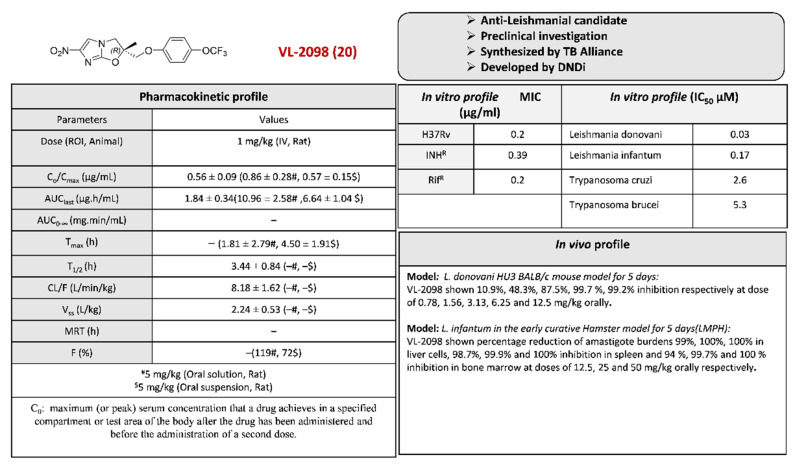
Activity profile of VL-2098 **20** [186,187].

VL-2098 **20** belongs to the class of 4-nitroimidazooxazole series, synthesized by TB Alliance as an anti-tubercular agent but later screened by the Drugs for Neglected Diseases Initiative and was identified as a potent anti-leishmanial compound (Figure 24). Initially, a series of nitroimidazole-based compounds were selected, and among them, VL-2098 **20** was found to be a potent and safe molecule and was therefore selected for in-depth evaluation of its efficacy, pharmacokinetic and early safety profile. VL-2098 **20** has shown potent activity against *Leishmanial* species as well as tuberculosis and *trypanosomal* species in in vitro assays. It has also shown an excellent in vivo profile against acute and chronic visceral leishmaniasis animal models after oral dosing. However, in longer duration studies, a narrow therapeutic window halted its further development, which is now being actively pursued for a new generation of better analogs [186].

**Scheme 72:**
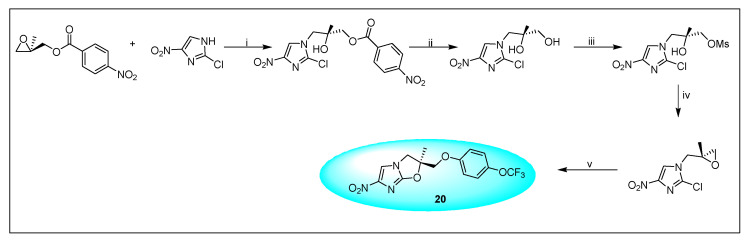
Synthesis of VL-2098 **20** (Sasaki et al.). Reagents and conditions: (**i**) Et_3_N, AcOEt, 60–65 °C, 6 h, 87%; (**ii**) K_2_CO_3_, MeOH, rt, 2 h, 97%; (**iii**) MsCl, C_5_H_5_N, <15 °C, 2 h (yield not reported); (**iv**) DBU, AcOEt, rt, 2 h, 75%; (**v**) 4-(trifluoromethoxy)phenol, NaH, DMF, 50 °C, 2 h (yield not reported).

Sasaki et al. (2006) synthesized VL-2098 **20** [172]. In this, the authors started the synthesis by the reaction of 5-nitroimidazole with (2-methyloxiran-2-yl)methyl 4-nitrobenzoate to obtain the intermediate, which underwent deprotection to produce diol. Then, the next step involved the protection of primary alcohol with mesyl chloride, epoxidation, and nucleophilic opening with aromatic phenol furnishing the desired compound VL-2098 (**20**) (Scheme 72).

**Scheme 73:**
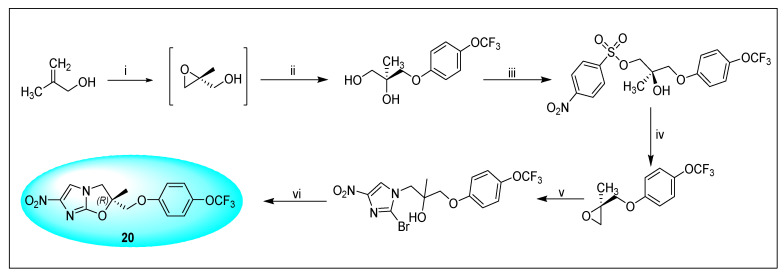
Synthesis of VL-2098 **20** (Satam et al.). Reagents and conditions: (**i**) Ti(O-*i*Pr)_4_, *D*(-)DIPT, TBHP, DCM, −25 °C to −30 °C (yield not reported); (**ii**) K_2_CO_3_, 4-trifluromethoxyphenol, MeOH, rt −60 °C, 43%; (**iii**) *p*-nitrobenzenesulfonyl chloride, Et_3_N, DCM, 0 to −5 °C (yield not reported); (**iv**) aqueous NaOH solution, 0 to 15 °C, 92%; (**v**) 2-bromo-4-nitroimidazole, DIPEA, 110 °C −115 °C, 96%; (**vi**) K_2_CO_3,_ DMF, 90 °C, 51%.

Satam et al. (2017) developed a scalable process for the synthesis of VL-2098 **20**. In this, the authors had developed the synthesis of VL-2098 **20** in four steps (Scheme 73) [187]. The target compound synthesis involved: (i) sharpless asymmetric epoxidation of 2-methyl-2-propen-1-ol; followed by (ii) nucleophilic opening of the epoxide ring with 4-trifluoromethoxyphenol; (iii) sulfonylation of diol with *p*-nitrobenzenesulfonyl chloride; and then (iv) reaction with 2-bromo-4-nitroimidazole.

**Scheme 74:**
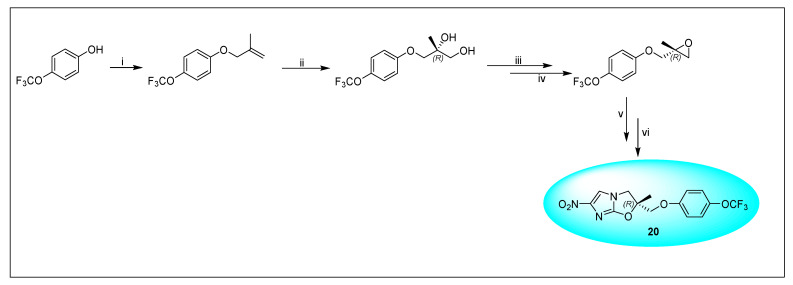
Synthesis of VL-2098 (**20**). Reagents and conditions: (**i**) 2-methylallyl chloride, K_2_CO_3_, DMF, 60–65 °C, 2 h, 97%; (**ii**) AD-mix *α*, *t*-BuOH–H_2_O (1:1), rt, 2 h, 88%; (**iii**) MsCl, Et_3_N, EtOAc, <15 °C-rt, 2 h; (**iv**) DBU (4 mmol), EtOAc, rt, 2 h, 89%; (**v**) DIPEA, 110 °C, 2 h; (**vi**) Cs_2_CO_3_, DMF, 50 °C, 2 h, 48%.

Recently, our group developed another shorter route for the synthesis of VL-2098 (**20**). In this, 2-methylallyl chloride was reacted with 4-trifuoromethoxy phenol under basic conditions to obtain 1-((2-methylallyl)oxy)-4-(trifuoromethoxy)benzene (Scheme 74). The next step involved the Sharpless asymmetric dihydroxylation where 1-((2-methylallyl)oxy)-4-(trifuoromethoxy)benzene was treated with AD mix-*α* to obtain the diol product. The diol was then converted into a key intermediate epoxide via mesylation. The final step involved the coupling between epoxide and 2-bromo-4-nitroimidazole to obtain the desired product VL-2098 (**20**). This new approach has an overall yield of 36% [177].

#### 2.4.5. TBA-354 (**21**) and Its Derivatives

**Figure 25:**
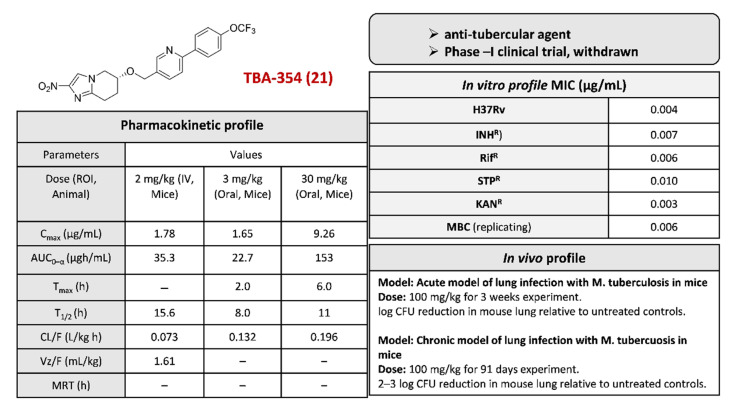
Activity profile of TBA-354 **21** [188].

TBA-354 **21** was the second-generation TB drug candidate belonging to the class of nitroimidazo-oxazine (PA-824) developed by the Global Alliance for TB Drug Development. It is a pyridine-containing biaryl compound with improved promising bioavailability and efficacy against chronic murine tuberculosis (Figure 25). It entered into phase 1 of clinical trials, but based on the observed side effects, was withdrawn [188].

**Scheme 75:**
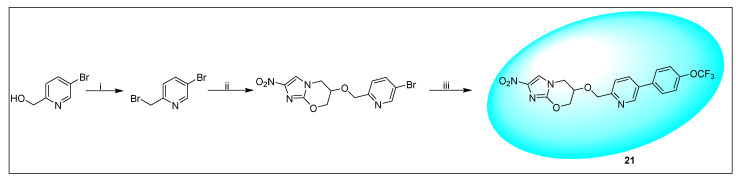
Synthesis of TBA-354 **21**. Reagents and conditions: (**i**) NBS, PPh_3_, CH_2_Cl_2_, 20 °C, 3–3.5 h, 91%; (**ii**) 2-nitro-6,7-dihydro-5*H*-imidazo[2,1-*b*][1,3]oxazin-6-ol, NaH, DMF, 0–20 °C, 2–3 h, 88%; (**iii**) ArB(OH)_2_, toluene, EtOH, K_2_CO_3_, Pd(dppf)Cl_2_ under N_2_, reflux, 20 min −21 h, 70%.

Kmentova et al. in 2010 synthesized new heterocyclic analogs of the potent biphenyl class derived from antitubercular drug PA-824, intending to improve aqueous solubility and maintain high metabolic stability and efficacy. From this strategy, one compound (TBA-354) emerged as a potent new anti-tubercular lead and was taken up for a phase I clinical trial. The synthesis of TBA-354 **21** was made from (5-bromopyridin-2-yl)methanol, which upon reaction with *N*-bromosuccinamide, converted into 5-bromo-2-(bromomethyl)pyridine. This intermediate underwent reaction with 2-nitro-6,7-dihydro-5*H*-imidazo[2,1-*b*][1,3]oxazin-6-ol followed by Suzuki coupling to furnish the desired product **21** (Scheme 75) [189].

#### 2.4.6. Miscellaneous Fused Nitroimidazoles

**Scheme 76:**
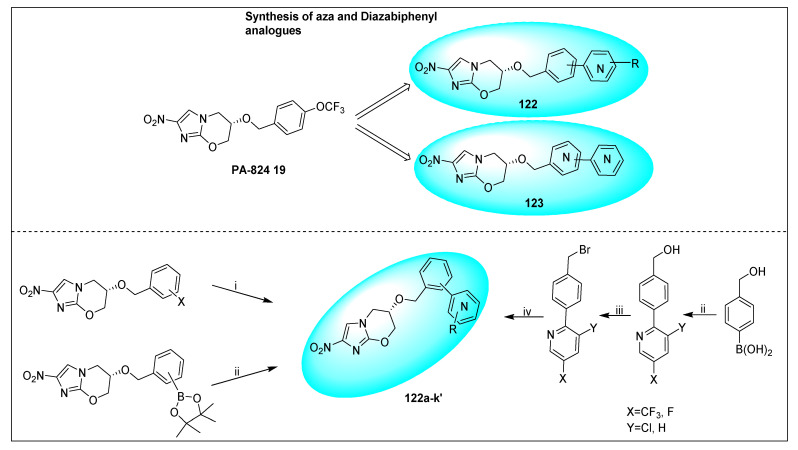
Synthesis of Aza analogs of PA-824 **19**. Reagents and conditions: (**i**) ArB(OH)_2_, toluene, EtOH, KOAc, Pd(dppf)Cl_2_ under N_2_, reflux, 10 min–3 h, 19–97%; (**ii**) substituted halopyridine, toluene, EtOH, K_2_CO_3_, Pd(dppf)Cl_2_ under N_2_, reflux, 0.5–3 h, 50–83%; (**iii**) NBS, PPh_3_, CH_2_Cl_2_, 20 °C, 3–4 h, 87–97%; (**iv**) 2-nitro-6,7-dihydro-5*H*-imidazo[2,1-b][1,3]oxazin-6-ol, NaH, DMF, 0–20 °C, 2.2–2.5 h, 74–84%.

**Scheme 77:**
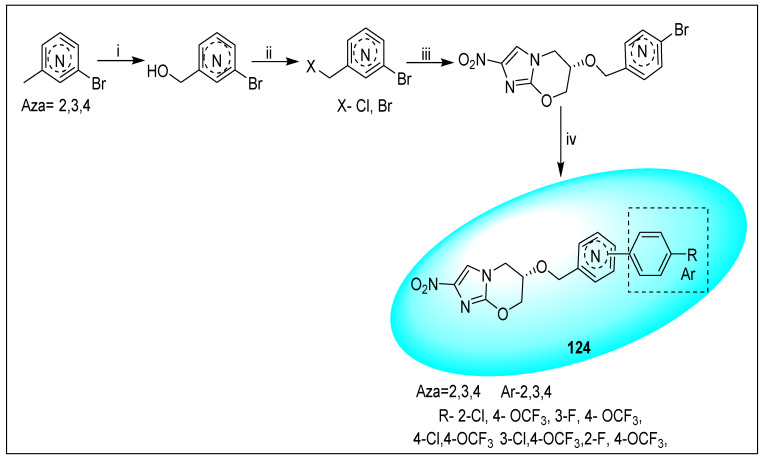
Synthesis of Aza analogs of PA-824 **19**. Reagents and conditions: (**i**) TFAA, 20 °C, 30 min, and then reflux, 30 min, and then aqueous NaHCO_3_, 20 °C, 16 h, 78% (**ii**) SOCl_2_, CHCl_3_, 0–20 °C, 20 h, or reflux, 1 h, 83%; (**iii**) 2-nitro-6,7-dihydro-5*H*-imidazo[2,1-*b*][1,3]oxazin-6-ol, NaH, DMF, −5 to 20 °C, 1–16 h, 65%; (**iv**) ArB(OH)_2_ (or pinacol ester), toluene, EtOH, K_2_CO_3_, Pd(dppf)Cl_2_ under N_2_, reflux, 10 min–6 h, 42–92%.

**Scheme 78:**
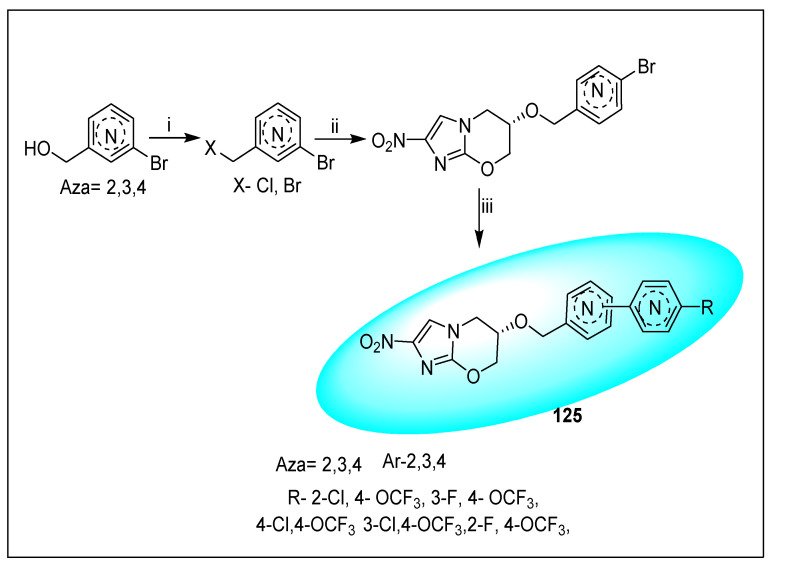
Synthesis of Aza analogs of PA-824 **19**. Reagents and conditions: (**i**) NBS/NCS, PPh_3_, CH_2_Cl_2_, 20 °C, 3–3.5 h, 83%; (**ii**) NaH, DMF, 0–20 °C, 2–3 h, 81%; (**iii**) ArB(OH)_2_ (or pinacol ester), toluene, EtOH, K_2_CO_3_, Pd(dppf)Cl_2_ under N_2_, reflux, 20 min, 21 h, 54–80%.

Kmentova et al. (2010) synthesized and developed the structure–activity relationship for the aza and diazabiphenyl analogs of the antitubercular drug PA-824 **19** (Scheme 76, Scheme 77 and Scheme 78). In this, the authors had developed new heterocyclic analogs of the potent biphenyl class in order to improve the aqueous solubility, metabolic stability and efficacy. The authors had performed the modifications by replacing one or both phenyl groups by pyridine, pyridazine, pyrazine, or pyrimidine in order to reduce lipophilicity. After synthesizing, the compounds were evaluated. In this study, the terminal pyridine or proximal heterocycle compounds showed good potency, better solubility, high metabolic stabilities, and excellent pharmacokinetics. Broadly, these showed that replacement of one of the phenyl groups with pyridine lowered ClogP values by ~1.3 units, whereas replacement with a diaza heterocycle had a more variable effect, with lipophilicity differences ranging from −1.41 (30, 50-pyrimidine) to −2.84 (pyridazine). Additional replacement of the second phenyl group with pyridine further reduced the ClogP values (by ∼0.6–1.1 units), providing particularly hydrophilic analogs [189]. This study has led to the discovery of TBA-354, which is discussed in the previous section.

**Scheme 79:**
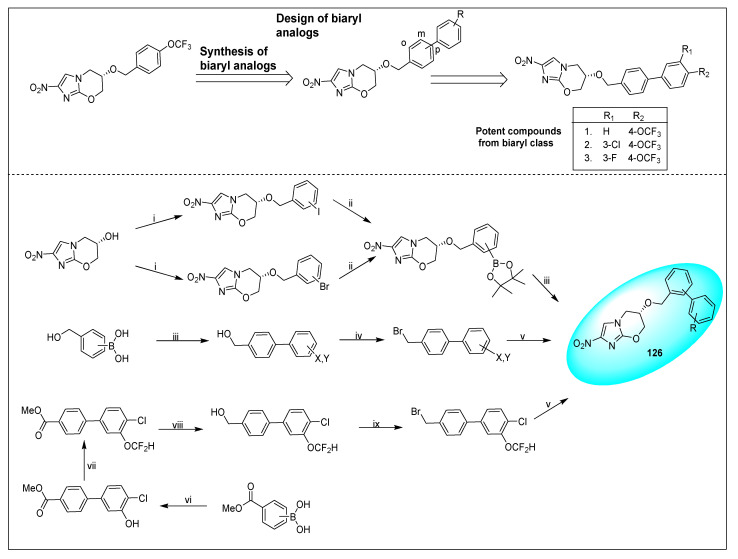
Synthesis of derivatives of PA-824 **19** as anti-tubercular agents. Reagents and conditions: (**i**) iodo/bromo benzylhalides, NaH, DMF, 20 °C, 61–97%; (**ii**) ArB(OH)_2_, toluene, EtOH, KOAc, Pd(dppf)Cl_2_, DMSO, 90 °C, 1 h, 51–61%; (**iii**) halobenzenes, toluene, K_2_CO_3_, Pd(dppf)Cl_2_ under N_2_, reflux, 15–70 min, 51–80%; (**iv**) 33% HBr, acetic acid, 20 °C, 6–11 h, 100%; (**v**) 2-nitro-6,7-dihydro-5*H*-imidazo[2,1-b][1,3]oxazin-6-ol, NaH, DMF, 0–20 °C, 3 h, 70–79%; (**vi**) (5-Br, 2-Cl)PhOH, toluene, EtOH, K_2_CO_3_, Pd(dppf)Cl_2_ under N_2_, reflux, 30 min, 81%; (**vii**) NaOCOCClF_2_, K_2_CO_3_, DMF, 80 °C, 14 h, 37%; (**viii**) LiAlH_4_, Et_2_O, 0–20 °C, 2 h, 100%; (**ix**) PBr_3_, Et_2_O, 0–20 °C, 3 h, 82%.

Palmer et al. (2009) synthesized and developed structure–activity relationship studies for biphenyl analogs of PA-824 **19** (Scheme 79) [190]. In this, the authors had performed the synthesis of biphenyl analogs by coupling the iodobenzyl alcohols with iodides and appropriate boronic acids and then evaluated them against antitubercular activity. The structure–activity relationship of synthesized biphenyl analogs clearly showed that *para*-linked biaryls more active flowed by *meta* then *ortho*-linked *biaryl* analogs under both replicating and non-replicating conditions. Then, most potent analogs were screened for detailed study for the in vivo efficacious study in acute MTB infections. The three compounds with better lipophilicity and electron-withdrawing groups showed > 200-fold higher efficacies than the parent drug.

**Scheme 80:**
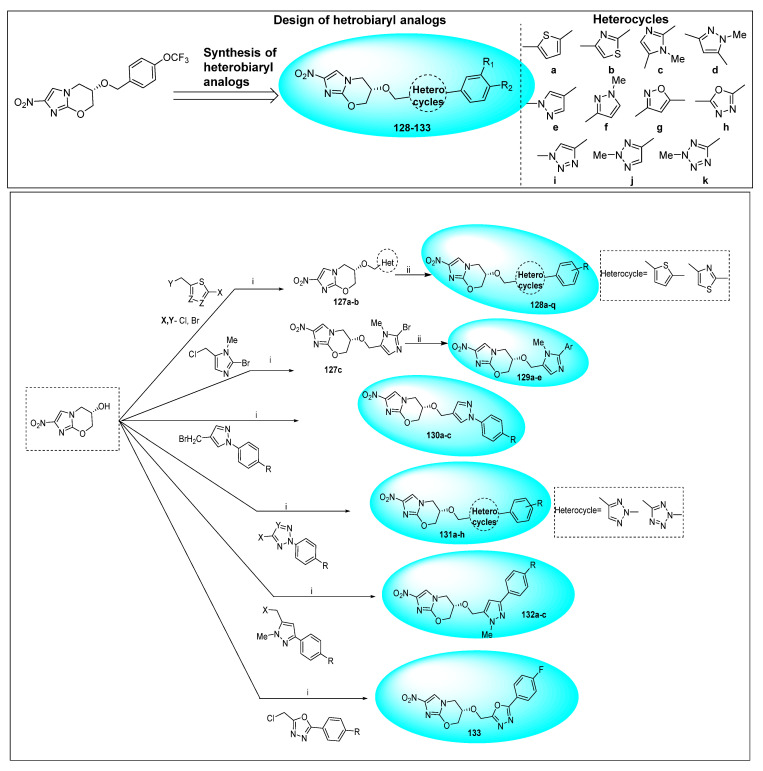
Synthesis of PA-824 **19** and its derivatives (Sutherland et al.). Reagents and conditions: (**i**) NaH, DMF, 0 °C, 2 h, B-26–54%, C-71–73%, D-81–94%, E-69–85%, F-36%; (**ii**) ArB(OH)_2_, toluene, EtOH, K_2_CO_3_, PdCl_2_(dppf), N_2_, reflux, A-51–67%; (**iii**) SOCl_2_, reflux, 0.5 h, B-83–93%; (**iv**) NaOAc, aq. AcOH, 100 °C, 15 h, 46–94%; (**v**) isoamyl nitrite, THF, reflux, 20 h followed by LiAlH_4_, Et_2_O, 0 °C, 20 h, and then PBr_3_, Et_2_O, 0–20 °C, 2–17 h, 12–81%; (**vi**) EtOH, 70 °C, 0.5 h, 41–78%; (**vii**) acetic anhydride, 20 °C, 0.5 h, Cs_2_CO_3_, THF, 20 °C, and then (CH_2_O)n, 2M HCl, reflux, 2 h, and then NaBH_4_, MeOH, 20 °C, 0.5 h followed by addition of PBr_3_, Et_2_O, 0–20 °C, 15–16 h, 51–67%; (**viii**) methylhydrazine, H_2_SO_4_, aq. NaHCO_3,_ CuI, PdCl_2_(PPh_3_) THF, 20 °C, 48 h under CO, and then HCl, THF, 80 °C, 16h followed by addition of PBr_3_, Et_2_O, 0–20 °C, 16 h, 69–85%; (**ix**) oxalyl chloride, ethylacetate, reflux, 30 min, followed by addition of POCl_3_, reflux, 2 h, 74%.

Sutherland et al. (2010) developed a new series of 2-nitroimidazooxazine bearing heterocyclic side chains as anti-TB agents (Scheme 80) [191]. After developing biphenyl analogs, the authors had designed the heterobiaryl analogs where 5-membered heterocycles replaced the phenyl ring. The compounds were constructed by the coupling of 2-nitroimidazooxazine with the halo partner of heteroaryl halides. The aryl heterocyclic compounds showed the most potent activity against replicating M. tb. while having improved solubility profiles. Among the synthesized compounds, two compounds with a pyrazole ring showed >10-fold more efficacy than the parent drug in the acute infection model.

**Scheme 81:**
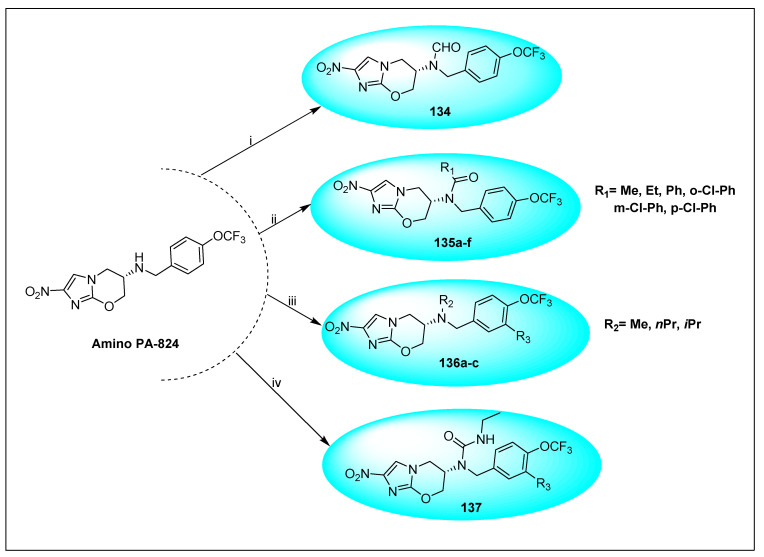
Synthesis of PA-824 **19** and its derivatives (Berry et al.). Reagents and conditions: (**i**) HCO_2_H, Ac_2_O, THF, 0 °C, 1 h, 55%; (**ii**) R_1_COCl, NaH, DMF rt to 70 °C, 48–55%; (**iii**) R_2_CHO, NaBH(OAc)_3_, MeOH/AcOH, 27–64%; (**iv**) triphosgene, EtNH_2_.HCl, Et_3_N, THF, 0 °C to rt, 66%.

**Scheme 82:**
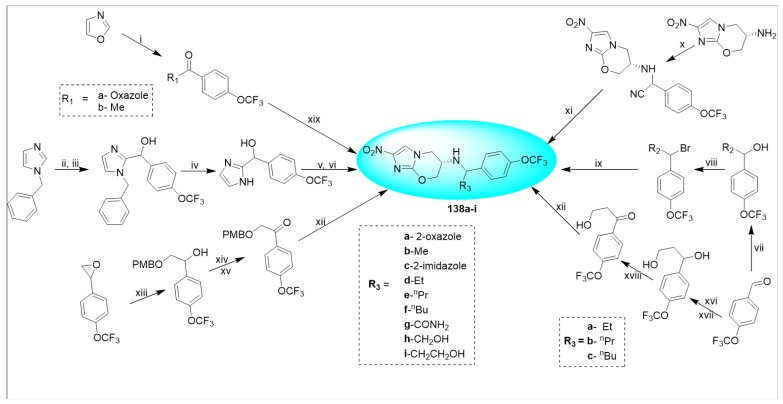
Synthesis of PA-824 **19** and its derivatives (Berry et al.). Reaction conditions: (**i**) *n*-BuLi, ZnCl_2_, CuI, THF, −78 °C to rt, 1.5 h, and then 4-trifluoromethoxybenzoyl-chloride, rt, 1 h, 40%; (**ii**) diisopropylcarbamyl chloride, DIPEA, 4-trifluoromethoxybenzaldehyde, CH_3_CN, reflux, 19 h, 73%; (**iii**) 50% TFA in water, THF, reflux, 15 h, 82%; (**iv**) H_2_, Pd/C, MeOH, 1 atm, 81%; (**v**) MsCl, Et_3_N, CH_2_Cl_2_, rt, 1 h (yield not reported); (**vi**) 2-nitro-6,7-dihydro-5*H*-imidazo[2,1-*b*][1,3]oxazin-6-amine, NaH, THF, rt, 40 h, 15%; (**vii**) RMgBr, THF, 0 °C to rt when R = Et and n-Pr; *n*-BuLi, THF, −78 °C to rt when R = *n*-Bu (yield not reported); (**viii**) PBr_3_, ether, 0 °C to rt (yield not reported); (**ix**) 2-nitro-6,7-dihydro-5*H*-imidazo[2,1-*b*][1,3]oxazin-6-amine, K_2_CO_3_, DMF, KI, 90 °C (yield not reported); (**x**) 4-trifluoromethoxybenzaldehyde, neat, 100 °C, 5 min, and then TMSCN, 100 °C, 30 min, 50%; (**xi**) EtOH/HCl, −10 °C, 38%; (**xii**) 2-nitro-6,7-dihydro-5*H*-imidazo[2,1-*b*][1,3]oxazin-6-amine, NaCNBH_3_, AcOH, EtOH, 5%; (**xiii**) 4-methoxybenzylalcohol, KO*t*Bu, 60 °C, 2 h, 34%; (**xiv**) PDC, CH_2_Cl_2_, rt, 24 h, 62%; (**xv**) TBDMSOTf, CH_2_Cl_2_, rt, 5 min, 86%; (**xvi**) ethylbromoacetate, Zn, CH_2_Cl_2_, rt, 3 h; (**xvii**) LiAlH_4_, THF, 0 °C to rt, 2 h, 25% over two steps; (**xviii**) MnO_2_, CH_2_Cl_2_, rt, 6 h, 50%; (**xix**) 2-nitro-6,7-dihydro-5*H*-imidazo[2,1-*b*][1,3]oxazin-6-amine, Ti(iOPr)_4_, AcOH, NaBH_3_CN, 9%.

**Scheme 83:**
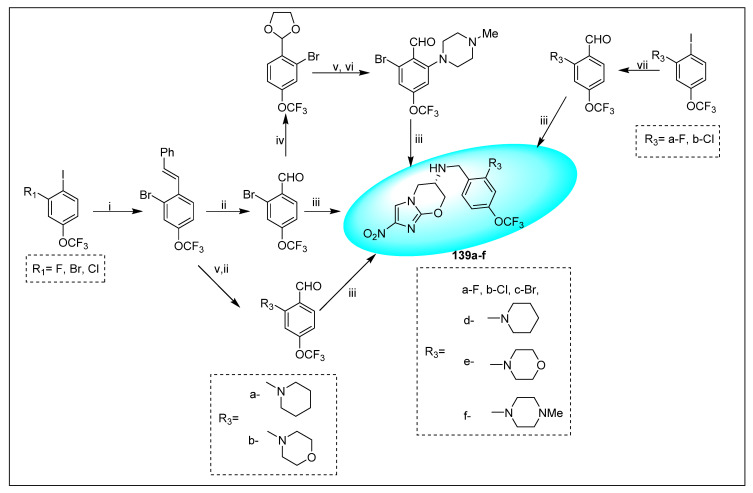
Synthesis of PA-824 **19** and its derivatives (Berry et al.). Reagents and conditions: (**i**) styrene, Pd(OAc)_2_, Et_3_N, 95 °C,16 h, 64%; (**ii**) OsO_4_, NaIO_4_, acetone-water, rt, 16 h, 16%; (**iii**) 2-nitro-6,7-dihydro-5*H*-imidazo[2,1-*b*][1,3]oxazin-6-amine, NaCNBH_3_, AcOH, DMF, 20 h, 13–72%; (**iv**) ethylene glycol, *p*-TSA, benzene, 80 °C, 8 h, 86%; (**v**) Pd(OAc)_2_, Cs_2_CO_3_, Xantphos, dioxane, amine, 90 °C, 8 h, 18-31%; (**vi**) THF, 6 N HCl, 30 min, rt, 55%; (**vii**) *n*-BuLi, THF, −78 °C**,** DMF, 15 min, 57–72%.

Cherian et al. (2011) developed the newer generation analogs by exploring the linker and lipophilic tail of PA-824 **19** (Scheme 81, Scheme 82 and Scheme 83) [192]. In this, the authors had performed the modification by incorporating “*N*” instead of “*O*”. The new generation analogs had been synthesized by introducing substitution at the third position of 4-trifluoromethoxybenzylamino tail, which was evaluated against replicating as well as non-replicating strains of MTB. Most of the compounds had shown better activity as compared with the parent molecule.

## 3. Mutagenicity and Genotoxicity of Selected Nitroimidazole Derivatives

**Figure 26:**
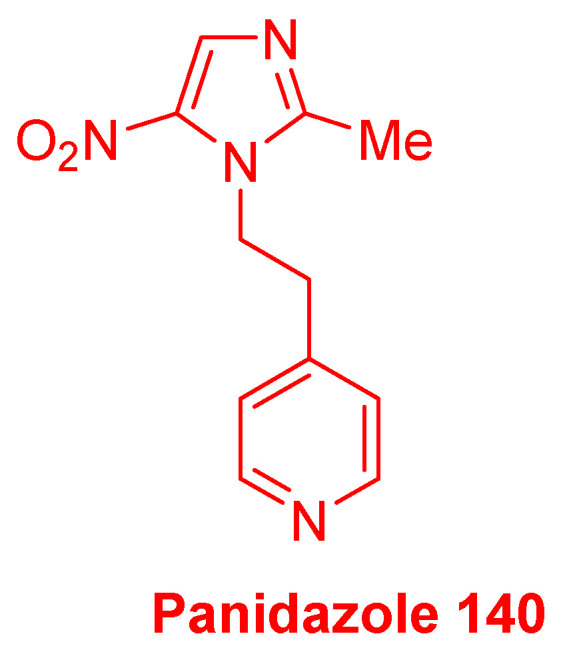
Chemical structure of Panidazole **140**.

Nitroimidazole derivatives are well known for their therapeutic effect through nitro group reduction [38]. However, the same is also responsible for mutagenic, genotoxic, and cytotoxic properties [30]. Metronidazole is relatively well tolerated in animals, and no signs of chronic toxicity problems were observed in rats [193]. In humans, metronidazole is also well tolerated and is used in pregnant women [194]. Several nitroimidazoles possess good oral therapeutic activity, but there are concerns with toxicity related to mutagenicity, especially if base-pair tester strains are used and if bacterial nitroreductases are present. Therefore, genotoxicity has made this drug development problematic [195]. These toxicity properties were related to DNA damage by the products of the bio-reduction of the nitro group. Consequently, positive Ames tests were observed, for instance, for the 5-nitroimidazole derivatives such as metronidazole, secnidazole, tinidazole, ornidazole, carnidazole and panidazole (Structure shown in Figure 26) using *Salmonella typhimurium* [113]. Despite this, several of these compounds are used for the clinical treatment of bacterial and protozoal infections. In addition, one of the reasons for their use is that the mutagenicity is different in mammalian cells, bacteria, and protozoa, which is discussed in the below subsection [196].

### 3.1. Azomycin 1

Voogd et al. studied the mutagenic properties of Azomycin **1** in *Klebsiella pneumonia* using Luria and Delbruck’s fluctuation tests. The mutagenic potential was studied in two concentrations of 0.5 and 0.25 mM, where mutation frequency was measured. In the studied method, azomycin did not show any increase in mutation frequency and was found to be non-mutagenic [197]. In this paper, the authors made a comparison of Azomycin with 2-nitroimidazole derivatives having a side chain at *N*-atom. The substituted derivatives have shown potential mutagenic activity.

### 3.2. Benznidazole 2

The mutagenic activity of Benznidazole **2** was studied in a plate incorporation assay using *Salmonella typhimurium* TA100 and its nitroreductase-deficient strain, TA100 NR, under aerobic or anaerobic conditions as well as with/without the addition of liver extracts. Benznidazole **2** has shown significant mutagenic activity in the *Salmonella typhi* TA100 strain under both aerobic and anaerobic conditions; however, the addition of liver enzyme did not alter any effects. Conversely, Benznidazole **2** did not show any mutagenic activity in the nitroreductase-deficient strain, TA100 NR, under aerobic conditions. These results revealed that Benznidazole **2** becomes mutagenic to a mammalian system under anaerobic conditions, and such environments are not expected to occur in most mammalian tissues [198]. Later on, Buschini et al. studied the mutagenic activity of Benznidazole **2** in *Salmonella strains* (TA100, TA100NR, TA98 and TA98NR) and revealed that Benznidazole **2** is more active for base-pair substitution than frameshift induction [199].

The genotoxic potential of Benznidazole **2** was evaluated by Comet assay test and micronucleus assay by Buschini et al [200]. The Comet assay was performed on fresh human leukocytes. Comet assay detected Benznidazole **2**-induced DNA damage at doses in the range of therapeutically treated patient plasma concentration and exerted its effect through ROS generation. In the micronucleus assay, Benznidazole **2** did not alter micronuclei frequency in the lower dose; however, it exerted its effects at higher doses and for a longer time (72 h). In the test assay, nifurtimox (NFX) was used as a comparator, showing its effects at lower concentrations in short duration assays (24 h).

### 3.3. Misonidazole 3

The mutagenic potential of Misonidazole **3** was studied by Chessin et al. using *E. coli* strain WP2 uvrA^-^, a tryptophan-requiring strain. Reversion to tryptophan independence on selective plates was used as the quantitative test of mutagenesis. In this study, the nitro containing drugs and leads (nitrofurantoin, Nifuroxime, Misonidazole **3**, Metronidazole **7**, NF-167, NF-269) were studied and was found that as the concentration of Misonidazole **3** was increased significantly, the frequency or survival of Trp+ revertants also increased significantly. These studies revealed that Misonidazole **3** induced mutation and was found to be mutagenic [201].

### 3.4. Azathioprine 6

In 1976, Herbold and Buselmaier studied the mutagenicity of azathioprine with liver microsomal assay by using different bacterial tester strains, namely *Salmonella typhimurium* TA1535, TA1536, TA1537, TA1538 and G46 [202]. It was observed that Azathioprine **6** produced negative results with TA1536, TA1537 and TA1538 in the frameshift strains of the test set, whereas a clear mutagenicity was demonstrated with TA 1535 and G46. Later on, Voogd et al. in 1979 studied the in-depth mutagenic action of Azathioprine using the fluctuation test of Luria and Delbruck using *Klebsiella pneumoniae* as a test organism [199]. In this study, Azathioprine **6** was found to be mutagenic even without metabolic activation at the concentration range from 2-0.1mmol/L. Similarly, Azathiopurine was compared with 6-mercaptopurine; it was interesting to observe that 6-mercaptopurine (0.6 mol/L) showed no mutagenic action on *K. pneumonia*, which indicates that this part of the azathioprine molecule does not contribute to the mutagenic activity. Later, Voogd reported that mutagenicity of Azathioprine for bacteria seems to be irrelevant for man because the nitroimidazole moiety can be reduced by bacteria but not by mammalian tissues [203].

The genotoxicity of Azathioprine **6** was investigated by performing a micronucleus test (mice/rats) and lymphocyte metaphase test (rabbits) [204]. In the micronucleus test, it was found that there was a dose-dependent increase in the number of cells with micronuclei. In the lymphocyte test, a dose of 20 and 5 mg per kg body weight was given orally to rabbits on three successive days after pertussis injection, where azathioprine induced a significant increase in cells with chromosomal abnormalities. The obtained results suggested that Azathioprine **6** is genotoxic.

### 3.5. Metronidazole 7

The mutagenicity of Metronidazole **7** was performed by Voogd et al. in the year 1974 through Luria and Delbruck’s fluctuation test using the following strains, *viz*., *Klebsiella pneumoniae*, *Escherichia coli* K_I2_ HfrH and *Citrobacter freundii* 425 with concentrations starting from 2–0.01 mM. Metronidazole **7** showed significant mutagenicity to *Klebsiella pneumonia* and *Escherichia coli*. It was found that 0.1 mM concentration of Metronidazole **7** induced 5.2- to 9.7-fold higher mutation than that of a spontaneous mutation rate, while the concentration of 1 mM increased the mutation rate by a factor of 39.6. When the mutagenic action of Metronidazole **7** was checked against Salmonella typhimurium TA 1530 and LT2, Voogd et al. found that it exerted clear mutagenic action and concluded that Metronidazole induced base-pair substitution mutation in TA 1530 and LT2 [205].

The genotoxic potential of Metronidazole **7** was evaluated by using the comet assay, micronucleus assay and chromosomal aberration tests. In Comet assay, metronidazole induced DNA damage in human lymphocytes (Comet assay), whereas in micronucleus assay, not much change was observed, making Metronidazole **7** non-genotoxic. However, in chromosomal aberrations in V79-379A cells, Metronidazole **7** exhibits a significant clastogenic action in hypoxic but not in aerobic cells [200,206].

### 3.6. Ornidazole 8

Voogd et al. in 1977 determined the mutation frequency of Ornidazole **8** in the Luria and Delbruck’s fluctuation tests using *Klebsiella pneumoniae* mutant requiring uracil and proline [207]. Ornidazole **8** has shown high mutagenic actions at a lower concentration; however, the effects are less at higher concentration levels. A possible explanation is also provided for the effects where an observed lower effect at the high concentration levels may be from the formation of charge-transfer complexes or other associations.

The genotoxic activity of Ornidazole **8** was evaluated in cultured human lymphocytes at various therapeutic concentrations [25]. The endpoints analyzed included: mitotic index (MI), replication index (RI), sister chromatid exchange (SCE) and chromosomal aberrations (CA). Among the endpoints describing genotoxic damage, a significant decrease in MI and an increase in SCE was observed in all cultures treated with Ornidazole **8**, and an increased percentage of cells with aberrations was observed during in vitro treatments. The analysis of chromosome aberrations also showed that most of the CA detected with Ornidazole **8** were chromatid breaks. These results suggested that Ornidazole **8** has a genotoxic effect.

### 3.7. Tinidazole 9

The mutagenicity of Tinidazole **9** was studied by Voogd et al. using Luria and Delbruck’s fluctuation test in the strains viz., *Klebsiella pneumoniae*, *Eischerchia coli* and *Citrobacter freundii* with a concentration ranging from 1 to 0.02 mM/l [207]. The mutation frequency of test organisms increased spontaneously on increasing the concentration of Tinidazole **9** from 0.05 to 1 mM/L. These results suggest its mutagenic nature; however, there is no linear relationship between the concentrations of the mutagenic agents and their mutation frequencies.

Nigro et al. studied the genotoxic activity of Tinidazole **9** in cultured human lymphocytes at various therapeutic concentrations and searched for the mitotic index (MI), replication index (RI), sister chromatid exchange (SCE) and chromosomal aberrations (CA). A significant decrease in the frequency of mitoses was observed, while a significant increase in the concentration of SCE was also observed. The analysis of chromosomal aberrations showed that most of the breaks detected were of the chromatid type. In addition, morphological changes in the nucleus and cytoplasm were observed in the treated cells. These results concluded that Tinidazole **9** is genotoxic, cytotoxic and is able to modulate cell death through apoptotic mechanisms [208].

### 3.8. Nimorazole 11

Nimorazole **11**, an antitrichomonal drug, also displayed a mutagenic effect. Voogd et al. determined the mutagenic potential of Nimorazole **11** using Luria and Delbruck’s fluctuation test. Nimorazole **11** had shown a dose-dependent response, and the effect was significant at higher concentrations [205].

### 3.9. Secnidazole 12

The mutagenicity of Secnidazole **12** was conducted in Ames assay using various strains of *Salmonella typhimurium* and one strain of *Eischerchia coli*. The concentration dose was chosen from 5000 to 33 µg/plate, and the number of revertant colonies also increased significantly. Thus, it was found to be mutagenic in these strains with and without the metabolic activation in rat liver extracts [94].

The genotoxic potential of Secnidazole **12** was evaluated through bone marrow micronucleus assay in Sprague–Dawley rats. There were no reductions in the ratio of PCEs to total erythrocytes. In addition, no significant increase in the incidence of mnPCEs was observed. According to the study conducted, it was concluded that Secnidazole **12** was negative in the rat micronucleus assay [94].

### 3.10. Dimetridazole 13

Luria and Delbruck’s fluctuation tests were carried out to evaluate the mutagenicity of Dimetridazole **13**. The test was carried out on different organisms such as *Klebsiella pneumoniae*, *Escherichia coli*, and *Citrobacter freundii* at different concentrations ranging from 1 to 0.01 mM. Dimetridazole **13** was found to be less active in the test assay in comparison to other nitroimidazole. With Klebsiella pneumoniae, solutions of 0.1 mM of Dimetridazole **13** increased the mutation rate by a factor of 3.4 to 4.1, whereas 1 mM solutions raised it to 32.2. Similar results were obtained with the two other test organisms *Escherichia coli* and *Citrobacter freundii.* Later on, the mutagenic action of Dimetridazole **13** was tested against different strains of *S. typhimurium*, which showed that Dimetridazole **13** also exerted a clear mutagenic action upon these Salmonella strains. With strains his G46 and TA 1530, a possible effect on the uvr repair system was detected. It may be concluded that the substances investigated induced base-pair substitution mutations in TA 1530 and his G46. The possibility of frame-shift mutations was investigated with strains TA I531, TA 1532 and TA 1534 of *S. typhimurium*. It shows that Dimetridazole also induced frame-shift mutations [199,205].

The genotoxicity of Dimetridazole **13** has been evaluated in human lymphocytes using the comet assay by Re et al. [206]. The test has been performed using three doses of 70.9, 212.6 and 354.3 mM under three experimental protocols: aerobiosis, anaerobiosis (90% N_2_, 10% CO_2_) and with the presence of the microsomal fraction S9 mix. In this study, the protective effects of four antioxidants, 8-hydroxyquinoline (8HQ), vitamin C (VitC), catalase (CAT) and superoxide dismutase (SOD), have been investigated on DNA damage generated by fixed concentrations of Dimetridazole **13** 354.4 mM. In aerobic conditions, Dimetridazole **13** produced significant dose–response relationships. The dose-related effects of the drug decreased or were abolished in anaerobic conditions or in the presence of S9 mix. 8HQ, VitC, CAT and SOD induced dose-related protective responses against DNA damage due to Dimetridazole **13**. These findings suggest that Dimetridazole **13** induced DNA damage in human lymphocytes through the futile cycle.

### 3.11. Fexinidazole 14

The mutagenic activity of Fexinidazole **14** was determined through Ames test using different strains of *Salmonella typhimurium*. Fexinidazole **14** has shown mutagenicity in all strains with different degrees. In each case, mutagenicity was either lost (e.g., TA98NR versus TA98) or significantly attenuated (e.g., TA100NR versus TA100) in the strains deficient in nitroreductase compared to their nitoreductase-proficient counterpart. In most cases, where mutagenicity was observed, potency of the signal was increased in the presence of rat liver S9 [209].

Fexindazole **14** was tested for by in vitro micronucleus tests using human peripheral lymphocytes. It did not induce chromosomal damage in human lymphocytes under these conditions. In addition, Fexinidazole **14** was tested for by in vivo mouse bone marrow micronucleus test, where the percentage of polychromatic erythrocytes of the total of erythrocytes in each bone marrow sample was used to estimate toxicity. The results revealed that Fexinidazole **14** does not induce any chromosomal damage in human lymphocytes. Similarly, in in vivo assay in the rat, no genetic damage was detected in the jejunum or liver even at a dose six times higher than that possible in the mouse study. The study concluded that Fexinidazole **14** and its metabolites have low redox potentials and therefore do not show any effects in the battery of assays. Thus, Fexinidazole **14** does not pose a genotoxic hazard to patients, which therefore qualifies it as a drug candidate for therapeutic applications [209].

### 3.12. Megazol 15

The mutagenicity of Megazol **15** was also determined by Mello et al. in the year 2013 through Ames test using various strains of *Salmonella typhimurium* with the concentration ranging from 1.0 to 0 µg/plate with and without metabolic activation by rat liver microsomes and was found to be mutagenic to all these strains tested for mutagenicity. However, due to its mutagenic and carcinogenic activity related to the nitro group, megazol is not used clinically [210].

The genotoxicity of Megazol **15** was reported by Nesslany et al. in the year 2004 by performing in vitro human lymphocyte metaphase analyses with and without metabolic activation and in vivo micronucleus assay test in rats [211]. It showed significant clastogenic activity in the in vitro human lymphocyte metaphase analysis test, with and without metabolic activation, and can therefore be considered as a clastogenic agent in cultured human lymphocytes. However, no numerical aberrations were observed despite the presence of structural aberrations and complex exchanges. Without metabolic activation in the 4 h treatment assay with one sampling at 20 h after the start of treatment, Megazol induced a dose-related increase in the number of chromosomal aberrations. At the highest dose tested of 0.625 mM, which is the maximum dose compatible with its cytotoxicity, a statistically significant increase in the number of breaks per cell and in the frequency of aberrant cells was observed. At the two lower doses of 0.312 and 0.156 mM, a slight increase in the number of aberrant cells was found, but this increase was not statistically significant. In the second assays without metabolic activation, a dose-related increase in the number of chromosomal aberrations was observed with a statistically significant increase in the number of breaks per cell and in the frequency of aberrant cells excluding or including cells with gaps only, at the three dose levels tested with the 20 h continuous treatment. In the in vivo micronucleus test in rats, a dramatic and statistically significant decrease in the ratio PCE to NCE was noted for the treatment group compared to the negative control group. Such an effect, reflecting a marked depression of the cell division in bone marrow, provides evidence that a sufficiently toxic dose had been administered and that exposure of bone marrow cells had taken place. A dark-yellow discoloration of the urine of treated animals was additional evidence of systemic exposure. Therefore, it was concluded that Megazol **15** displayed in vivo genotoxic activity after dosing by the oral route in the in vivo micronucleus rat bone marrow assay. Megazol **15** is a clear in vivo genotoxic compound, and as a consequence, it could be a human carcinogen.

### 3.13. CGI-17341 17

It has been found to be mutagenic in the Ames test, but no data were found [17].

### 3.14. Delamanid (OPC-67683) 18

The mutagenicity of Delamanid **18** was determined through Ames test using different strains of *Salmonella typhimurim* and *Escherichia coli*. The mutagenicity was evaluated both with and without the metabolic activation by rat liver extracts using the BRM test in accordance with OECD Guideline 471, and the number of revertants was counted 48 h after incubation at 37 °C. The selected dose range varied from 0 to 5000 µg/plate, and the number of revertants did not increase much, which revealed that Delamanid **18** is non-mutagenic [171].

Again in 2017, Matsumotu et al. examined the initial metabolic rate and mutagenic-specific activity of a series of nitro compounds in *S. typhimurium* TA100. The higher the metabolism (reduction), the higher the mutagenicity potential, but Delamanid **18** was not metabolized even after 60 h of treatment. In addition, Delamanid **18** was not reduced by two human nitroreductases. Thus, it was concluded that Delamanid **18** is devoid of genotoxicity in both in vitro and in vivo tests [212].

### 3.15. Pretomanid (PA-824) 19

The mutagenic activity of Pretomanid **19** was also determined through Ames test. The test organisms used were *Salmonella typhimurium* (different strains) and *Escherichia coli*. The mutagenicity was checked at different concentrations of the drug. PA-824 **19** was positive for mutagenic potential in the bacterial reverse mutation assay. Revertants were increased with TA100, TA1535 and WP2uvrA in the presence and absence of S9 mix. In addition, revertants were increased with TA98 in the presence of S9 mix and with TA1537 in the absence of S9 mix, suggesting that PA-824 **19** has some mutagenic properties [31].

### 3.16. VL-2098 20

Mukavilli et al. in 2014 have found VL-2098 **20** to be non- mutagenic in the **Ames** test, micronucleus and chromosomal aberration assay [186].

### 3.17. Nitroimidazole Derivatives Mutagenicity and Genotoxicity SARS: Selected Examples

**Figure 27:**
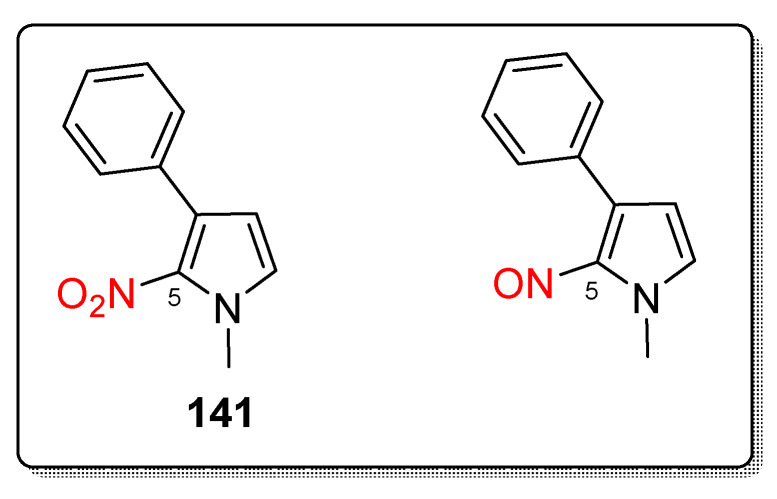
Chemical structure of 1-methyl-4-phenyl-5-nitroimidazole (**141**) and 1-methyl-4-phenyl-5-nitrosoimidazole (**142**).

Ehlhardt et al. demonstrated that 1-methyl-4-phenyl-5-nitroimidazole (**141**) is at least 1000-fold less cytotoxic for CHO cells and mutagenic for Ames tester strain TA100 than its corresponding homologous nitroso compound, 1-methyl-4-phenyl-5-nitrosoimidazole (**142**, Figure 27) [213]. The authors suggested that unlike the nitroimidazoles, the enhanced bactericidal activity of nitrosoimidazoles is expressed under both aerobic and anaerobic conditions resulting in nitrosoimidazoles that are more proximate to a common reactive species.

**Figure 28:**
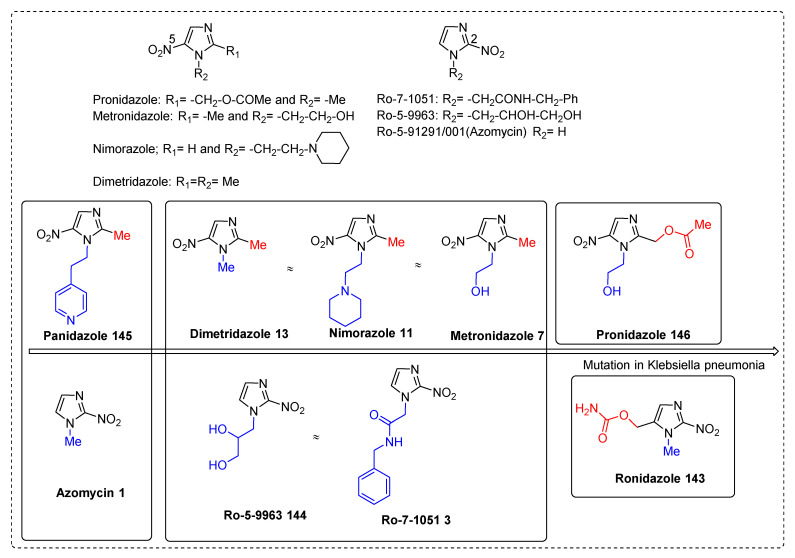
Mutagenic properties of a series of two substituted 5-nitroimidazole derivatives.

Another study performed by Voogd et al., as shown in Figure 28, summarizes the increase in mutagenic action of several 2- and 5-nitroimidazoles against *Klebsiella pneumoniae* [197,205]. Among the nitroimidazole derivatives, Ronidazole **143** showed the highest mutagenic activity, increasing the mutation rate of *Klebsiella pneumoniae*, whereas Metronidazole **7**, Nimorazole **11**, Dimetridazole **13**, Ro-7-1051 (misoindazole **3**) and Ro-5-9963 **144** displayed similar activities. Without any substituent on the nitroimidazole ring, no mutation rate was observed such as with Azomycin **1** as well as with the substituted 5-nitroimidazole Panidazole **145 [214]**.

**Figure 29:**
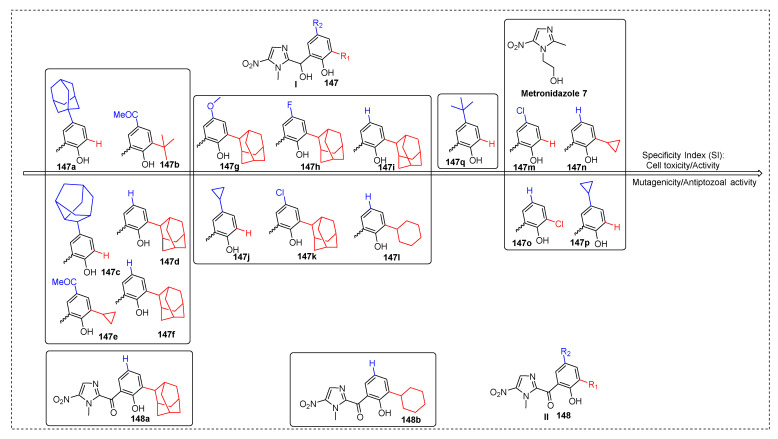
Mutagenic properties of two substituted 5-nitroimidazole derivatives.

Arredondo et al. prepared and tested for their antimicrobial activity as well as for mutagenicity (*Salmonella typhimurium* reverse mutation). To achieve this, series of 21 1-methyl-2-substituted 5-nitroimidazole derivatives were synthesized with the general formulas I (**147**) and II (**148**) compared with metronidazole (Figure 29) [215].

At a glance, the introduction of a bulky group in R_1_ or R_2_ decreased the mutations in *Salmonella typhimurium.*

**Figure 30:**
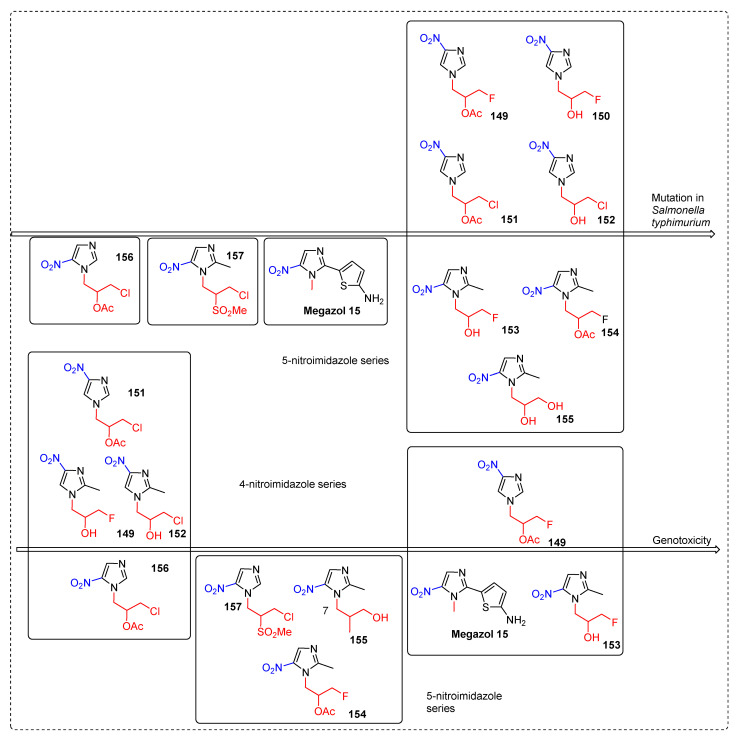
Genotoxicity (comet assay) and mutagenicity (*Salmonella typhimurium*) of nitroimidazoles.

Boechat et al. synthesized a series of ten 4- and 5-nitroimidazoles, including megazole **15**, bearing different substituent moieties that were investigated for their potential induction of genotoxicity (Comet assay) and mutagenicity (*Salmonella typhimurium*) (Figure 30) [30]. The 4-nitroimidazole derivatives **150** and **152** were not genotoxic versus the 5-nitroimidazoles **153**, **154** and **155**. The same result was observed with the 4-nitroimidazole **150** and the corresponding 5-nitroimidazole **148**, which is genotoxic. No influence on genotoxicity was observed regarding the position of the nitro group without methyl moiety in position 2, compound **151** versus **156**. Generally, the introduction of a fluorine atom induced genotoxicity (**153** versus **155**, **154** versus **156**, **149** versus **151**).

The mutagenic and genotoxic properties of various forty-eight 5-nitroimidazoles derivatives including Metronidazole **7** and Dimetridazole **13** have been evaluated using Ames test and the SOS Chromotest (Table 5). In the Ames test, *Salmonella typhimurium* strain TA 100 was used with and without metabolic activation, whereas Escherichia coli strain PQ 37 was used with and without metabolic activation in the SOS Chromotest [216]. Forty-five nitroimidazoles showed mutagenic and genotoxic properties, whereas three molecules showed neither mutagenic nor genotoxic activity with and without the metabolic fraction. Good correlation was observed between the mutagenic potencies (MP) and the SOS induction powers (SOSIP) without the S9: log(MP) = 0.88 X log (SOSIP) + 2.675 with r = 0.845 (*n* = 84). As shown in Table 4, compound **157** displayed the highest MP and SOSIP, whereas the transfer of the nitro group from possition 5 at position 4 decreased the mutagenic potency, which became similar to that of dimetridazole. The replacement of the nitro moiety by a cyano group also decreased the mutagenicity.

**Figure 31:**
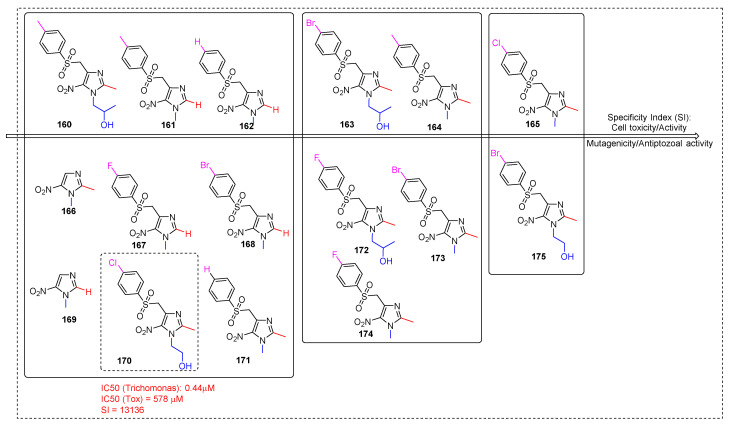
Cytotoxic activity and the antitrichomonas activity of 5-nitroimidazole derivatives.

Twenty-four antiprotozoal 5-nitroimidazole derivatives bearing an arylsulfonylmethyl group were prepared by M. D. Crozet et al. [155]. In vitro antiparasitic activity was determined against trichomonas vaginalis, whereas in vitro mutagenicity was evaluated by the *Salmonella* mutagenicity assay. All the tested molecules were mutagenic in the *Salmonella* mutagenicity assay using the most sensitive strain, TA100. The tested compounds showed better activity against *T. vaginalis* than metronidazole. Good correlation was observed between the antiprotozoal activity and the mutagenicity for the 21 compounds, reflecting their ability to damage DNA through covalent binding and induction of DNA breaks. 5-Nitroimidazole derivatives with an additional methyl group on the 2 position displayed a lower mutagenicity than Metronidazole. Moreover, 11 derivatives had an SI over the one of metronidazole, and compound **170** showed an SI of 13136 (Figure 31).

**Figure 32:**
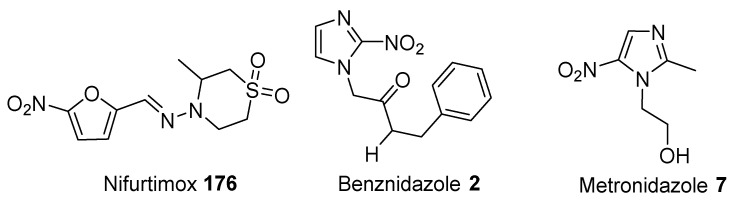
Chemical structure of Nifurtimox **176**, Benznidazole **2**, and Metronidazole **7**.

Buschini et al. analyzed the genotoxicity of Nifurtimox **176,** Benznidazole **2**, and Metronidazole **7** (Figure 32) [200].

Benznidazole 2 was evaluated by both comet and MN assay. Discrepancies between the results obtained by the Comet and MN tests were observed. Comet assay, at a low concentration, displayed significantly increased DNA damage. No increase in chromosomal damage was detected by MN assay at low concentrations. Importantly, nifurtimox and benznidazole are more mutagenic than metronidazole **7**. These two compounds induced DNA damage at doses in the range of therapeutically treated patient plasma concentrations through ROS generation and dose-dependent mechanisms of DNA damage for Benznidazole **2** and Nifurtimox **176**, respectively. For metronidazole, no effects on mammalian cells were observed, whereas MN induction is observed for Nifurtimox **176** and Benznidazole **2.** The effects with metronidazole **7** are dependent on anaerobic/hypoxic conditions, whereas for Nifurtimox **176** and Benznidazole **2**, interaction with the DNA of mammalian cells and cellular damage are the two processes regarding their toxicities.

## 4. Conclusions and Future Perspectives

Nitroimidazoles and their derivatives have drawn continuing interest over the years because of their varied biological activities. In 1953, Maeda et al. discovered the first nitroimidazole with anti-bacterial activity. Then, numerous nitroimidazole derivatives were prepared and developed, with remarkable broad-spectrum activity, as anti-bacterial, anti-cancer, anti-HIV, anti-parasitic, anti-tuberculosis, anti-leishmaniasis agents, etc.

In short, nitroimidazole-based drugs and leads are better defined as prodrug, and their bio-activation utilizing the nitro functionality is the major reason for their mechanisms of action. In addition to this, the position of the nitro group also plays an important role for bio-activation, which is activated differently by diverse conditions (redox potential of host and parasites based upon the diseases) and is the reason for activity against diverse disease conditions. In general, each class has shown some trends against the disease conditions. Among the four subclasses discussed in the review, (i) 2-nitroimidazole-based drugs are known as anti-protozoal agents and radio-sensitizers agents, (ii) 4-nitroimidazole-based drugs are used as an immunosuppressive drug, (iii) 5-nitroimidazole-based are known for anti-bacterial and anti-parasitic properties, and (iv) fused nitroimidazole-based drugs were exploited as anti-tubercular agents; however, the recent study also reported them as anti-leishmanial and as anti-parasitic agents. More robust strategies and approaches are still required to understand the exact mechanisms of action for each of the classes discussed, which will definitely open new opportunities to bring more effective drugs into this class. Nitroimidazoles can target a broad range of parasitic and bacterial pathogens that infect different sites within the human body, where no other drug classes are effective. There has been renewed interest, and in the last decade, nitroimidazole derivatives belonging to bicyclic-fused nitroimidazole have also shown great potential in TB drug discovery.

Here, we have compiled and summarized mutagenic data (bacterial reverse mutation assay) and genotoxicity (Comet assay, chromosomal aberrations assay under in vitro conditions and micronucleus assay under in vivo conditions) of nitroimidazole-based drugs available in the literature. Although the different assays were used for screening, a unique trend is developing for their mutagenic and genotoxic profile. The Ames test data (using different bacterial strains) of nitroimidazole-based drugs belonging to 2-.4-,5- and fused nitroimidazoles revealed that most of the drug candidates have mutagenic potential except for Azomycin **1**, Delamanid **18** and VL-2098 **20**. Azomycin **1** is a 2-nitroimidazole and represents the first molecule discovered in the scaffold and is without any substitution. Afterward, several other drugs were synthesized with diverse functionality. Delamanid and VL-2098 **20**, belonging to fused nitroimidazole, were presented as recent discoveries, where the former is approved as a drug against MDR-TB and the latter is presented as a potent pre-clinical anti-leishmanial candidate. Among all the drugs and leads, these two candidates are free of mutagenic and genotoxic liabilities, suggesting that introduction of a five-member cyclic ring helped the molecules to escape from nitroreduction by bacterial strains and mammalian cells, which is responsible for the mutagenicity and genotoxicity.

**Figure 33:**
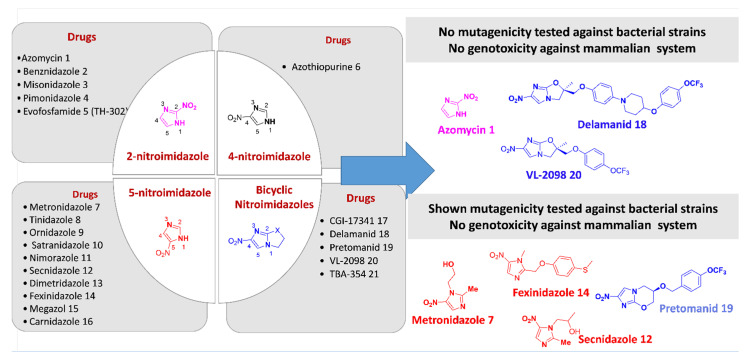
Summarized mutagenic trend in nitroimidazole scaffold.

Apart from these, another notable trend is also developing where some of the drug candidates are positive in bacterial Ames tests (Mutagen); however, they become negative in mammalian genotoxic assays (Figure 33). The overall effects of the candidates against all the assays are the deciding factors for the selection of drugs. The molecules that do not have any effect in the in vivo micronucleus assay are qualified for further development. Moreover, the dosage is also another important parameter in overall selection, if the molecules are active in higher concentrations, which should have good and acceptable fold selectivity and are considered for the overall selection. There are also candidates belonging to the nitroimidazole class, which are positive in in vitro assays but become negative in in vivo conditions. The molecules belonging to this category are Metronidazole **7**, Secnidazole **12**, Fexinidazole **14** and Pretomanid **19**. Metronidazole **7**, Secnidazole **12**, and Fexinidazole **14** belong to 5-nitroimidazole having di-substitutions, and conversely, Pretomanid **19** belong to fused nitroimidazole, having a fused six-membered ring that also helps the candidate to escape genotoxicity. In summary, the introduction of an additional ring and substitution helps nitroimidazole to escape from mutagenicity and genotoxicity. This vital information could be helpful for future design and discoveries.

The presence of a nitro group and its role in mutagenicity and genotoxicity is one of the major reasons for not actively pursuing this scaffold in drug discovery; however, the current understanding is providing a way to deal with such a problem. However, more design and effort are required to understand the balance between activity and toxicity. Considering the track record of nitroimidazole-based drugs to counter anaerobic and parasitic infections, nitroimidazole is still the choice to deal with the other related complications and diseases. Moreover, the multi-target engagement of nitroimidazole-based drugs also provides an opportunity for a comparatively low prevalence for drug resistance, which is one of the major concerns for anti-bacterial drug discovery. Computational, proteomics, bioinformatics and polypharmacological approaches can be used for future drug design. Finally, the development and expansion of the nitroimidazole family collection to address unmet needs in the area of neglected infectious diseases should be strongly encouraged.

## Figures and Tables

**Scheme 43:**
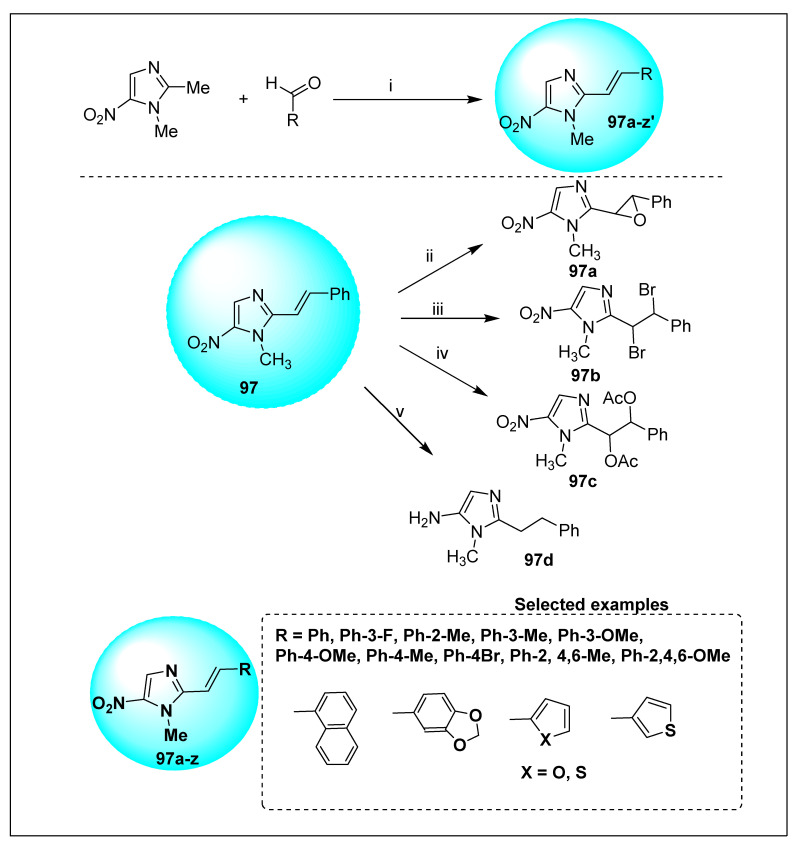
Synthesis of 5-nitroimidazole derivatives as antimicrobial agents. Reagents and conditions: (**i**) NaOEt, EtOH, reflux, 13–77% (**ii**) *m*-CPBA, DCM, 0 °C to rt, low yield; (**iii**) Br_2_, CHCl_3_, 88%; (**iv**) citric acid, NMO, K_2_OsO_4_.2H_2_O, *t*-BuOH/H_2_O, acetic anhydride, pyridine, 66%; (**v**) Pd/ H_2_, MeOH (yield not reported).

**Table 1:** Inhibition data of dual-function Misonidazole derivatives.

	Anti-Angiogenesis Activity			Radiosensitizing Activity
	Protease Inhibitory Activity	RLE Cell Proliferation Activity	Anti-Angiogenic Activity	Radiosensitizing Effect (10 μM)
Compound ID	K_I_ (μM)	IC_50_ (μM)	% Inhibition	ER
**27a** (TX-1863) ^a^	1180	325	80	
**27b** (TX-1866) ^a^	66	20	100	
**27c** (TX-1880) ^a^	260	71	55	1.76
**27d** (TX-1882) ^a^	42	6.3	61	1.82
**27e** (TX-1897) ^a^	30	7.5	64	1.73
**27f** (TX-1898) ^a^	9	1.4	93	1.80
**25a** (TX-1878) ^b^	1750	275	83	
**25b** (TX-1879) ^b^	220	17	100	
**25c** (TX-1881) ^b^	557	80	53	1.75
**25d** (TX-1883) ^b^	55	6.9	77	1.83
**25e** (TX-1899) ^b^	56	6.5	58	1.90/1.75 ^c^ (1 μM)
**25f** (TX-1900) ^b^	14	0.9	82	1.74

^a^ *R*-isomer; ^b^ *S*- isomer; ^c^ Measured at 1 μM because of cytotoxicity at 10 μM without irradiation; ER, radiosensitizing enhancement ratio; K_I_, inhibition constant.

**Table 2:** In vitro and in vivo antibacterial activities.

	In-Vitro Activity against Selected Organism MIC (µM)							
Compound	*S. aureus*	*S. pyrogens*	*C. perfringens*	*M. gallisepticum*	*T. vaginalis*	In Vivo Activity against *T. vaginalis*	Rel. ED_50_ mg/kg	LD_50_ mg/kg
**30**	25	200	0.8	50	25	>10	0.7	
**31**	100	100	>3.1	100	2	1.65	0.28	1969
**32**	>100	100	10	25	100	4.67	0.33	141
**33**	>100	>100	3.1	100	10	5.74	0.40	156
**34**	>100	100	3.1	25	100	28.83	2.88	
**35**	100	>100	>100	50	100	16.2	1.62	
**36**	>100	>100	5	100	100	>40		99
**Metronidazole 7**	>300	>300	0.6	>300	5	5.77	1	658
**Tinidazole 8**	>300	>300	0.3	>300	3.1	2.5	0.43	1280
**Nimorazole 11**	>300	>300	0.0	>300	12.5	37.9	4.66	40

**Table 3:** Various parameters of the hypoxia-selective cytotoxins **41** (NLQZ-1) and **42** (NLPP-1) and comparison with NLCQ-1 in V79 cells.

Comp.	IC_50(A)_^a^ (µmol/L h)	IC_50(H)_^a^ (µmol/L h)	HS ^b^	C_1.6_ ^c^ (µmol/L)	C_i1.6_^d^ (µmol/L)	ThI ^e^
**41** (NLQZ-1)	998.6–1486.7	73.6–108.4	14	61.4	347.9 ± 6.0	16–24
**42** (NLPP-1) (V79)	1000–962.2	86.1–51.9	12–19	75.0	271.9 ± 3.5	13
**42** NLPP-1 (A549)	1668.8–1311.6	97.6–59.7	17–22	44.1	ND	30–38
NLCQ-1	414.2–958.9 ^f^	86–14.7 ^f^	5–65 ^f^	7.2	62.5 ± 1.5	57–133 ^f^

^a^ The product of a compound’s concentration and the time necessary for 50% reduction in clonogenicity under aerobic (A) or hypoxic (H) conditions. Since cytotoxicity was exposure time dependent, range of values for 1–3 h exposure to each compound is shown. ^b^ Hypoxic selectivity: IC_50(A)_/IC_50(H)_. ^c^ Concentration for an SER of 1.6. SER, sensitization enhancement ratio. ^d^ Intracellular concentration at C_1.6_, determined as described in Materials and Methods. ^e^ In vitro therapeutic index: IC_50(A)_/C_1.6_. ^f^ Range of values for 1–4 h exposure is shown for NLCQ-1.

**Table 4:** In vitro and in vivo Anti-tubercular activity.

Sr. No.	R	R_1_	R_2_	Anti-Tubercular Activity	
				MIC μg/mL	In Vivo Efficacy mg/kg p.o ED_50_
**17a**	H	CH_3_	H	1.95	25
**17b (CGI-17341)**	H	C_2_H_5_	H	0.06	10
**17c**	H	CH_2_Cl	H	0.12	30–100
**17d**	H	Ph	H	0.95	Ф at 100
**17e**	H	Bu (n)	H		
**17f**	H	CH_2_Br	H	0.24	30–100
**17g**	H	CCl_3_	H	31.2	
**17h**	H	CH_2_OPr *(iso)*	H	3.9	Ф at 200
**17i**	H	CH_2_Oallyl	H	3.9	Ф at 200
**17j**	H	CH_2_OPh	H	0.24	Ф at 200
**17k**	H	Bu (n)	CH_3_	0.015	
**17l**	H	(C_7_H_15_)_n_	CH_3_	0.0037	
**17m**	H	(CH_2_)_5_	H	0.03	Ф at 100
**17n**	H	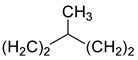	H	0.12	
**17o**	H	(CH_2_)_6_		0.05	Ф at 100
**17p**	H	(CH_2_)_4_	H	31.2	--

**Table 5:** Ames test and SOS Chronotests for Metronidazole **7** and Dimetridazole **13**, **157**, **158** and **159**.

Compounds	AMES Test Mutagenic Power		SOS Test Induction Power	
	−S9 + S9		−S9 + S9	
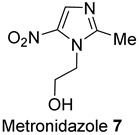	**10**	14	0.079	0.0210
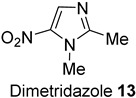	12	16	0.0221	0.0724
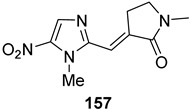	53,117	26,830	107	59.3
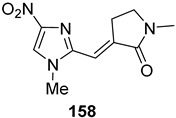	11	11	0.05	0.0118
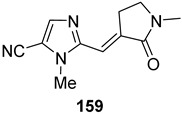	0.482	1.55	0.00426	<0.0010

## Data Availability

Data is contained within the article.

## Abbreviations

ADMET	absorption, distribution, metabolism, elimination, and toxicity
AUC_0-t_	area under the concentration–time curve from 0 to the last quantifiable concentration estimated by the trapezoidal rule
AUC_0-∞_	area under the curve extrapolated to infinity, after a single dose
BSA	body surface area
C_max_	maximum (or peak) serum concentration that a drug achieves
CL/F	oral clearance
Cl	rate at which the active drug is removed from the body
ELISA	enzyme-linked immunosorbent assay
FDA	Food and Drug Administration
F(%)	oral bioavailability
IC_90_	concentrations leading to relative effects of 10%
K_I_	inhibitory constant
MIC_50_	minimum inhibitory concentration (inhibition of 50%)
MIC_90_	minimum inhibitory concentration (inhibition of 90%)
MRT	mean residence time of drug in the body
PK	drug pharmacokinetics
R&D	research and drug development
T_max_	amount of time that a drug is present at the maximum concentration in serum
t_1/2_	time taken for levels of drug to decrease by half (a measure of the rate of elimination of the drug from plasma)
V/F	ratio of volume of distribution/bioavailability
V_d_	olume of distribution is the theoretical volume that would be necessary to contain the total amount of an administered drug at the same concentration that it is observed in the blood plasma
